# Regioselective Arene C–H Borylation with a MOF-Immobilized Iridium Bipyridine Catalyst

**DOI:** 10.1021/acsami.5c24764

**Published:** 2026-02-13

**Authors:** Jordon S. Hilliard, May M. Cheline, Andrew J. Robinson, Casey R. Wade

**Affiliations:** Department of Chemistry and Biochemistry, 2647 The Ohio State University, 100 West 18th Ave, Columbus, Ohio 43210, United States

**Keywords:** metal−organic frameworks, heterogeneous catalysis, C−H activation, borylation, regioselectivity

## Abstract

Postsynthetic ligand exchange and metalation have been used to immobilize iridium bipyridine precatalysts in MFU-4*l* with controlled loadings. The resulting materials, **1-Ir-** ***x*** (*x* = 0.1–0.5), exhibit high activity for catalytic C–H borylation of arenes, and **1-Ir-0.5** achieves up to 1560 turnover numbers per Ir for toluene substrate. Remarkably, **1-Ir-0.5** exhibits high *meta*:*para* product regioselectivity compared to a homogeneous catalyst analogue for arene substrates bearing bulky triisopropylsilyl groups. Density functional theory (DFT) calculations reveal that steric constraints imposed by metal-organic framework (MOF) pore confinement increase the free energy barrier for *para* C–H activation, biasing product formation toward the *meta* isomer.

## Graphical abstract

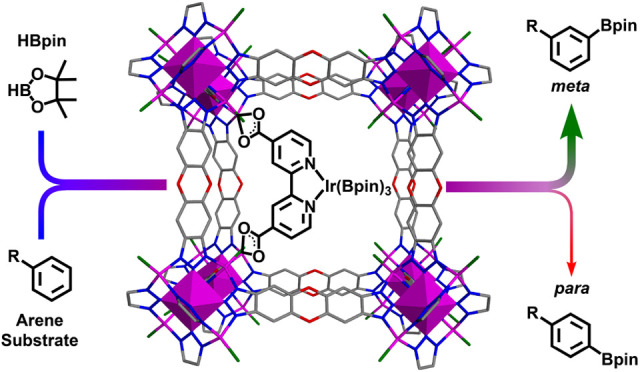

## Introduction

Catalytic C–H bond functionalization has emerged a powerful tool in modern synthetic chemistry. In particular, C–H borylation of arenes has received considerable attention for the synthesis of organoboron compounds that serve as key intermediates for cross-coupling reactions used in the synthesis of pharmaceuticals, agrochemicals, and advanced materials.^1–3^ Iridium-based complexes supported by 2,2′-bipyridine or 1,10-phenanthroline ligands are among the most well studied and commonly used catalysts.^4–6^ However, site-selective C–H borylation remains challenging for arene substrates that lack strong directing groups.^7–10^ Ligand design has enabled regioselective C–H activation with these systems. For example, catalyst–substrate interactions promoted by pendant functional groups capable of hydrogen bonding,^11–13^ ion pairing,^14–18^ or Lewis adduct formation^19–22^ have been shown to dramatically increase product regioselectivity. Sterically encumbering ligands that limit substrate access to the iridium site can also influence selectivity. Segawa, Itami, and co-workers used an iridium catalyst with a bulky diphosphine ligand (**A**) to facilitate *para*-selective C–H borylation of arenes (Figure 1).^23,24^ The large phosphine substituents block access to the *ortho*- and *meta*-C–H bonds of monofunctionalized arene substrates, resulting in high regioselectivity for *para* borylated products. Similarly, Asako, Ilies, and co-workers designed *spiro*-bipyridine ligands (**B**) with substituents that project above the iridium active site, providing a roof-like effect.^25^ This remote steric influence disfavors C–H activation at the *para* position of monofunctional arenes, resulting in high selectivity for *meta* borylation (Figure 1).

**Figure 1:**
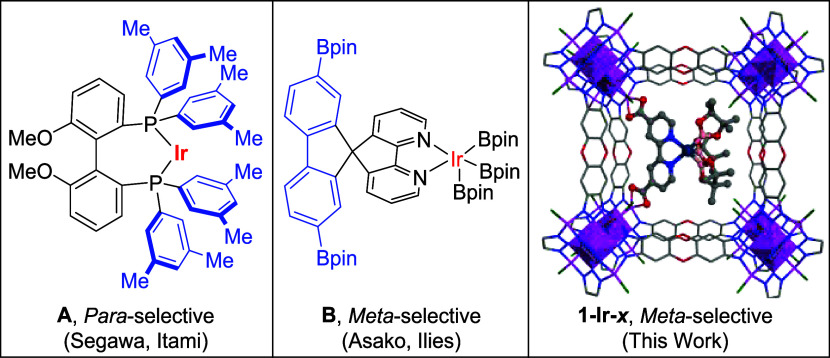
Structures of regioselective catalysts **A** and **B** bearing sterically encumbering ligands and MOF-based catalyst **1-Ir-** ***x***.

Metal–organic frameworks (MOFs) continue to attract significant interest as solid supports for heterogeneous catalysis. Catalyst recyclability and site-isolation effects that inhibit deactivation are widely touted benefits of MOF supports. Although less common, catalyst confinement and pore microenvironment effects have been observed to bias product regioselectivity.^26^ The latter has motivated us to investigate the possibility of using MOF-supported catalysts for regioselective C–H borylation of arenes. Previous studies with MOF-immobilized iridium catalysts, including those from our laboratory, have shown improved catalyst activity and recyclability.^27–35^ However, the reported substrate scopes for C–H borylation have generally been limited to small arene substrates, resulting in product regioselectivity that mirrors that of related homogeneous catalysts.

Herein, we describe the synthesis, characterization, and catalytic studies of an iridium bipyridine catalyst supported in MFU-4*l*.^36^ Ligand exchange at the terminal Zn–Cl sites of the MOF nodes allows facile immobilization of 2,2′-bipyridine-4,4′-dicarboxylate (bpydc^2–^) with controlled loadings (Scheme 1). Subsequent postsynthetic metalation with [Ir­(OMe)­(cod)]_2_ affords precatalysts (**1-Ir-** ***x***) that are highly active for C–H borylation of toluene. Remarkably, catalyst confinement also results in high selectivity for *meta* borylation of arene substrates bearing large triisopropylsilyl (TIPS) substituents. DFT calculations reveal that the product bias arises from confinement effects and the steric influence of the MOF pores.

**Scheme 1:**
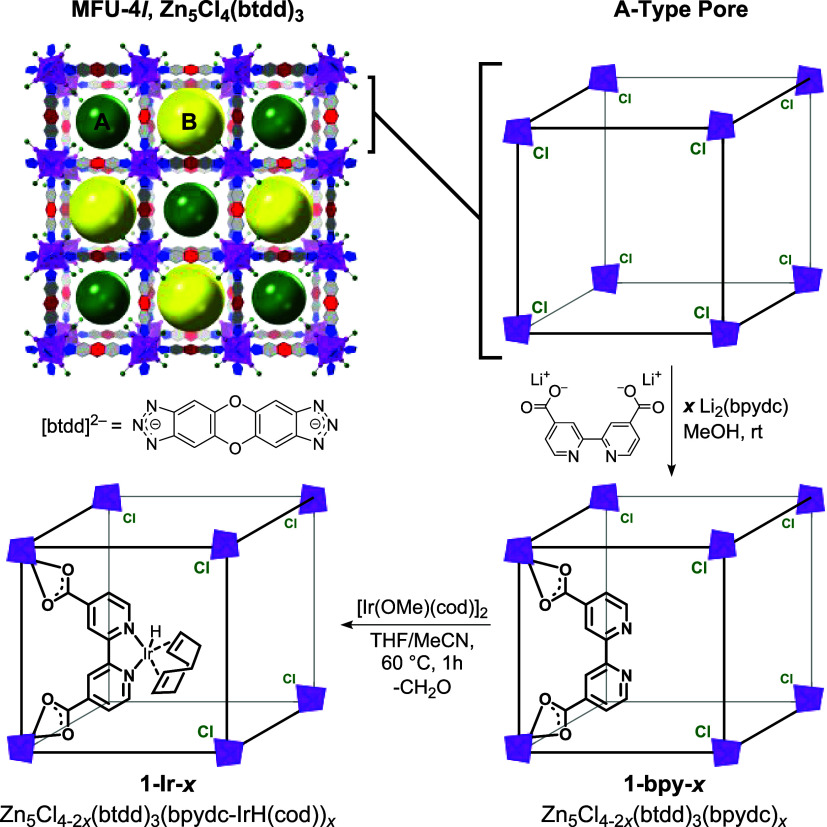
Postsynthetic Modification of MFU-4*l* to Generate **1-bpy-*x*** and **1-Ir-*x***

## Results and Discussion

The bipyridine functionalized MOFs **1-bpy-** ***x*** (*x* = 0.1, 0.2, 0.3, 0.4, 0.5) were prepared by treating MFU-4*l* with methanol solutions containing the corresponding amount of Li_2_(bpydc). ^1^H NMR spectra of the acid-digested products (Figures S1–S5) confirmed quantitative ligand exchange according to the empirical formula Zn_5_Cl_4–2*x*_(btdd)_3_(bpydc)_*x*_, where *x* represents the amount of bpydc incorporated per formula unit. Additionally, *x* = 0.5 corresponds to one bpydc per A-type pore, which is the upper limit for ligand immobilization in the MOF. PXRD analysis confirms that the MOFs retain crystallinity, and their structure is largely unchanged (Figure S8). N_2_ gas adsorption isotherms show a modest decrease in porosity and surface area as a function of increased bpydc loadings (Figure S9).

Activated samples of **1-bpy-** ***x*** were treated with an MeCN/THF (3:1 v/v) solution containing 0.5 equiv of [Ir­(OMe)­(cod)]_2_ (cod = 1,5-cyclooctadiene) per bipyridine and heated at 60 °C. The yellow color of the supernatant solutions gradually dissipated from yellow to colorless over the course of 1 h, signaling incorporation of [Ir­(OMe)­(cod)]_2_ into the framework. The solid MOF samples experienced an accompanying color change from beige to orange. The products, **1-Ir-** ***x***, were subsequently washed with acetonitrile to remove any unreacted [Ir­(OMe)­(cod)]_2_ and dried *in vacuo* at 45 °C. ICP-OES analysis is consistent with quantitative metalation of the bpydc ligands (Table S1). PXRD analysis confirms that the framework structures are retained after postsynthetic metalation while BET surface areas calculated from N_2_ gas adsorption isotherms show a monotonical decrease in porosity with increased catalyst loading (Figure 2). Pore size distributions calculated using 2D nonlocal density functional theory reveal a systematic decrease in size of the smaller A-Type pores with increased catalyst loading (Figure S10).^37^ CO adsorption isotherms (300 K) were also measured for the **1-Ir-** ***x*** series, revealing steep chemisorption profiles. The CO capacities at 50 mbar pressure correspond to binding of ∼2 CO molecules per iridium site (Figures 2c and S11). Accordingly, the attenuated total reflectance infrared (ATR-IR) spectrum of **1-Ir-0.3** measured after the CO adsorption experiment exhibits two v­(CO) bands at 2087 and 2020 cm^–1^. These bands correspond to v­(CO) symmetric and asymmetric stretching modes and are consistent with the formation of a five-coordinate *cis*-Ir­(CO)_2_ species (Figure S12).^38^

**Figure 2:**
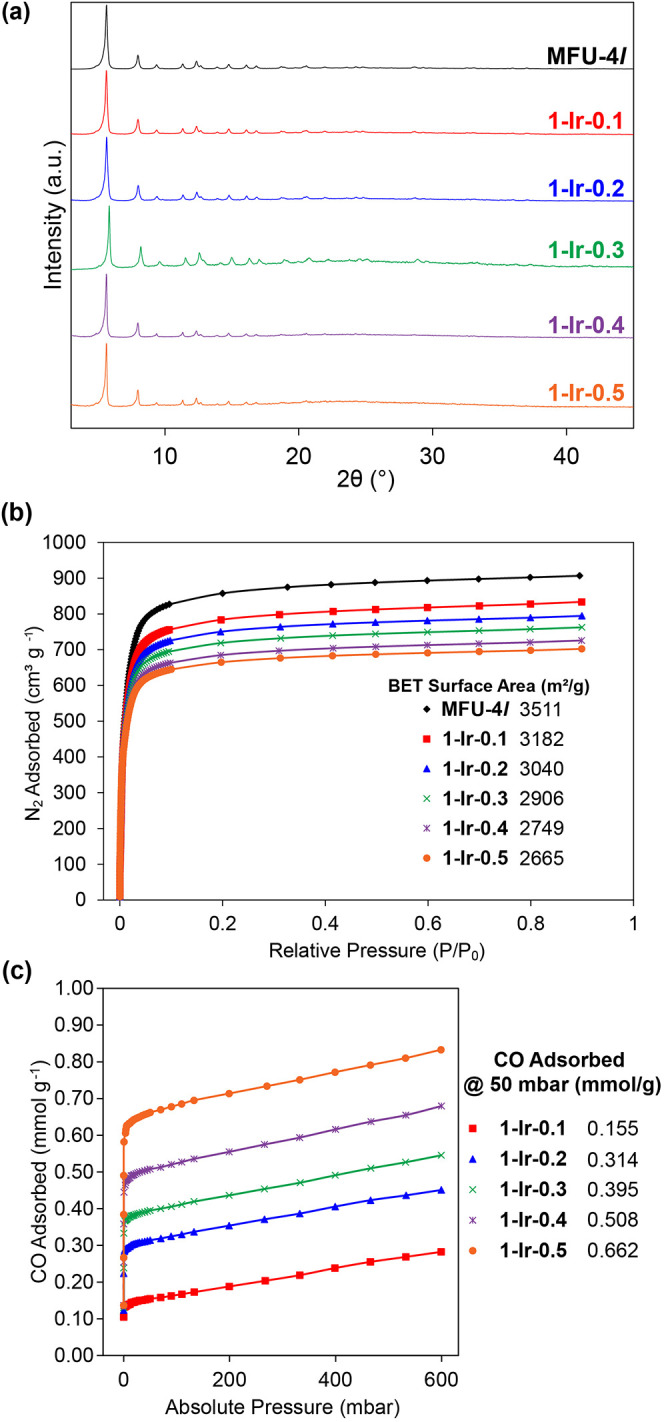
(a) Powder X-ray diffraction patterns, (b) N_2_ adsorption isotherms (77 K), and (c) CO adsorption isotherms (300 K) for the **1-Ir-** ***x*** MOFs.

Initial catalytic screening of the **1-Ir-** ***x*** MOFs for C–H borylation of arenes was carried out in neat toluene substrate with bis­(pinacolato)­diboron (B_2_pin_2_) as the borylating reagent. The catalyst loading was held constant at 0.5 mol % iridium with respect to B_2_pin_2_, taking into consideration the varying iridium site density (*x*) in the MOFs. Product formation was monitored by gas chromatography (GC-FID) and turnover numbers per iridium (TONs) were calculated with respect to an internal standard. TONs determined after 1 h reveal increasing activity as a function of catalyst site density with **1-Ir-0.5** reaching 355 TONs (Table 1, entries 1–5). In all cases, a ∼0.1:1:1 *ortho*:*meta*:*para* distribution of monoborylated products was observed. A series of control reactions were performed to interrogate the role of the immobilized bipyridine ligand in the **1-Ir-*x*** catalysts. A mixture of [Ir­(OMe)­(cod)]_2_ and MFU-4*l*-Cl yielded only 12 TON after 20 h (Table 1, entry 7), indicating that the immobilized bipyridine ligand plays an important role in generating catalytically active species. A homogeneous control reaction employing [Ir­(OMe)­(cod)]_2_ and 4,4′-di-*tert*-butyl-2,2′-bipyridine (dtbpy) gave 252 TON after 1h (Table 1, entry 8). The higher catalytic activity of **1-Ir-0.4** and **1-Ir-0.5** in comparison to the homogeneous dtbpy-Ir catalyst highlights the beneficial site isolation effects offered by the MOF support. Lastly, **1-bpymc-Ir-0.1**, containing immobilized 4′-methyl-2,2′-bipyridine-4-carboxylate (bpymc^–^) ligands, was prepared to determine the impact of removing one of the carboxylate tether sites. **1-bpymc-Ir-0.1** exhibited significantly lower catalytic activity than **1-Ir-0.1**, indicating that the dual tethering provided by the bpydc ligands is beneficial for catalyst stabilization (Table 1, entry 9).

**Table 1:**
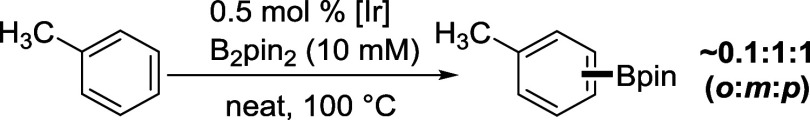
Catalytic C–H Borylation of Toluene^a^

entry	catalyst	time (h)	TON^b^
1	**1-Ir-0.1**	1	124
2	**1-Ir-0.2**	1	159
3	**1-Ir-0.3**	1	221
4	**1-Ir-0.4**	1	320
5	**1-Ir-0.5**	1	355
6	**1-Ir-0.5** (0.12 mol %)	20	1560
7	[Ir(OMe)(cod)]_2_ + MFU-4*l-*Cl	20	12
8	[Ir(OMe)(cod)]_2_ + dtbpy	1	252
9	**1-bpymc-Ir-0.1** (1.0 mol %)	20	88

a Reaction conditions: B_2_pin_2_ (10 mM), catalyst (0.0008 mmol Ir), neat, 100 °C.

b Turnover numbers (TON) are reported as total borylated products per Ir site. TONs were determined by GC-FID with respect to an internal standard (hexamethylbenzene).

Reaction profiles for C–H borylation of toluene with B_2_pin_2_ reveal induction periods that vary with the MOF catalyst site density (Figure 3a). Nevertheless, all of the **1-Ir-*x*** MOF catalysts reach 400 TON within 3 h, reflecting complete consumption of B_2_pin_2_ and the pinacolborane (HBpin) byproduct. When HBpin is used as the borylating reagent, the MOF catalysts exhibit nearly linear reaction profiles without any observable induction periods (Figure 3b). This behavior is consistent with previous mechanistic studies of homogeneous catalyst systems that show HBpin is more effective than B_2_pin_2_ at generating catalytically active Ir­(Bpin)_3_ species.^5^ The positive correlation between catalyst site density and activity is also less pronounced with HBpin. All of the catalysts except **1-Ir-0.1** reach completion (200 TON) within 1 h, and **1-Ir-0.4** is slightly more active than the other members of the series. These results are contrary to the trend observed for our previously reported iridium *diphosphine* catalyst immobilized in MFU-4*l*, where increased catalyst site densities resulted in decreased activity owing to diffusion limitations.^33^ In the present case, the smaller steric profile of the bpydc-Ir catalyst species and its rigid immobilization along one edge of the A-type pore should provide for less hindered substrate/reagent diffusion within the framework.

**Figure 3:**
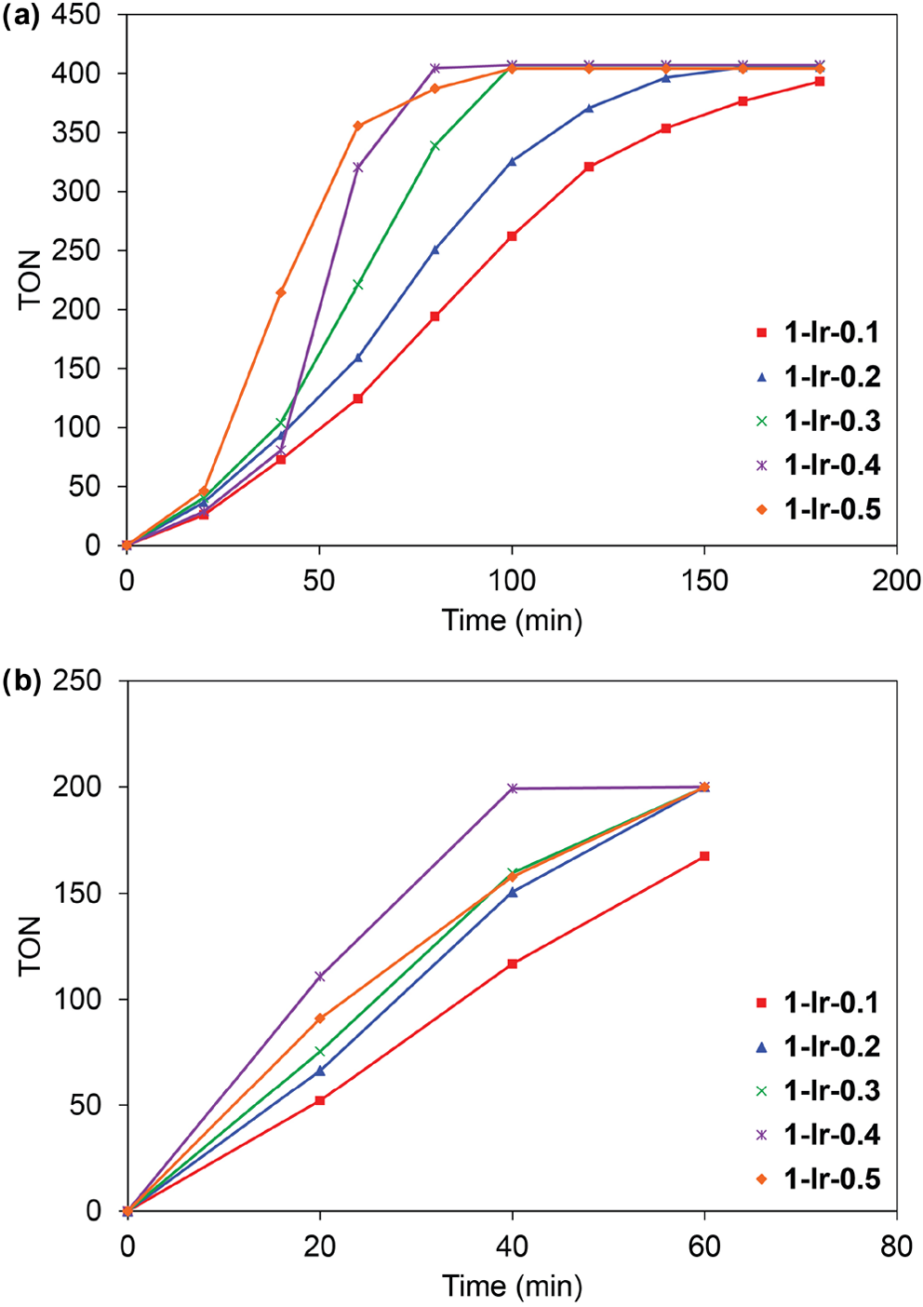
Reaction profiles for C–H borylation of toluene using (a) B_2_pin_2_ and (b) HBpin as borylating reagents. All reactions were performed with 0.5 mol % Ir loading with respect to the borylation reagent.

A hot filtration test shows an abrupt halt in catalytic turnover after removing the solid MOF, indicating that leached iridium species are not responsible for the observed catalysis (Figure S13). At 5 mol % catalyst loading, **1-Ir-0.5** could be recycled for up to 5 runs with no observable loss in turnover (Figure S14). However, attempts to recycle the catalyst at lower loadings (0.5 and 1 mol %) results in significantly decreased activity for subsequent runs (Figures S15–S16). Even with rapid recycling at low conversion (∼30% at 30 min), the MOF only retains 55–65% of its initial activity at 0.5 mol % loading (Figure S17). PXRD analysis and scanning electron microscopy (SEM) images show that MOF crystallinity, particle size, and morphology are retained after the attempted recycling studies (Figures S18–S20). Additionally, ICP-OES analysis of the recovered MOF shows minimal Ir leaching (Table S1). These results support the heterogeneous nature of the MOF catalyst and suggest its poor recyclability is not caused by Ir leaching or framework degradation. We surmise that the catalytically active iridium species undergo deactivating side reactions during the recycling process. In support of this notion, **1-Ir-0.5** provided 1560 TONs after 20 h at 0.12 mol % iridium loading (Table 1, entry 6), indicating that catalyst activity is maintained over long periods under the reaction conditions. This is also among the highest level of activity reported for supported iridium bipyridine catalysts (Table S2).^28,30,31^

Toluene C–H borylation with **1-Ir-0.5** at 1 mol % Ir loading was screened in different solvents (1,4-dioxane, cyclohexane, and heptane) using HBpin as the borylating reagent (Table S3). Catalyst activity increases with increasing initial arene concentration, [Arene]_0_, in all of the solvents tested. This behavior is consistent with arene C–H bond activation as the rate-limiting step, which has been proposed for homogeneous iridium catalysts.^5,39^ **1-Ir-0.5** gave similar TONs (∼77) in heptane, cyclohexane, and 1,4-dioxane after 20 h with [Arene]_0_ = 1–2 M. However, the catalyst performed best in heptane at lower arene concentrations, providing 31 TON for [Arene]_0_ = 500 mM. Based on these results, heptane was used as a solvent for subsequent substrate screening studies.

Next, we sought to explore the influence of the MOF microenvironment on product regioselectivity using monosubstituted arene substrates (Table 2). **1-Ir-0.5** shows a modest increase in *meta*:*para* (*m*:*p*) product selectivity for C–H borylation of *tert*-butyl benzene (**3**, 5.6:1) in comparison to the near statistical distribution observed for toluene (**2**). However, with a larger phenyl­(triisopropyl)­silane substrate (**4**), the MOF catalyst exhibits dramatically increased *m*:*p* distribution of 24:1. In comparison, the homogeneous dtbpy-Ir catalyst provides only slightly increased selectivity of *m*:*p* = 3.8:1. Both catalysts exhibit similar modest activity under the solvent conditions (13–15 TON, 38–43% yield).

**Table 2:**
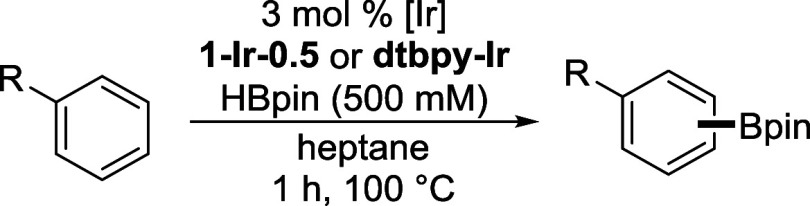

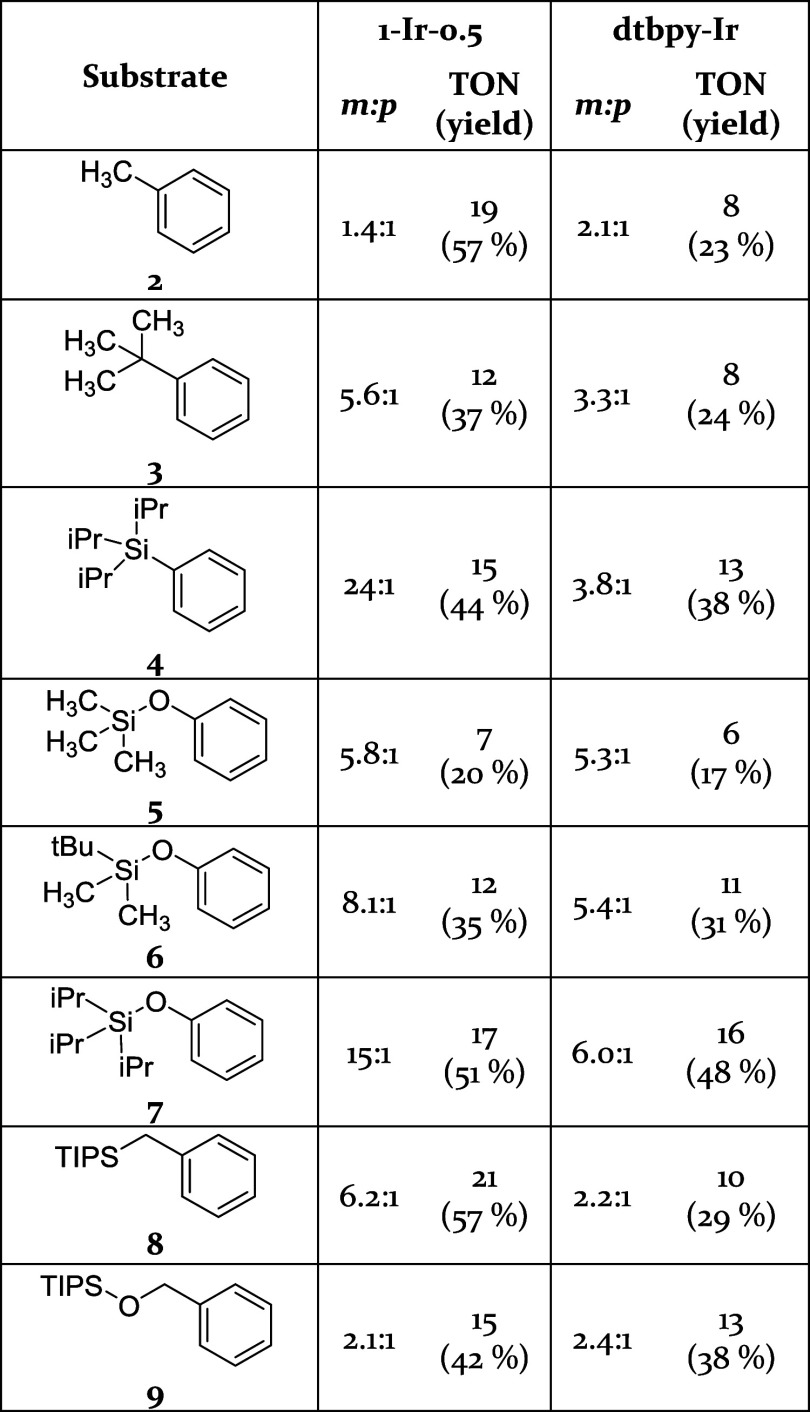
Substrate Scope for C–H Borylation with **1-Ir-0.5** and dtbpy-Ir^a^

a Reaction conditions: Arene (500 mM), HBpin (500 mM), catalyst (0.0008 mmol Ir), 100 °C, 1 h. Turnover numbers (TON) are reported as total borylated products per Ir site. TONs and *meta:para* selectivity were determined by GC-FID with respect to an internal standard (hexamethylbenzene or 1,3,5-trimethoxybenzene).

Given this result, we investigated a series of phenoxysilane substrates (**5–7**). **1-Ir-0.5** shows increasing *m*:*p* selectivity with increasing silane substituent size. For the largest member of the series, **7**, the MOF provides a 15:1 *m*:*p* product distribution while dtbpy-Ir produces only a 6.0:1 product ratio. The latter result is consistent with previous reports.^40^ Upon lowering the reaction temperature to 60 °C, **1-Ir-0.5** experienced a notable increase in *meta* selectivity for **7** (*m*:*p* = 20:1) but at the expense of product yield (7 TON, 21%). Replacing the phenoxy group of **7** with an isosteric benzyl group in **8** results in a decrease in selectivity for both **1-Ir-0.5** (*m*:*p* = 6.2:1) and dtbpy-Ir (*m*:*p* = 2.2:1), but the MOF maintains a greater bias for formation of the *meta* product. Extending the steric influence of the TIPS group away from the arene with a benzyloxy group (**9**) results in a complete loss of selectivity, yielding nearly statistical product distributions for both **1-Ir-0.5** (*m*:*p* ≈ 2.1:1) and dtbpy-Ir (*m*:*p* ≈ 2.4:1).

Considering the good selectivity for *meta* C–H borylation of PhOTIPS (**7**), we expanded the substrate scope to a series of *ortho*-functionalized substrate derivatives (Table 3, substrates **10–15**). The introduction of electron donor groups (**10–12**) resulted in both decreased *a:b* isomer selectivity and catalyst activity for **1-Ir-0.5**. However, the MOF catalyst maintains a greater bias toward forming the *a* isomer than dtbpy-Ir. The presence of electron withdrawing trifluoromethyl (**13**) and halide (**14–15**) groups at the *ortho* position results in a loss of selectivity with respect to the homogeneous catalyst, suggesting that substrate electronic effects may compete with the steric influence of the MOF pore environment.

**Table 3:**
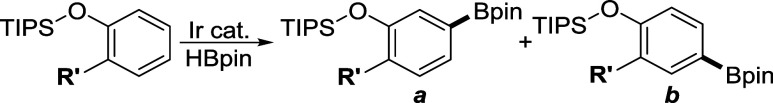
C–H Borylation of Ortho Disubstituted Substrates with **1-Ir-0.5** and dtbpy-Ir^a^

		**1-Ir-0.5**		dtbpy-Ir	
sub	R′	*a*:*b*	TON (yield)	*a*:*b*	TON (yield)
10	CH_3_	8.8:1	9 (28%)	3.0:1	10 (33%)
11	iPr	2.7:1	6 (23%)	1.5:1	13 (39%)
12	OCH_3_	2.5:1	10 (31%)	1.1:1	13 (41%)
13	CF_3_	1:1.4	22 (70%)	2.8:1	22 (69%)
14	Cl	2.0:1	19 (62%)	1.9:1	21 (65%)
15	Br	1.2:1	19 (58%)	1.8:1	20 (61%)

a Reaction conditions: Arene (500 mM), HBpin (500 mM), catalyst (0.0008 mmol Ir), 100 °C, 1h. Turnover numbers (TON) are reported as total borylated products per Ir site. TONs and *a:b* isomer selectivity were determined by GC-FID with respect to an internal standard (hexamethylbenzene).

DFT calculations were carried out to better understand the *meta* regioselectivity observed for **1-Ir-0.5**. Since C–H bond activation is expected to be the product-determining step, reaction pathways were calculated for substrate activation at the *meta* and *para* positions of PhTIPS (**4**). Geometry optimizations were performed in Orca v6.0.1 ^41,42^ using a multiscale approach with a truncated model cage of the MFU-4*l* A-type pore containing the embedded (bpydc)­Ir­(Bpin)_3_ catalyst species. The bpy-Ir­(Bpin)_3_ fragments with bound substrate (high-level region) were treated with the r^2^SCAN-3c composite DFT method.^43^ The remaining cage and link atoms (low-level region) were treated with GFN2-xTB,^44^ and their coordinates were fixed during the optimizations. Subsequent calculations were performed on the optimized high-level region (bpyIr­(Bpin)_3_ + substrate) without the MOF cage (see Supporting Information for full details.) Final electronic single point energies were computed at the ωB97M-V/def2-QZVP level^45,46^ with matching auxiliary basis sets,^47^ a large-core ECP on Ir,^48^ and the CPCM­(heptane) implicit solvation model.^49,50^

The free energy reaction profiles show that formation of arene adducts (AA) with the (bpydc)­Ir­(Bpin)_3_ catalyst is exergonic (Figure 4a). Despite slightly different orientations of the PhTIPS substrate within the pore, the starting adducts for C–H activation at the *meta* and *para* sites exhibit similar relative energies (−5.1 kcal/mol for AA_*meta*_ and −4.4 for AA_*para*_). Oxidative addition of the *para* C–H bond is calculated to have a free energy barrier of 23.1 kcal/mol, while activation at the *meta* position has a lower barrier of 19.7 kcal/mol. This difference (3.4 kcal/mol) is in line with the experimentally observed *meta*:*para* product selectivity (24:1). Inspection of the transition state (TS) structures (Figure 4b) suggests that TS_*para*_ experiences more strain than TS_*meta*_ from the pore restriction. For TS_*para*_, the large TIPS group extends into the pore window, forcing the arene to bend at the Ir–C_*para*_ bond (∠156°). A similar scenario is observed for the oxidative addition products (OA, Figure S24), correlating with the higher relative free energy of OA_*para*_ (+8.8 kcal/mol) versus OA_*meta*_ (+5.8 kcal/mol).

**Figure 4:**
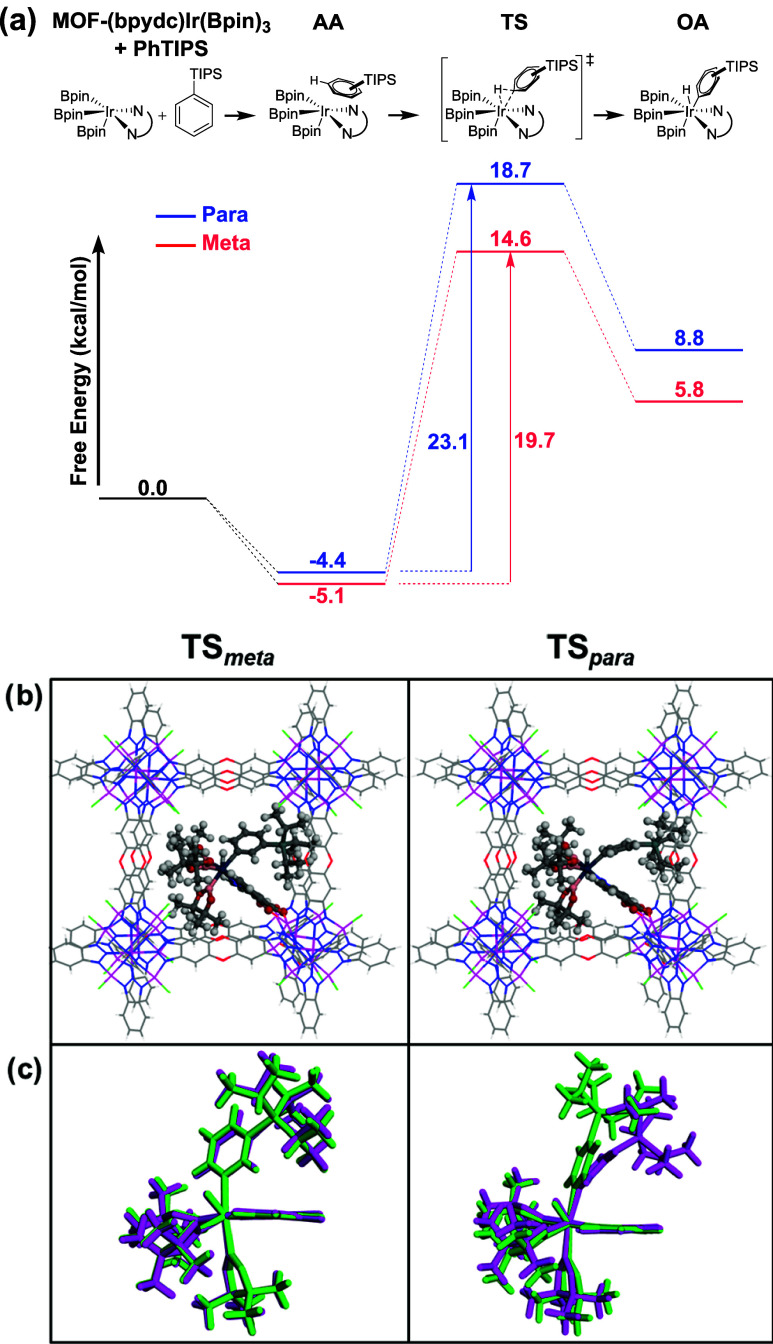
(a) DFT-calculated free energy reaction profiles for *meta* and *para*-C–H activation of PhTIPS in **1-Ir-0.5**. (b) Optimized transition states (r^2^scan-3c:GFN2-xTB) in the truncated MOF cage. (c) Overlay of transition state structures with constraints from the MOF cage (purple) and fully relaxed (green) geometry optimizations.

To corroborate the MOF-induced strain, the AA, TS, and OA structures were allowed to fully relax with geometry optimizations in the absence of the cage. The difference in free energy barriers for *meta* and *para* C–H activation in the relaxed structures is <1 kcal/mol (Figure S27), which correlates with the large decrease in *meta*:*para* selectivity observed for the homogeneous dtbpy-Ir catalyst. Overlays of the MOF-constrained and fully relaxed geometries reveal the different effects of MOF confinement on the *meta* and *para* isomers (Figures 4c and S26). The substrate orientation for TS_*meta*_ is largely unchanged without the influence of the MOF cage. However, the TS_*para*_ structure experiences a notable relaxation of the arene bending at the Ir–C_*para*_ bond (∠171°) compared to the MOF-constrained structure (∠156°).

## Conclusions

A simple postsynthetic modification route is employed to immobilize iridium bipyridine complexes in MFU-4*l*, resulting in highly active precatalysts for C–H borylation of arenes. The MOF with the highest catalyst site density, **1-Ir-0.5**, provides optimal activity for C–H borylation of toluene with B_2_pin_2_ and yielded up to 1560 TONs at 0.12 mol % Ir loading. This is among highest level of activity reported for supported iridium bipyridine catalysts. Optimized solvent conditions allowed screening of a library of arene substrates. Remarkably, **1-Ir-0.5** exhibits high *meta*:*para* product regioselectivity (up to 24:1) for substrates bearing large triisopropylsilyl (TIPS) groups. An analogous homogeneous catalyst, dtbpy-Ir, exhibits only modest selectivity (∼4:1), indicating product bias results from the sterically confined MOF pore environment. Consistent with the experimental observations, DFT calculations for the MOF-embedded catalyst show that the free energy barrier for C–H activation at the *para* position of PhTIPS is ∼3.4 kcal/mol higher than for the *meta* site. They further reveal that the restricted MOF pore environment induces strain in the transition state and oxidative addition product formed for the *para* isomer. Future studies will focus on leveraging the tunability of the MOF pore microenvironment to expand substrate scope and influence product selectivity in other catalytic organic transformations.

## Experimental Section

### General Considerations

All manipulations were carried out using a nitrogen-filled glovebox unless otherwise noted. Toluene, cyclohexane, 1,4-dioxane, tetrahydrofuran, and acetonitrile were degassed by sparging with ultrahigh purity argon and passed through columns of drying agents using a Pure Process Technologies (PPT) solvent purification system. Heptane (Acros Organics) was dried over calcium hydride, vacuum distilled, and stored over 4 Å sieves in an N_2_-filled glovebox. H_2_btdd,^51^ MFU-4*l*,^36^ 4′-methyl­(2,2′-bipyridine)-4-carboxylic acid (Hbpymc),^52^ and [Ir­(OMe)­(cod)]_2_ ^53^ were prepared according to literature procedures. Bis­(pinacolato)­diboron (Frontier Scientific) and pinacolborane (TCI America) were used as received and stored at −25 °C in an N_2_-filled glovebox. Hexamethylbenzene (TCI America) and 1,3,5-trimethoxybenzene (ThermoSci) were dried prior to use and stored in an N_2_-filled glovebox. All arene substrates were either dried over calcium hydride and distilled or degassed via freeze–pump–thaw methods and stored in an N_2_-filled glovebox over 4 Å sieves. Lithium hydroxide (ThermoSci), 2,2′-bipyridine-4,4′-dicarboxylic acid (Ambeed), and methanol (Fisher) were used as received. Phenoxysilane substrates **5–7** (PhOSiR_3_, SiR_3_ = TMS, TBDMS, TIPS) were prepared following literature procedures.^54,55^ Other arene substrates (**4**, **8**-**15**) were synthesized using the procedures described below and purified by column chromatography using a Teledyne CombiFlash NextGen 300 Flash Chromatography System.

Inductively coupled plasma optical emission spectroscopy (ICP-OES) was performed using an Agilent 5100 ICP-OES. MOF samples were digested using a 3:1 (v/v) mixture of H_2_SO_4_ (ThermoSci, 99.999% metal-basis) and H_2_O_2_ (Fisher). Calibration curves were generated using commercial ICP standards (Zn, SPEX CertiPrep; Ir, VWR Chemicals). Powder X-ray diffraction patterns were measured using a Rigaku Miniflex 600 Diffractometer with nickel-filtered Cu Kα radiation (λ = 1.5418 Å).

MOF samples were acid-digested for solution-state NMR analysis by combining ∼5 mg of sample with 0.75 mL trifluoroacetic acid (Sigma-Aldrich). Deuterated dimethyl sulfoxide (DMSO-*d*_*6*_, 0.25 mL) was added to the suspension to help dissolve the organic components as well as provide a lock signal for shimming. Solution-state NMR spectra were obtained using a Bruker Neo 400 MHz NMR spectrometer equipped with a BBFO broadband probe. Solvent suppression was conducted with 180° water-selective excitation sculpting with a 1 ms pulse sequence. The transmittance frequency was centered on the solvent resonance selected for suppression. For ^1^H NMR spectra, the solvent residual resonance was used as an internal reference.

Single-component gas adsorption isotherms were measured using a Micromeritics 3Flex Surface Characterization Analyzer. Gas adsorption measurements were performed using ultrahigh-purity (≥99.99%) gases purchased from Praxair (N_2_, NI 5.0UHK; CO, 4.0RS-AS). Prior to analysis, samples were prepared in oven-dried sample tubes equipped with TranSeals (Micromeritics) then activated and degassed at 45 °C (1 °C min^–1^) under vacuum until the outgas rate was less than 0.0033 mbar min^–1^. BET surface areas were calculated from the N_2_ adsorption isotherms (77 K) by fitting the appropriate pressure range to the BET eq (0.0001 < *P*/*P*_o_ < 0.09) determined by the consistency criteria of Rouquerol.^56,57^ GC-FID data were collected on an Agilent 7890A GC System with an FID detector. Turnovers were calculated with respect to an internal standard using response factors determined from isolated substrates and products (Tables S4–S5). SEM images were collected using a ThermoFisher Axia scanning electron microscope equipped with an Everhart–Thornley (ET) detector. Samples were prepared by suspending particles in methanol and drop casting onto a silicon chip. Particles were imaged at 15 kV/23 pA-0.4 nA by a tetrode-boosted thermionic source. All DFT calculations were performed using Orca v6.0.1.^41,42^ Full details can be found in the main text and Supporting Information.

### Synthesis of Lithium 2,2′-Bipyridine-4,4′-dicarboxylate

In a 100 mL round-bottom flask, 2,2′-bipyridine-4,4′-dicarboxylic acid (0.600 g, 2.46 mmol) was suspended in methanol (40 mL). A solution of LiOH (0.210 g, 5.0 mmol) in methanol (20 mL) was added dropwise via an addition funnel over 1 h. The resulting mixture was stirred for an additional 12 h, resulting in a light-yellow solution with a small amount of white precipitate. The solid was removed by filtration through Celite. The solvent was then evaporated *in vacuo* to afford an off-white solid. The isolated compound was dried under high vacuum overnight. Yield: 0.593 g (94%). ^1^H NMR (400 MHz, DMSO-*d*_6_): δ 8.73 (s, 2H), δ 8.58 (d, 2H, ^3^ *J*_H–H_ = 4 Hz), δ 7.69 (d, 2H, ^3^ *J*_H–H_ = 4 Hz). Lithium 4′-methyl-2,2′-bipyridine-4-carboxylate (Li­[bpymc]) was synthesized following a similar procedure as above (Figure S6).

### Synthesis of **1-bpy-*x***

In a 50 mL round-bottom flask, MFU-4*l* (0.100 g, 0.08 mmol) was suspended in methanol (5 mL). A methanol solution of lithium 2,2′-bipyridine-4,4′-dicarboxylate (8 mM, 0.008–0.040 mmol for *x* = 0.1–0.5) was added to the MOF suspension dropwise. The resulting mixture was stirred at 300 rpm for 20 h, and the solid was isolated by vacuum filtration. The resulting MOFs were washed with methanol (5 × 20 mL) before being dried *in vacuo*. The bipyridine functionalized MOFs were activated by heating at 60 °C (1 °C min ^–1^) under high vacuum (10^–5^ bar) for 12 h prior to N_2_ gas adsorption analysis. Bipyridine incorporation was determined by quantitative ^1^H NMR spectroscopy of acid-digested samples (Figures S1–S5). Reference spectrum for **1-bpy-0.5**: ^1^H NMR (400 MHz, TFA/DMSO-*d*_6_): δ 8.81 (s, 1H), δ 8.74 (d, 1H, ^3^ *J*_H–H_ = 4 Hz), δ 8.09 (d, 1H, ^3^ *J*_H–H_ = 4 Hz), δ 7.37 (s, 12H). Empirical formula: Zn_5_Cl_3_(btdd)_3_(bpydc)_0.5_. **1-bpymc-0.1** (Zn_5_Cl_3.9_(btdd)_3_(bpymc)_0.1_) was synthesized following a similar procedure as above. The acid-digested ^1^H NMR spectrum is shown in Figure S7.

### Synthesis of **1-Ir-*x***

In an N_2_-filled glovebox, a solution of [Ir­(OMe)­(cod)]_2_ (1 equiv. Ir per bipyridine) in acetonitrile/tetrahydrofuran (3:1 v/v, 5 mM) was added to solid **1-bpy-** ***x*** (40–100 mg) in a 20 mL scintillation vial. The MOF suspension was heated at 60 °C with gentle stirring (100 rpm) for 1 h. After the reaction, the MOF was isolated by centrifugation and washed with acetonitrile (3 × 10 mL) before being dried *in vacuo*. The metalated MOFs were activated by heating at 45 °C (1 °C min^–1^) under high vacuum (10^–5^ bar) for 12 h prior to N_2_ (77 K) and CO (300 K) gas adsorption analysis. Iridium loadings per formula unit were determined by ICP-OES with respect to zinc (Figures S21–S22 and Table S1).

### Benchmark Catalytic C–H Borylation of Toluene

All catalytic reactions were performed in an N_2_-filled glovebox. For a typical reaction, a 10 mM solution of B_2_pin_2_ (0.040 g, 0.157 mmol) and hexamethylbenzene (∼1 equiv with respect to B_2_pin_2_) was prepared in neat toluene substrate (15.7 mL). **1-Ir-** ***x*** catalyst (0.5 mol % Ir with respect to B_2_pin_2_) was combined with the arene solution in a 20 mL vial and heated at 100 °C while stirring at 300 rpm for 3 h. Aliquots of the reaction mixture (50 μL) were obtained over the course of the reaction and diluted with HPLC grade benzene. Samples were prepared by filtering over Celite to remove particulates prior to analysis by GC-FID.

### Catalytic C–H Borylation of Arenes in Solvent

All catalytic reactions were performed in an N_2_-filled glovebox. For a typical reaction, a solution of HBpin (0.021 g, 0.164 mmol, 500 mM), internal standard (hexamethylbenzene or 1,3,5-trimethoxybenzene, 0.125 equiv with respect to arene), and arene (0.164 mmol, 500 mM) were prepared in solvent. **1-Ir-0.5** catalyst (3 mol % Ir with respect to arene) was combined with the reaction mixture in a 1-dram vial and heated at 100 °C with gentle stirring (100 rpm). Aliquots of the reaction mixture (10 μL) were collected and diluted with HPLC grade benzene. Samples were prepared by filtering over Celite to remove particulates prior to analysis by GC-FID. GC-FID chromatograms can be found in Figures S40–S53. Reactions with the homogeneous dtbpy-Ir catalyst were performed under similar conditions. In place of the MOF catalyst, a THF solution of dtbpy and [Ir­(OMe)­(cod)]_2_ (2:1 ratio, 45 mM Ir) was added to the reaction mixture.

### Recyclability Studies

In a 20 mL scintillation vial, **1-Ir-0.5** was suspended in toluene (6.5–16.5 mL) containing B_2_pin_2_ (10 mM or 40 mM) and a known amount of hexamethylbenzene as an internal standard (amounts of B_2_pin_2_ given below). The reaction was gently stirred at 100 °C for 30 min (0.5 mol %) or 80 min (0.5, 1, and 5 mol %). The supernatant was then analyzed by GC-FID to determine total TON. The supernatant was decanted, and the MOF was resubjected to a fresh reaction solution for a second catalytic run following the procedures above. This process was repeated for 3–5 runs.

Five mol % loading: **1-Ir-0.5** (0.010 g, 0.0033 mmol Ir), toluene (6.5 mL), and B_2_pin_2_ (0.0168 g, 0.067 mmol). The results are shown in Figure S14.

One mol % loading: **1-Ir-0.5** (0.005 g, 0.0017 mmol Ir), toluene (16.5 mL), B_2_pin_2_ (0.0427 g, 0.168 mmol). The results are shown in Figure S15. After 5 runs, the MOF was separated from the reaction mixture via centrifugation and analyzed by PXRD (Figure S18) and SEM (Figures S19–S20).

0.5 mol % loading after 80 min (40 mM B_2_pin_2_): **1-Ir-0.5** (0.0100 g, 0.0033 mmol Ir), toluene (16.7 mL), and B_2_pin_2_ (0.1708 g, 0.672 mmol). The results are shown in Figure S16.

0.5 mol % loading after 30 min (10 mM B_2_pin_2_): **1-Ir-0.5** (0.0028 g, 0.0009 mmol Ir), toluene (15.7 mL), and B_2_pin_2_ (0.0381 g, 0.15 mmol). The results are shown in Figure S17.

### Hot Filtration Test

In two 20 mL scintillation vials, **1-Ir-0.5** (0.0022 g, 0.0007 mmol Ir) was suspended in toluene (14.5 mL) containing B_2_pin_2_ (0.037 g, 0.146 mmol) and a known amount of hexamethylbenzene as an internal standard. After heating at 100 °C for 60 min, the MOF catalyst was removed from one of the reaction vials by quickly filtering the reaction mixture through a 0.45 μm PTFE syringe filter. The filtered solution was transferred to a new 20 mL scintillation vial, sealed, and heated at 100 °C. The reactions were periodically monitored by GC-FID for 120 min. No additional catalytic turnover was observed after removing the MOF catalyst while the control reactions showed continued turnover (Figure S13).

### Control Reaction with MFU-4*l* and [Ir­(OMe)­(cod)]_2_

MFU-4*l* (0.078 g, 0.006 mmol) was suspended in toluene (1 mL) in a 20 mL scintillation vial. A 0.36 mM stock solution of [Ir­(OMe)­(cod)]_2_ was prepared in toluene (0.0045 g/20 mL, 0.0068 mmol). A 1 mL aliquot of the [Ir­(OMe)­(cod)]_2_ stock solution (0.68 μmol Ir) was added to the MOF suspension. The suspension was stirred for 1 h prior to the addition of B_2_pin_2_ (0.0401 g, 0.158 mmol) and hexamethylbenzene (0.0167 g, 0.103 mmol) as an internal standard. The resulting suspension was then diluted with additional toluene to provide an initial B_2_pin_2_ concentration of 10 mM. The reaction mixture was heated for 20 h at 100 °C. Aliquots of the reaction mixture (50 μL) were collected and diluted with HPLC grade benzene. Samples were prepared by filtering over Celite to remove particulates prior to analysis by GC-FID.

### Synthesis of Phenyl­(triisopropyl)­silane (**4**)

In an oven-dried 25 mL Schlenk flask, a solution of phenyl lithium in dibutyl ether (1.9 M, 9.0 mL) was added to dry degassed THF (9.1 mL) under N_2_ and cooled to −78 °C. Triisopropylsilylchloride (4.04 mL) was added dropwise with stirring. The reaction was left at −78 °C for 1 h and then warmed to room temperature and left to stir for 18 h. The flask was then cooled to 0 °C, and water (10 mL) was added dropwise. The product was extracted with diethyl ether (15 mL), washed with 5% aqueous sodium bicarbonate and brine, and then dried over anhydrous sodium sulfate. The solvent was removed *in vacuo*. The yellow oil was then purified by column chromatography using hexane as the eluent. The solvent was removed to yield a colorless oil. The product was degassed following the freeze–pump–thaw method prior to being stored over 4 Å sieves in an N_2_-filled glovebox. The isolated oil was characterized by ^1^H NMR spectroscopy which matched the data reported in the literature (Figure S28).^58^ Yield 1.68 g (42%).

### Synthesis of Benzyl­(triisopropyl)­silane (**8**)

In an oven-dried 25 mL Schlenk flask, dry degassed toluene (4.7 mL) and THF (4.7 mL) were cooled to −78 °C under N_2_ for 30 min. *n*-butyllithium (2.5 M, 8.0 mmol, 3.20 mL) was added dropwise with stirring. The mixture was then warmed to room temperature and left to stir for 3 h. The flask was cooled to −78 °C and triisopropylsilylchloride (1.89 mL) was added dropwise with stirring. The flask was warmed to room temperature and then stirred overnight. The mixture was cooled to 0 °C, and water was added dropwise. The product was extracted with diethyl ether (15 mL), washed with 5% aqueous sodium bicarbonate and brine, and then dried anhydrous sodium sulfate. The solvent was then removed *in vacuo*. The yellow oil was then purified by column chromatography using hexane as the eluent. The solvent was removed to yield a colorless oil. The product was degassed following the freeze–pump–thaw method prior to being stored over 4 Å sieves in an N_2_-filled glovebox. The isolated oil was characterized by ^1^H NMR spectroscopy which matched the data reported in the literature (Figure S30).^59^ Yield 1.40 g (70%).

### Synthesis of Benzyl­(triisopropyl)­silyl Ether (**9**)

In a 100 mL silanized round-bottom flask, benzyl alcohol (1.2 mL, 12 mmol) and imidazole (1.60 g, 24 mmol) were dissolved in anhydrous dichloromethane (60 mL). Triisopropylsilyl chloride was added dropwise via syringe and the reaction was stirred for 9 h. Solvent was then removed *in vacuo* to afford an off-white solid and colorless oil. The oil was extracted with diethyl ether (100 mL) and filtered over Celite. The solvent was then removed *in vacuo* to afford a colorless oil which was purified by column chromatography using hexane as the eluent. The product was degassed following the freeze–pump–thaw method prior to being stored over 4 Å sieves in an N_2_-filled glovebox. The isolated oil was characterized by ^1^H NMR spectroscopy which matched the data reported in the literature (Figure S29).^60^ Yield 2.05 g (62%).

### General Procedure for the Synthesis of Phenoxysilane Arene Substrates **10–15**

In a 100 mL silanized round-bottom flask, the phenol precursor (10.6 mmol) was dissolved in anhydrous dichloromethane (50 mL). Triisopropylchlorosilane (13.8 mmol) and DBU (21.2 mmol) were added dropwise via syringe. The reaction was stirred for an additional 12 h resulting in a light-yellow solution. Solvent was then removed *in vacuo* to afford an off-white solid and colorless oil. The oil was extracted into diethyl ether (100 mL) and filtered over Celite. The solvent was then removed *in vacuo* to afford a colorless oil which was purified by column chromatography using hexane as the eluent. Samples were degassed following the freeze–pump–thaw method prior to being stored over 4 Å sieves in an N_2_-filled glovebox. The isolated products were characterized by ^1^H NMR spectroscopy (Figures S34–S39).

## Supplementary Material





## Notes

DFT-optimized structure data (ZIP)
NMR spectral data, analytical data, gas adsorption isotherms and data, PXRD data, DFT computational details GC-FID chromatograms, additional experimental details (PDF)
