# Supplementary material for: Regioselective Arene C–H Borylation with a MOF-Immobilized Iridium Bipyridine Catalyst
Source: ACS Appl Mater Interfaces. 2026 Feb 13;18(7):11467–76. doi: 10.1021/acsami.5c24764 (PMC12954667; doi:10.1021/acsami.5c24764)
Supplement: Supplementary file 2 [file am5c24764_si_002.pdf]

Supporting Information

Regioselective Arene C–H Borylation with a MOF-Immobilized Iridium Bipyridine Catalyst

Jordon S. Hilliard,[#] May M. Cheline,[#] Andrew J. Robinson, and Casey R. Wade*

Department of Chemistry and Biochemistry, The Ohio State University, Columbus, Ohio 43210, United States

*E-mail: wade.521@osu.edu

Contents

Figure S1. ^{1}H NMR (400 MHz, DMSO-d_6) spectrum of acid-digested **1-bpy-0.1**. 5

Figure S2. ^{1}H NMR (400 MHz, DMSO-d_6) spectrum of acid-digested **1-bpy-0.2**. 6

Figure S3. ^{1}H NMR (400 MHz, DMSO-d_6) spectrum of acid-digested **1-bpy-0.3**. 7

Figure S4. ^{1}H NMR (400 MHz, DMSO-d_6) spectrum of acid-digested **1-bpy-0.4**. 8

Figure S5. ^{1}H NMR (400 MHz, DMSO-d_6) spectrum of acid-digested **1-bpy-0.5**. 9

Figure S6. ^{1}H NMR (400 MHz, MeOD-d_4) spectrum of Li[bpymc]. 10

Figure S7. ^{1}H NMR (400 MHz, DMSO-d_6) spectrum of acid-digested **1-bpymc-0.1**. 11

Figure S8. Powder X-ray diffraction patterns of **1-bpy-*x*** and MFU-4*l*. 12

Figure S9. N_2 gas adsorption isotherms (77 K) and calculated BET surface areas for **1-bpy-*x***. 13

Figure S10. DFT Pore size distributions (2D-NLDFT, Carbon Slit, N_2, 77K) of **1-Ir-*x***. 14

Figure S11. CO gas adsorption isotherms (300 K) of **1-Ir-*x***. 15

Figure S12. ATR-IR spectrum of **1-Ir-0.3** and **1-Ir-0.3** after CO gas adsorption analysis. 16

Figure S13. Hot filtration test with **1-Ir-0.5**. 17

Figure S14. Recycling experiment (80 min) using 5 mol % **1-Ir-0.5** for the C–H borylation of toluene. 18

Figure S15. Recycling experiment (80 min) using 1 mol % **1-Ir-0.5** for the C–H borylation of toluene. 19

Figure S16. Recycling experiment (80 min) using 0.5 mol % **1-Ir-0.5** for the C–H borylation of toluene. 20

Figure S17. Recycling experiment (30 min) using 0.5 mol % **1-Ir-0.5** for the C–H borylation of toluene. 21

Figure S18. Powder X-ray diffraction patterns of **1-Ir-0.5** before and after catalysis. 22
Figure S19. SEM image of 1-Ir-0.5 prior to catalysis. 23
Figure S20. SEM image of 1-Ir-0.5 after 1 mol % recycling study. 23
Figure S21. Iridium ICP-OES calibration curve. 24
Figure S22. Zinc ICP-OES calibration curve. 24
Table S1. ICP-OES results for **1-Ir-*x***. 25
Table S2. Comparison of MOF-supported Iridium C–H Borylation Catalysts 26
Table S3. C–H Borylation of toluene in solvent. 27
Details of DFT Calculations 28
Figure S23. Structure of OA_{meta} embedded in MOF cage (Top) and extracted structure with link atoms for constrained optimization (Bottom). 29
Figure S24. DFT-optimized structures embedded in the MOF cage. 30
Figure S25. Overlay of AA, TS, and OA structures optimized within the MOF cage (yellow) followed by constrained optimization without the MOF cage (purple). 31
Figure S26. Overlay of DFT-optimized structures of MOF-constrained (purple) and fully relaxed (green) structures. 32
Figure S27. DFT-calculated free energy reaction profiles for *meta* and *para*-C–H activation of PhTIPS with fully relaxed (bpy)Ir(Bpin)$_3$ catalyst and substrate fragments. 33
Figure S28. ^{1}H NMR spectrum of phenyl(triisopropyl)silane (PhTIPS). 34
Figure S29. ^{1}H NMR spectrum of benzyl(triisopropyl)silyl ether (BnOTIPS). 35
Figure S30. ^{1}H NMR spectrum for benzyl(triisopropyl)silane (BnTIPS). 36
Figure S31. ^{1}H NMR spectrum of phenoxy(trimethyl)silane (PhOTMS). 37
Figure S32. ^{1}H NMR spectrum of phenoxy(*tert*-butyldimethyl)silane (PhOTBDMS).. 38
Figure S33. ^{1}H NMR spectrum of phenoxy(triisopropyl)silane (PhOTIPS). 39
Figure S34. ^{1}H NMR spectrum of 2-methyl-phenoxy(triisopropyl)silane (2-Me-PhOTIPS). 40
Figure S35. ^{1}H NMR spectrum of 2-isopropyl-phenoxy(triisopropyl)silane (2-iPr-PhOTIPS). 41
Figure S36. ^{1}H NMR spectrum of 2-methoxy-phenoxy(triisopropyl)silane (2-MeO-PhOTIPS). 42
Figure S37. ^{1}H NMR spectrum of 2-trifluoromethyl-phenoxy(triisopropyl)silane (2-CF_3-PhOTIPS). 43
Figure S38. ^{1}H NMR spectrum of 2-chloro-phenoxy(triisopropyl)silane (2-Cl-PhOTIPS). 44

Figure S39. ^{1}H NMR spectrum of 2-bromo-phenoxy(triisopropyl)silane (2-Br-PhOTIPS). 45

Table S4. Response factor tables for substrates. 47

Table S5. Response factor tables for products. 49

Figure S40. GC-FID chromatogram for the C–H borylation of toluene (500 mM) using **1-Ir-0.5** and HBpin (500 mM) in heptane. 52

Figure S41. GC-FID chromatogram for the C–H borylation of *tert*-butylbenzene (500 mM) using **1-Ir-0.5** and HBpin (500 mM) in heptane. 53

Figure S42. GC-FID chromatogram for the C–H borylation of phenyl(triisopropyl)silane (PhTIPS) (500 mM) using **1-Ir-0.5** and HBpin (500 mM) in heptane. 54

Figure S43. GC-FID chromatogram for the C–H borylation of phenoxy(trimethyl)silane (PhOTMS) (500 mM) using **1-Ir-0.5** and HBpin (500 mM) in heptane. 55

Figure S44. GC-FID chromatogram for the C–H borylation of phenoxy(*tert*-butyldimethyl)silane (PhOTBDMS) (500 mM) using **1-Ir-0.5** and HBpin (500 mM) in heptane. 56

Figure S45. GC-FID chromatogram for the C–H borylation of phenoxy(triisopropyl)silane (PhOTIPS) (500 mM) using **1-Ir-0.5** and HBpin (500 mM) in heptane. 57

Figure S46. GC-FID chromatogram for the C–H borylation of benzyl(triisopropyl)silane (BnTIPS) (500 mM) using **1-Ir-0.5** and HBpin (500 mM) in heptane. 58

Figure S47. GC-FID chromatogram for the C–H borylation of benzyl(triisopropyl)silyl ether (BnOTIPS) (500 mM) using **1-Ir-0.5** and HBpin (500 mM) in heptane. 59

Figure S48. GC-FID chromatogram for the C–H borylation of 2-methyl-phenoxy(triisopropyl)silane (2-Me-PhOTIPS) (500 mM) using **1-Ir-0.5** and HBpin (500 mM) in heptane. 60

Figure S49. GC-FID chromatogram for the C–H borylation of 2-isopropyl-phenoxy(triisopropyl)silane (2-iPr-PhOTIPS) (500 mM) using **1-Ir-0.5** and HBpin (500 mM) in heptane. 61

Figure S50. GC-FID chromatogram for the C–H borylation of 2-methoxy-phenoxy(triisopropyl)silane (2-MeO-PhOTIPS) (500 mM) using **1-Ir-0.5** and HBpin (500 mM) in heptane. 62

Figure S51. GC-FID chromatogram for the C–H borylation of 2-trifluoromethyl-phenoxy(triisopropyl)silane (2-CF_3-PhOTIPS) (500 mM) using **1-Ir-0.5** and HBpin (500 mM) in heptane. 63

Figure S52. GC-FID chromatogram for the C–H borylation of 2-chloro-phenoxy(triisopropyl)silane (2-Cl-PhOTIPS) (500 mM) using **1-Ir-0.5** and HBpin (500 mM) in heptane. 64

Figure S53. GC-FID chromatogram for the C–H borylation of 2-bromo-phenoxy(triisopropyl)silane (2-Br-PhOTIPS) (500 mM) using **1-Ir-0.5** and HBpin (500 mM) in heptane. 65

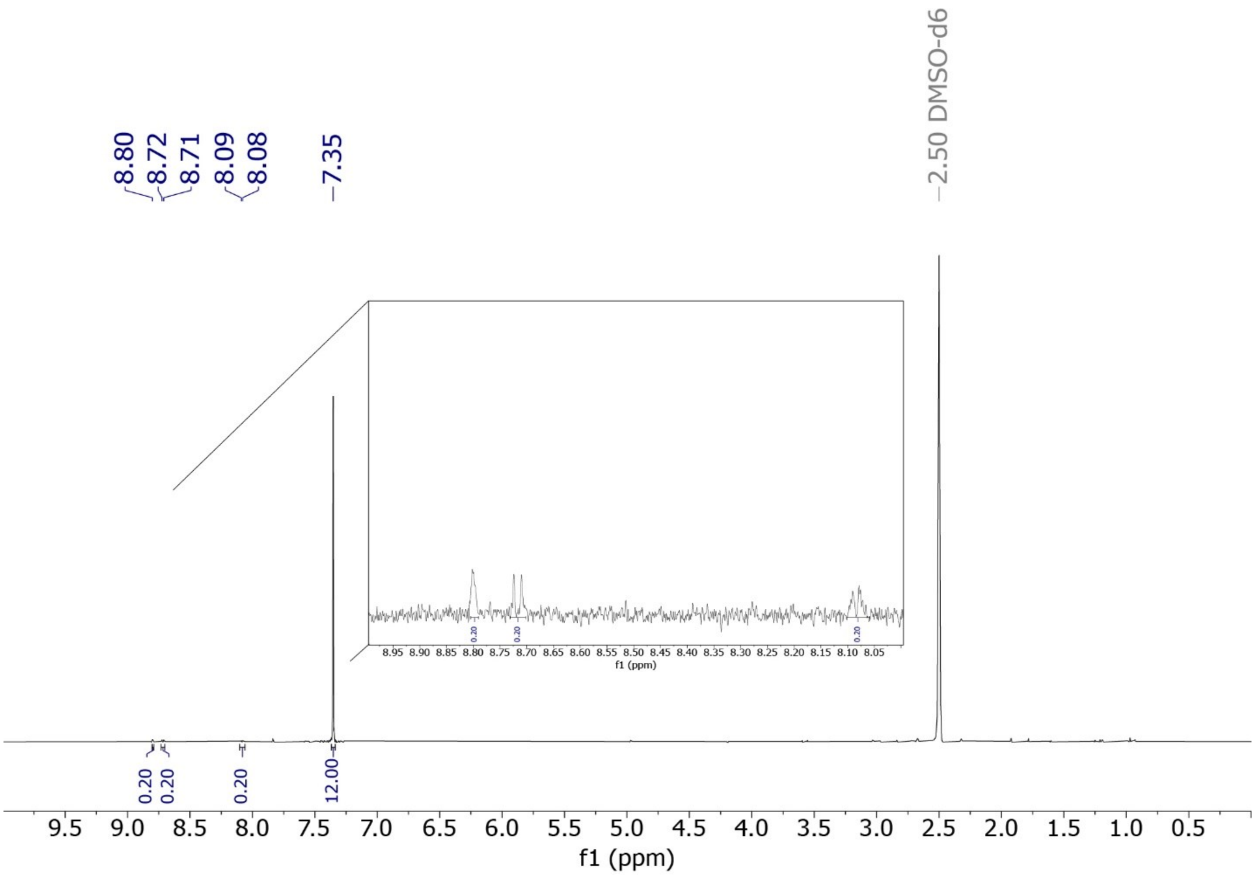

Figure S1. ^{1}H NMR (400 MHz, DMSO-d_6) spectrum of acid-digested **1-bpy-0.1**.

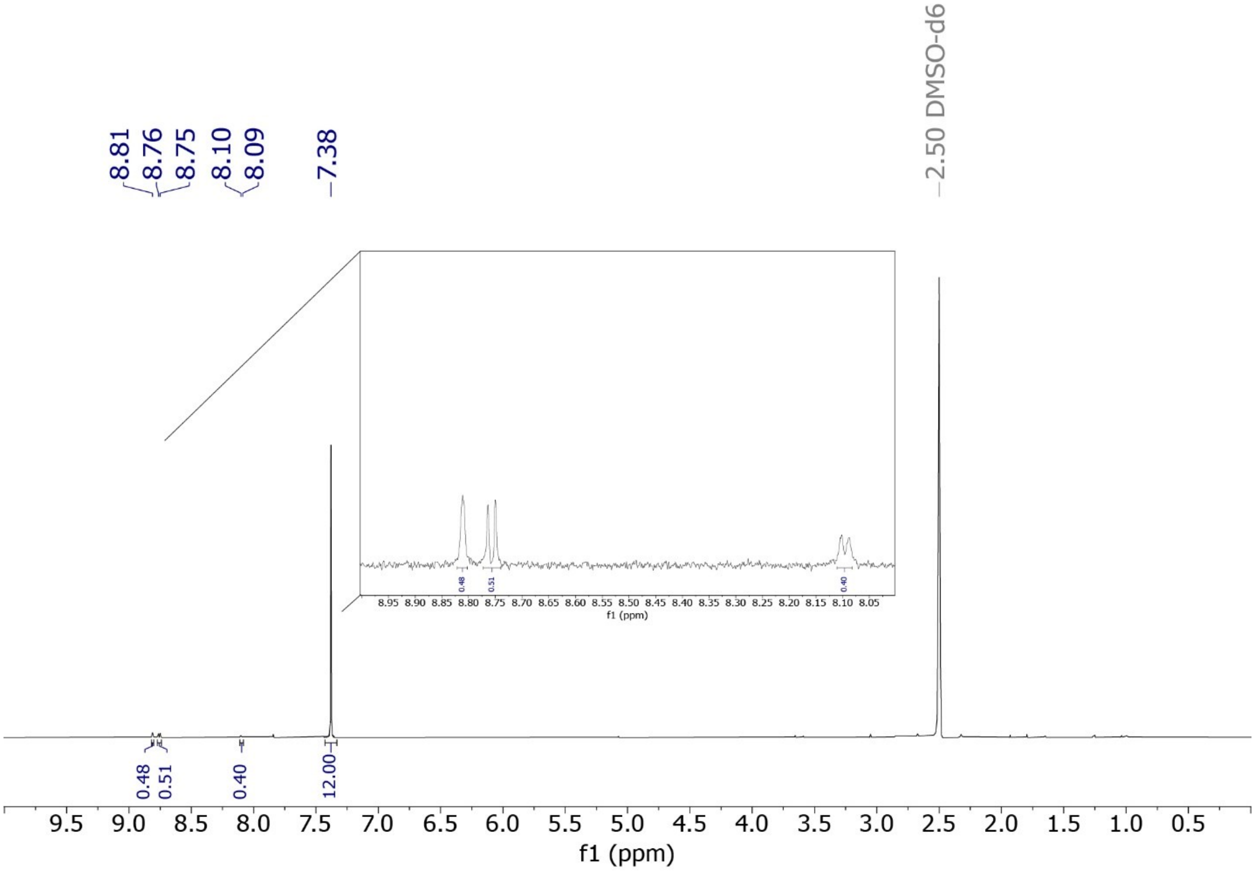

Figure S2. ^{1}H NMR (400 MHz, DMSO-d_6) spectrum of acid-digested **1-bpy-0.2**.

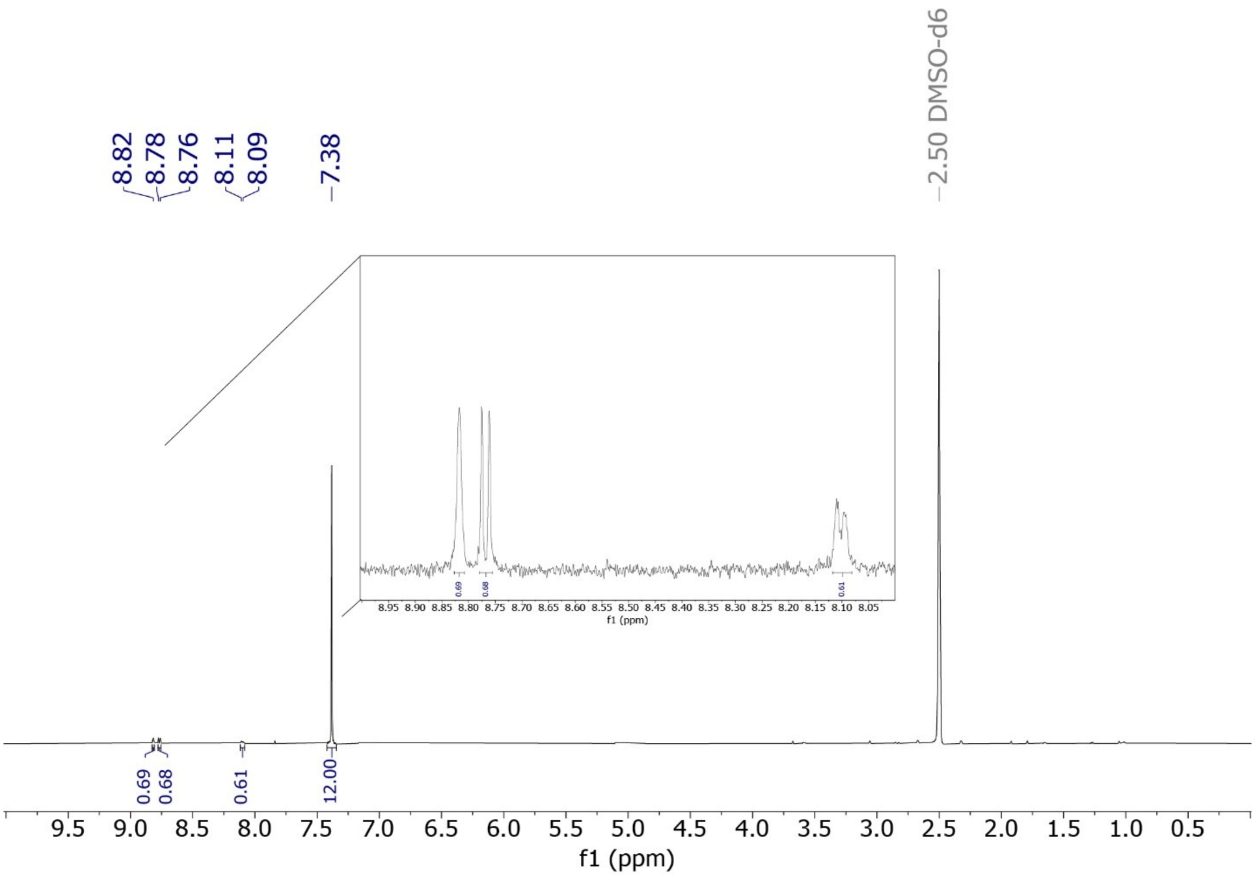

Figure S3. ^{1}H NMR (400 MHz, DMSO-d_6) spectrum of acid-digested **1-bpy-0.3**.

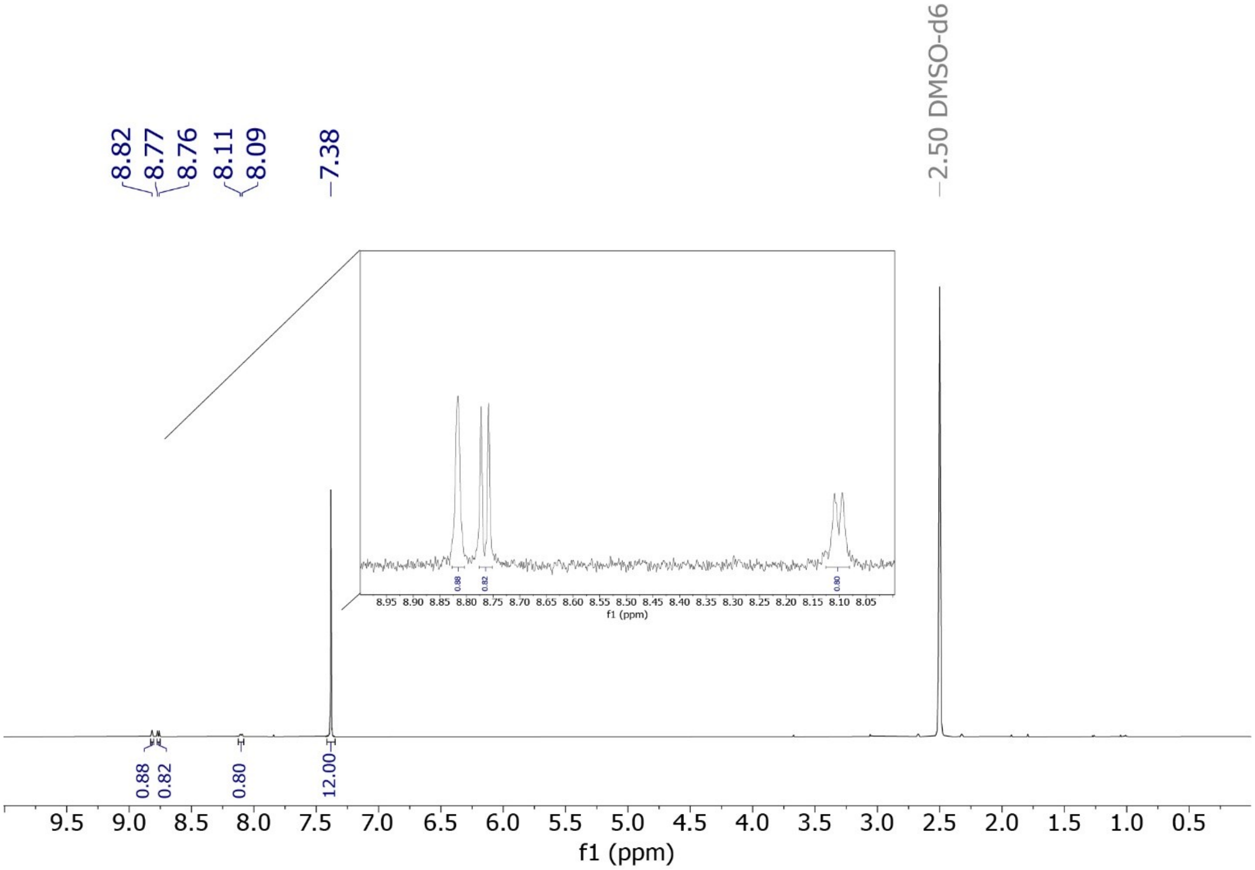

Figure S4. ^{1}H NMR (400 MHz, DMSO-d_6) spectrum of acid-digested **1-bpy-0.4**.

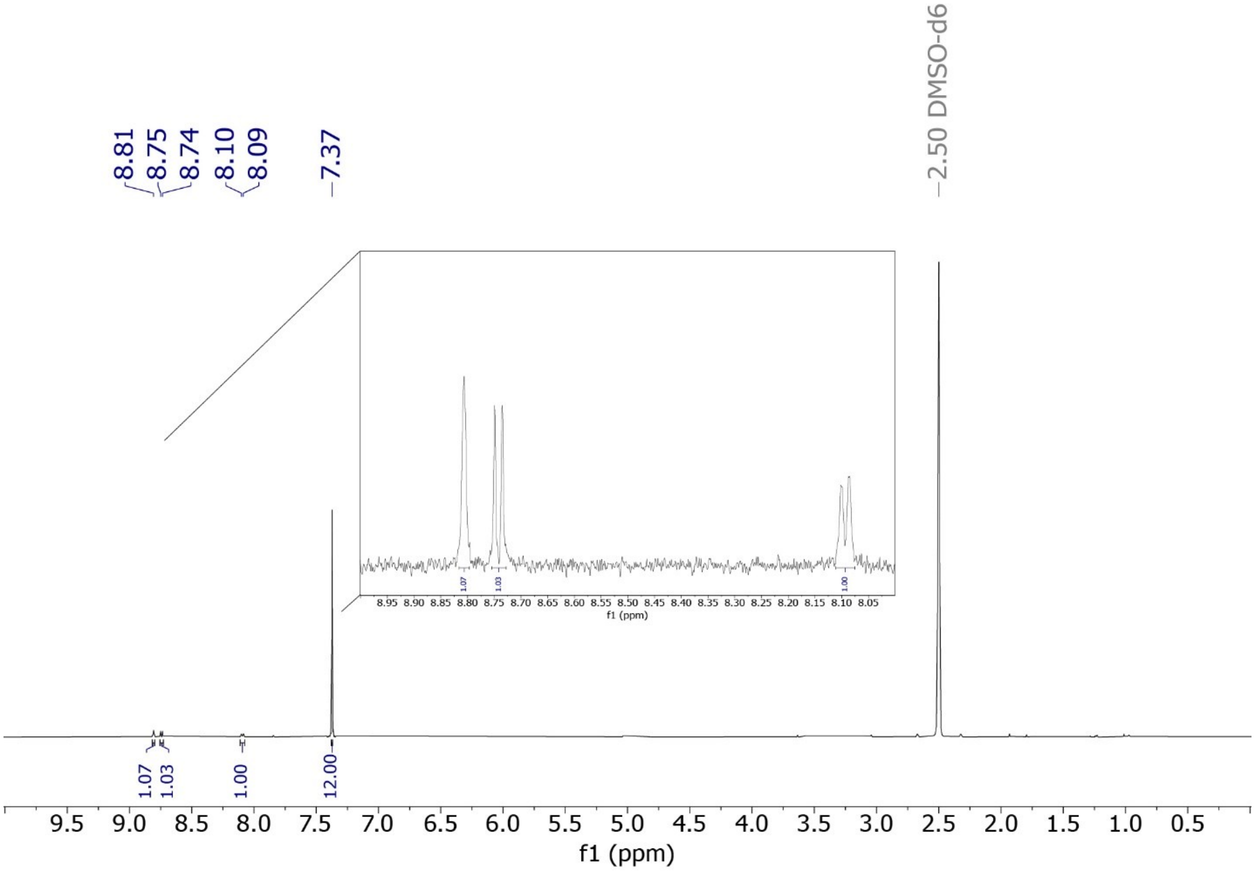

Figure S5. ^{1}H NMR (400 MHz, DMSO-d_6) spectrum of acid-digested **1-bpy-0.5**.

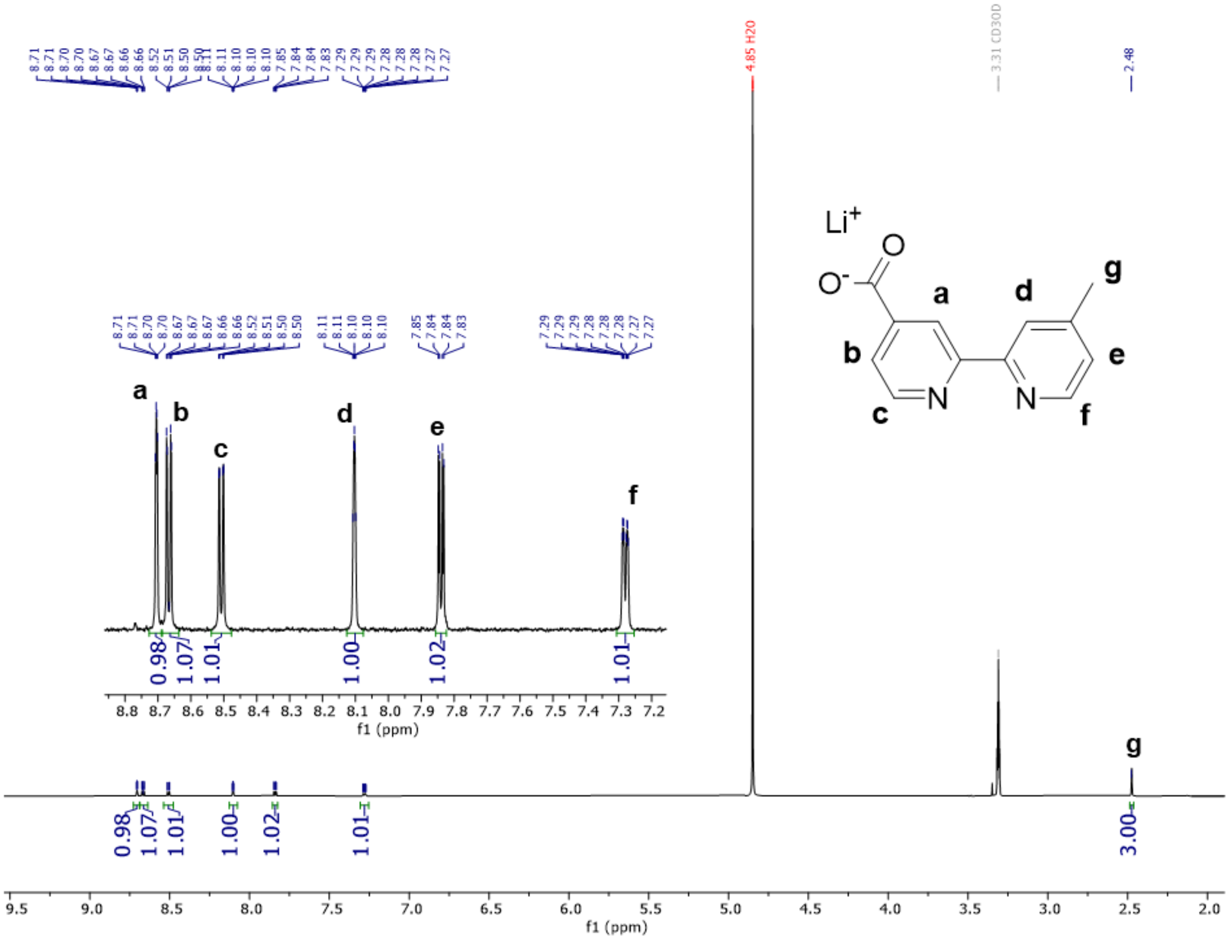

Figure S6. ^{1}H NMR (400 MHz, MeOD-d_4) spectrum of Li[bpymc].

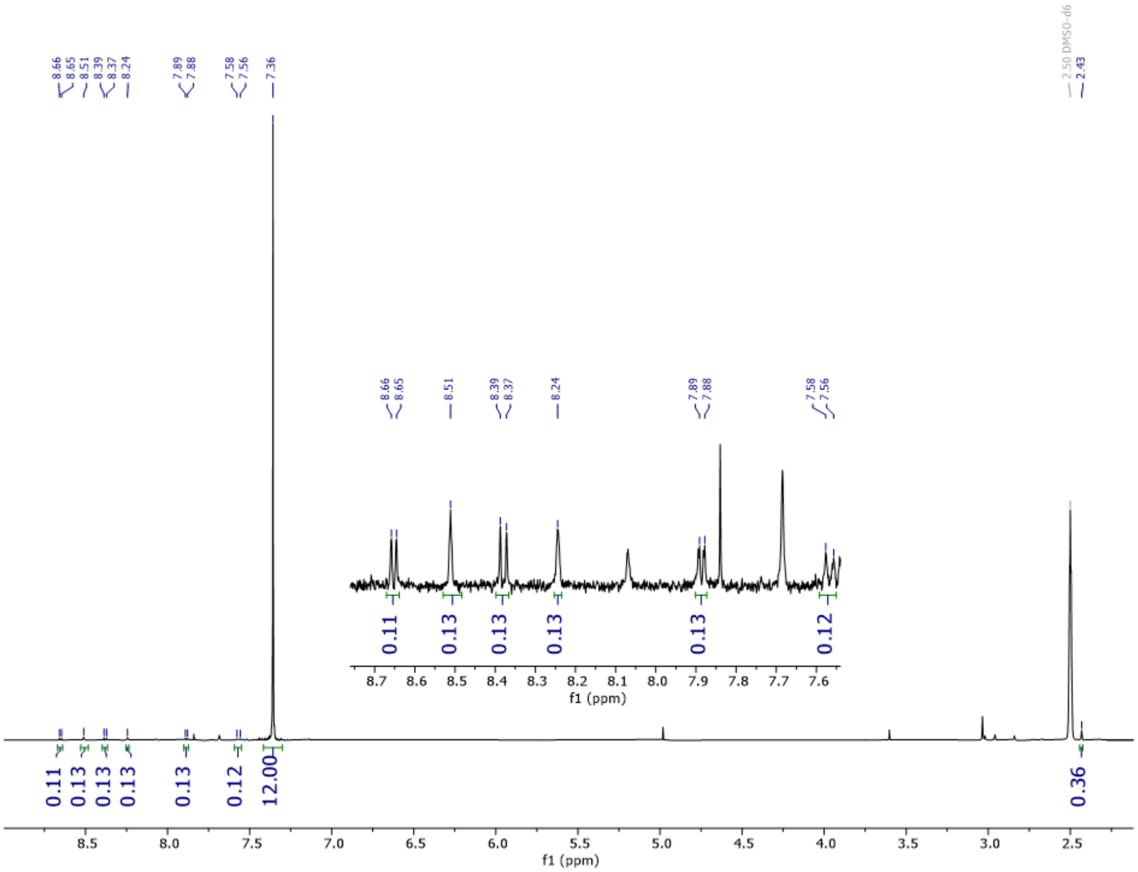

Figure S7. ^{1}H NMR (400 MHz, DMSO-d_6) spectrum of acid-digested **1-bpymc-0.1**.

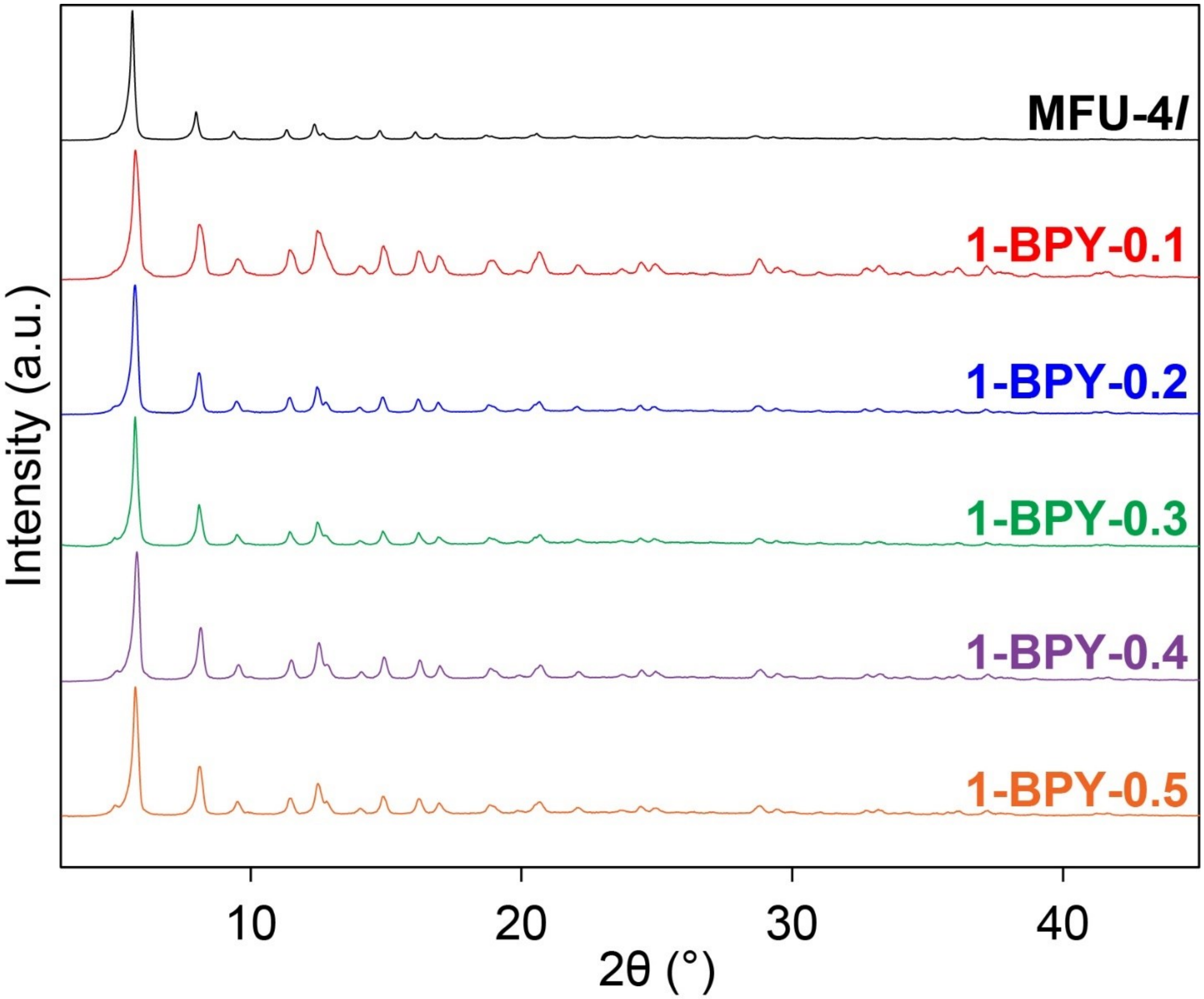

Figure S8. Powder X-ray diffraction patterns of **1-bpy-*x*** and MFU-4*l*.

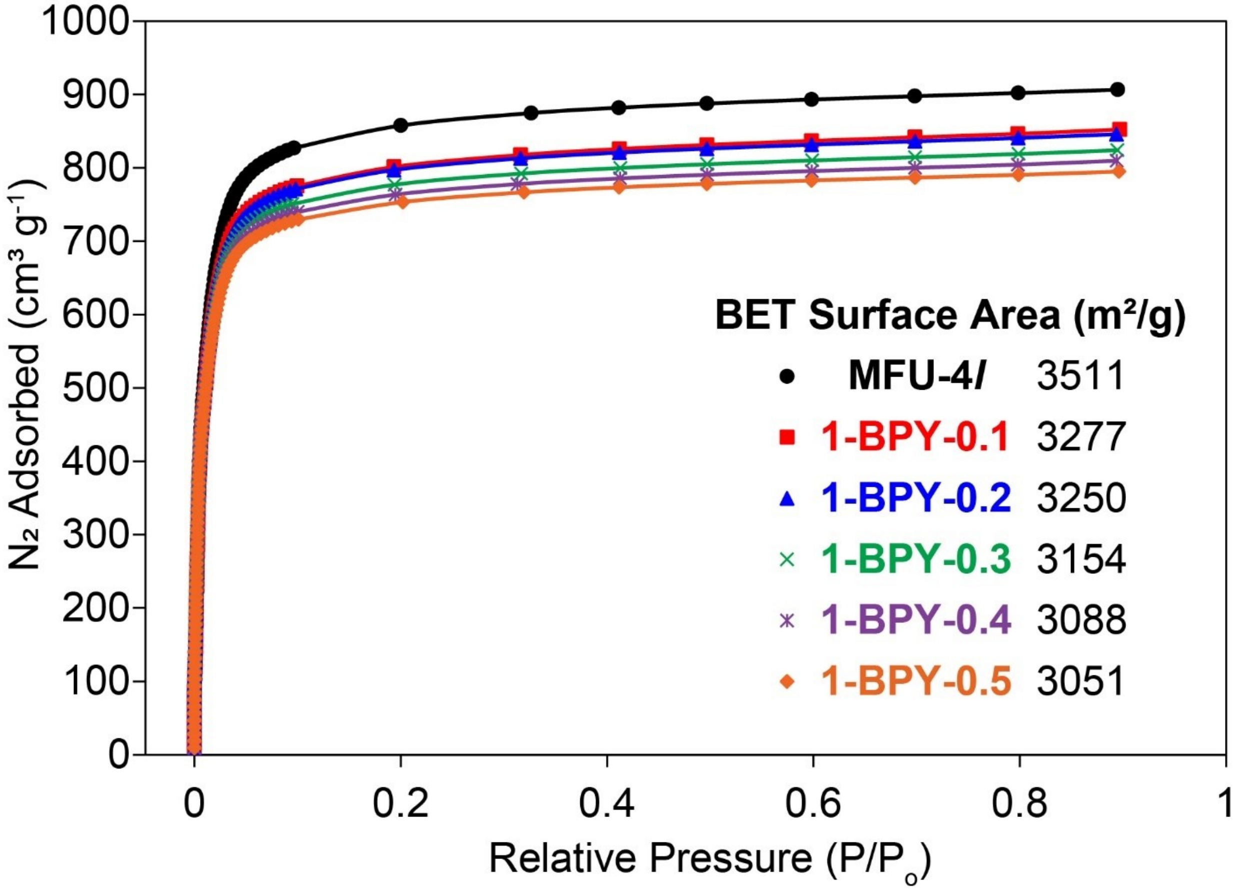

Figure S9. N_2 gas adsorption isotherms (77 K) and calculated BET surface areas for **1-bpy-*x***.

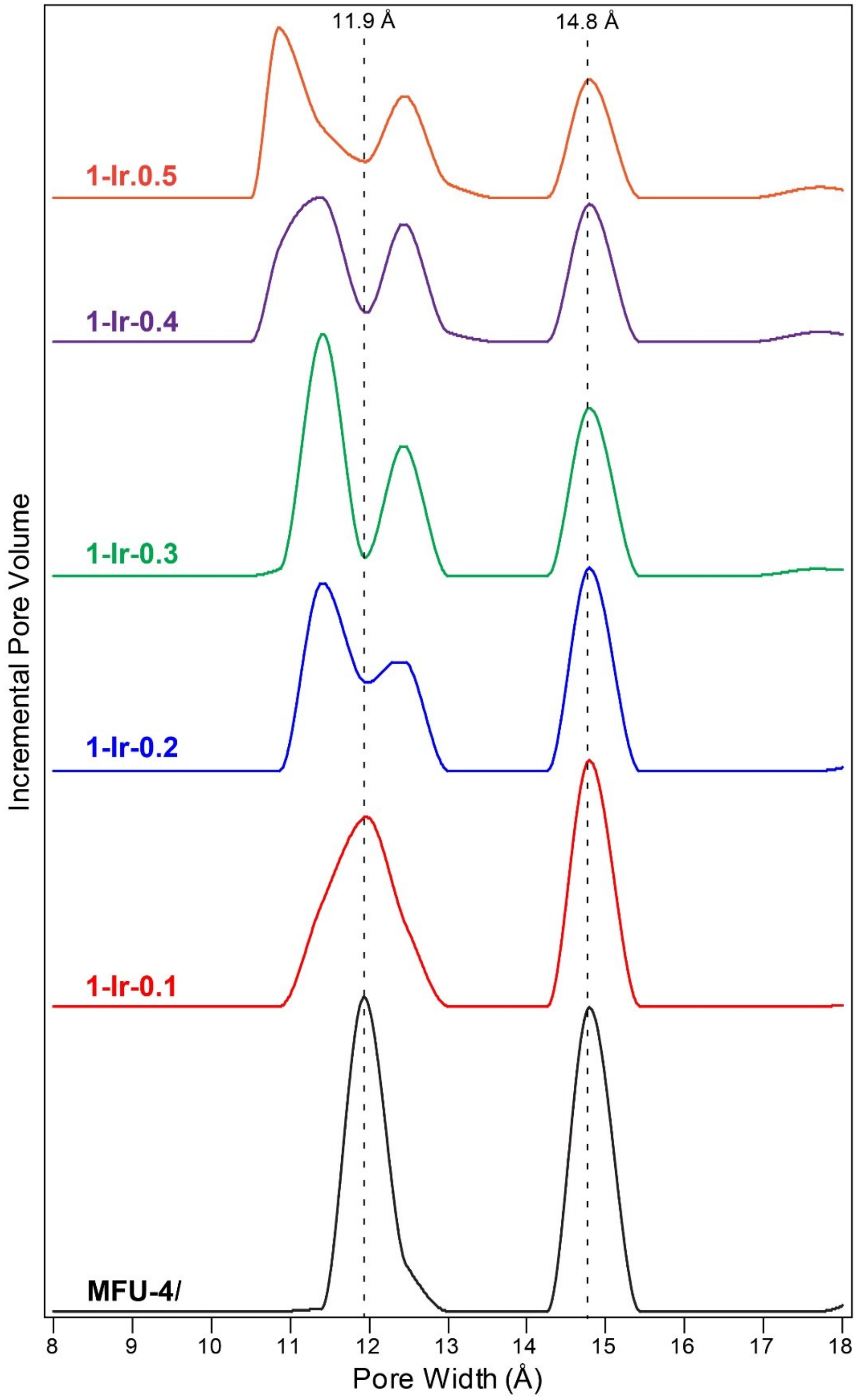

Figure S10. DFT Pore size distributions (2D-NLDFT, Carbon Slit, N_2, 77K) of **1-Ir-*x***.

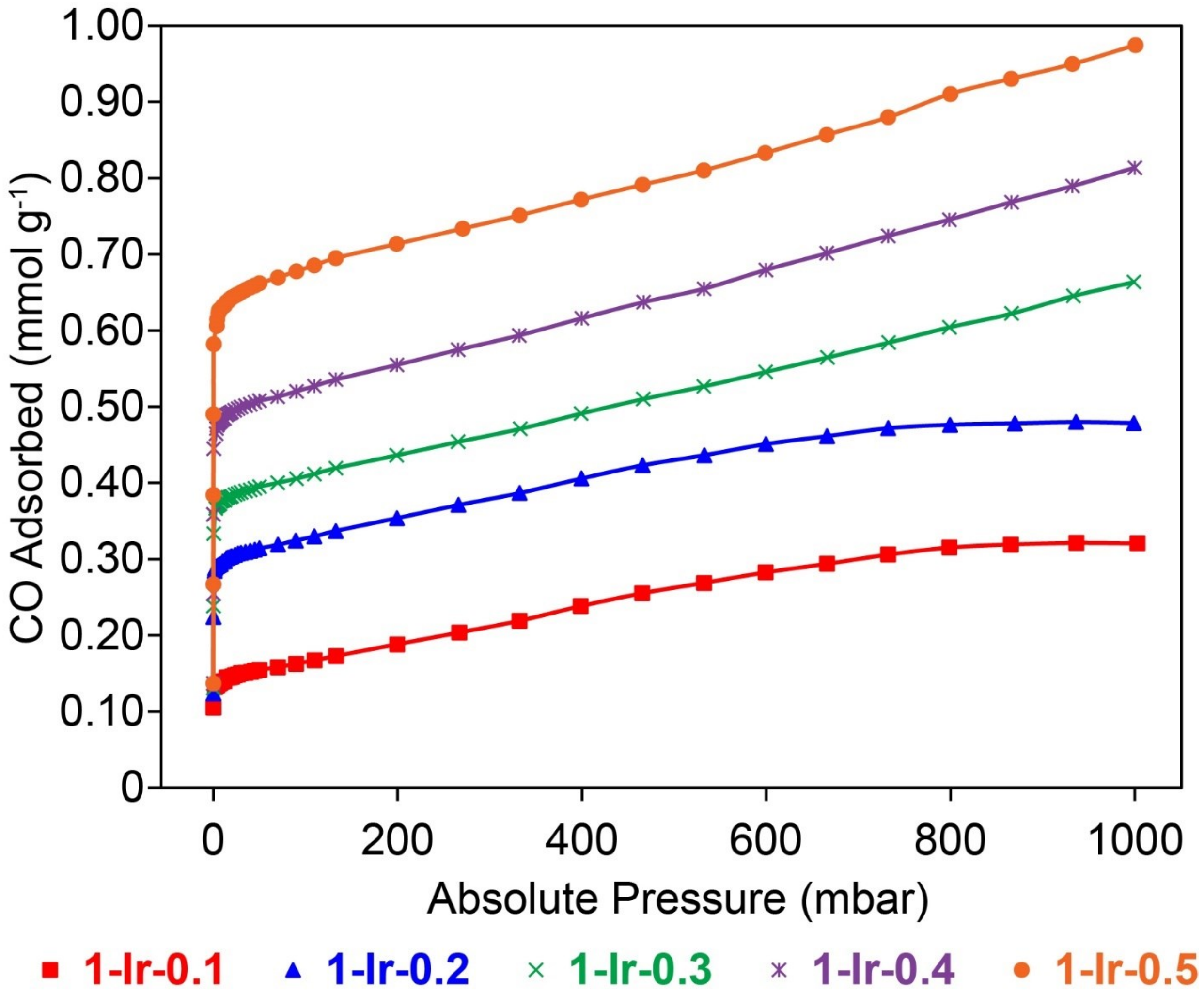

Figure S11. CO gas adsorption isotherms (300 K) of **1-Ir-*x***.

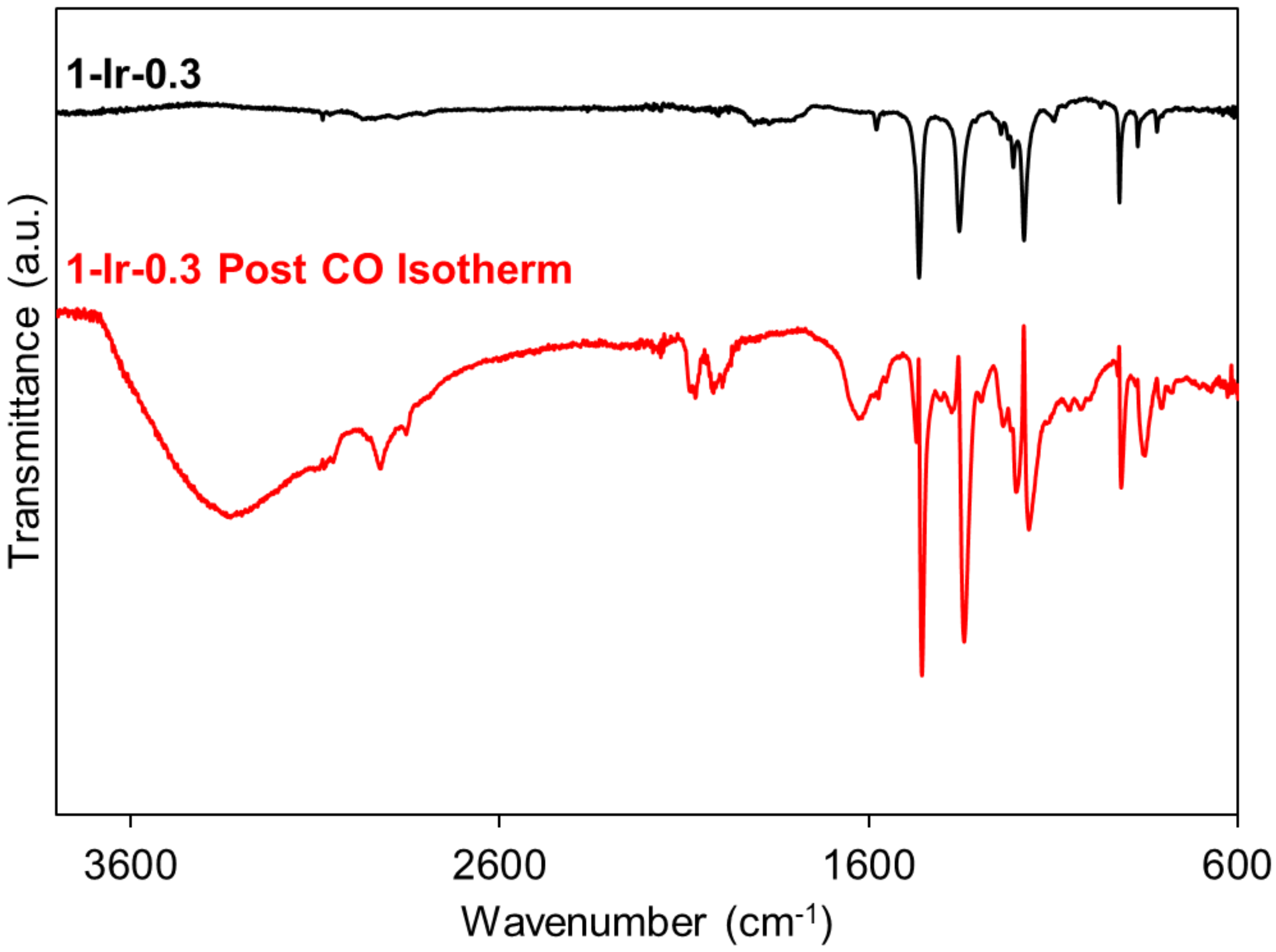

Figure S12. ATR-IR spectrum of **1-Ir-0.3** and **1-Ir-0.3** after CO gas adsorption analysis.

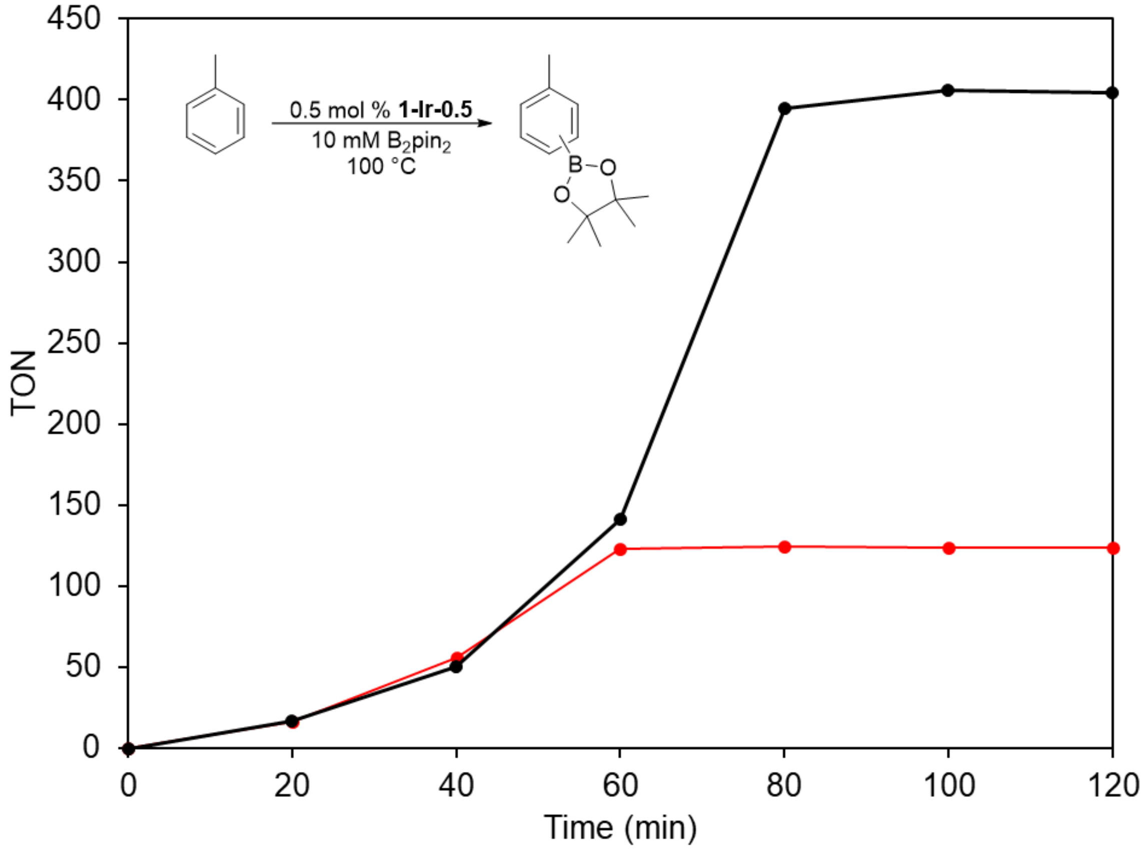

Figure S13. Hot filtration test with **1-Ir-0.5**.

The MOF catalyst was removed by filtration after 60 min (red), demonstrating a halt in catalytic turnover compared to the control reaction (black).

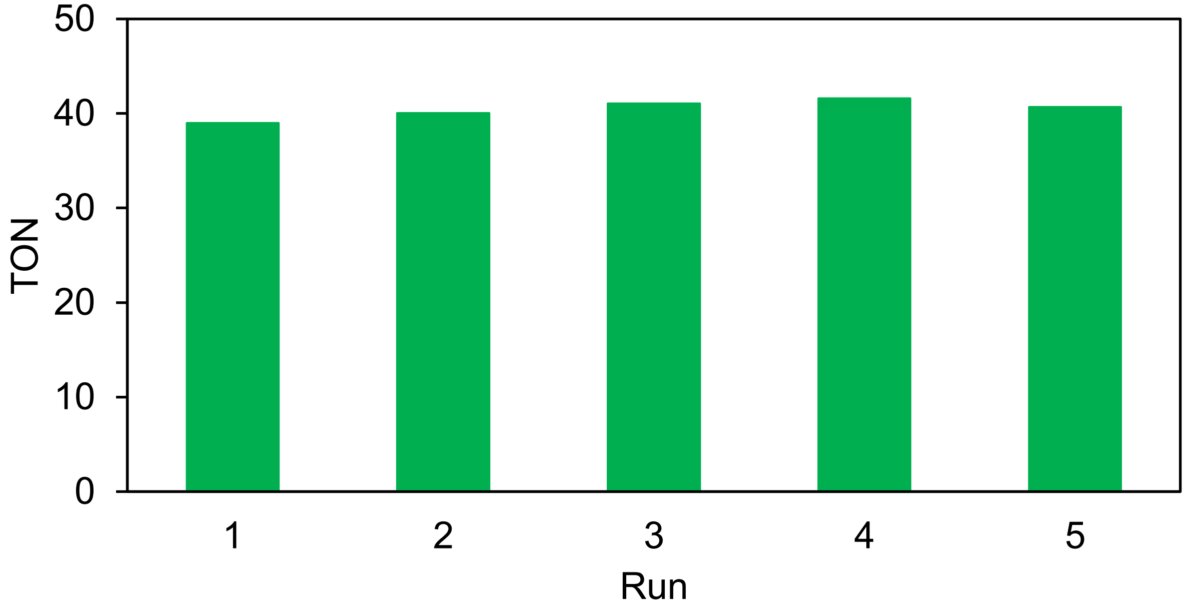

Figure S14. Recycling experiment (80 min) using 5 mol % **1-Ir-0.5** for the C–H borylation of toluene.

Each reaction run used B_2pin_2 (10 mM) and was stopped after 80 minutes for GC-FID analysis and recycling.

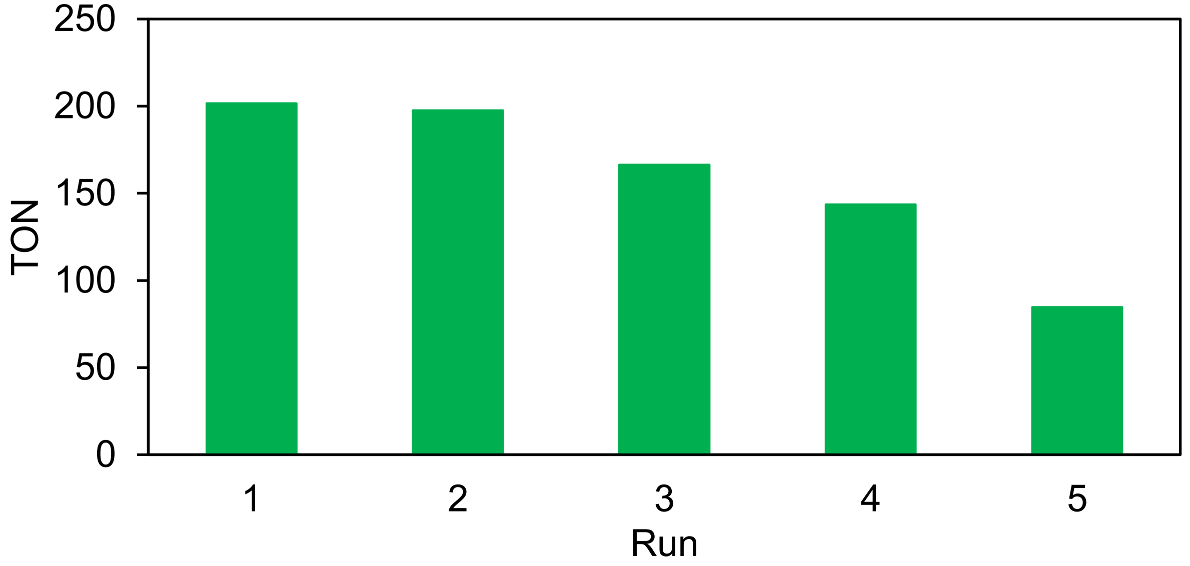

Figure S15. Recycling experiment (80 min) using 1 mol % **1-Ir-0.5** for the C–H borylation of toluene.

Each reaction run used B_2pin_2 (10 mM) and was stopped after 80 minutes for GC-FID analysis and recycling.

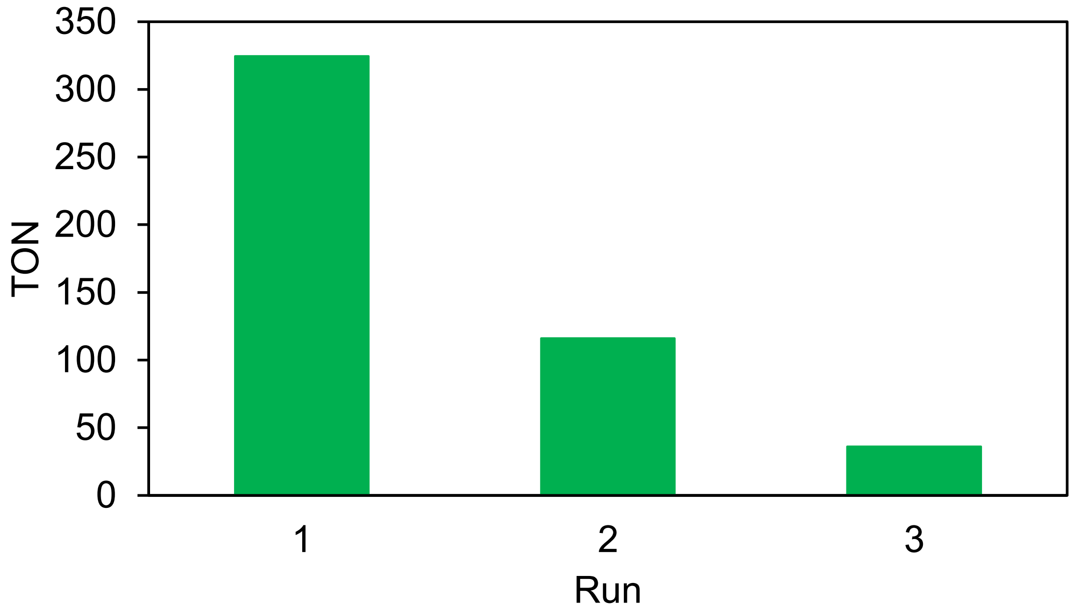

Figure S16. Recycling experiment (80 min) using 0.5 mol % **1-Ir-0.5** for the C–H borylation of toluene.

Each reaction run used B_2pin_2 (40 mM) and was stopped after 80 minutes for GC-FID analysis and recycling.

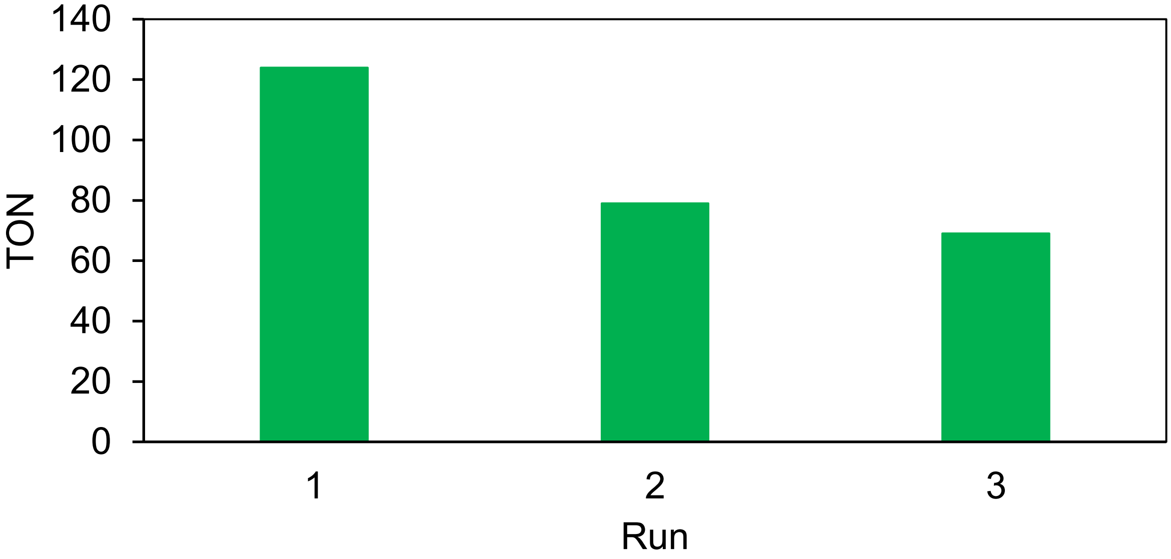

Figure S17. Recycling experiment (30 min) using 0.5 mol % **1-Ir-0.5** for the C–H borylation of toluene.

Each reaction run used B_2pin_2 (10 mM) and was stopped after 30 minutes for GC-FID analysis and recycling.

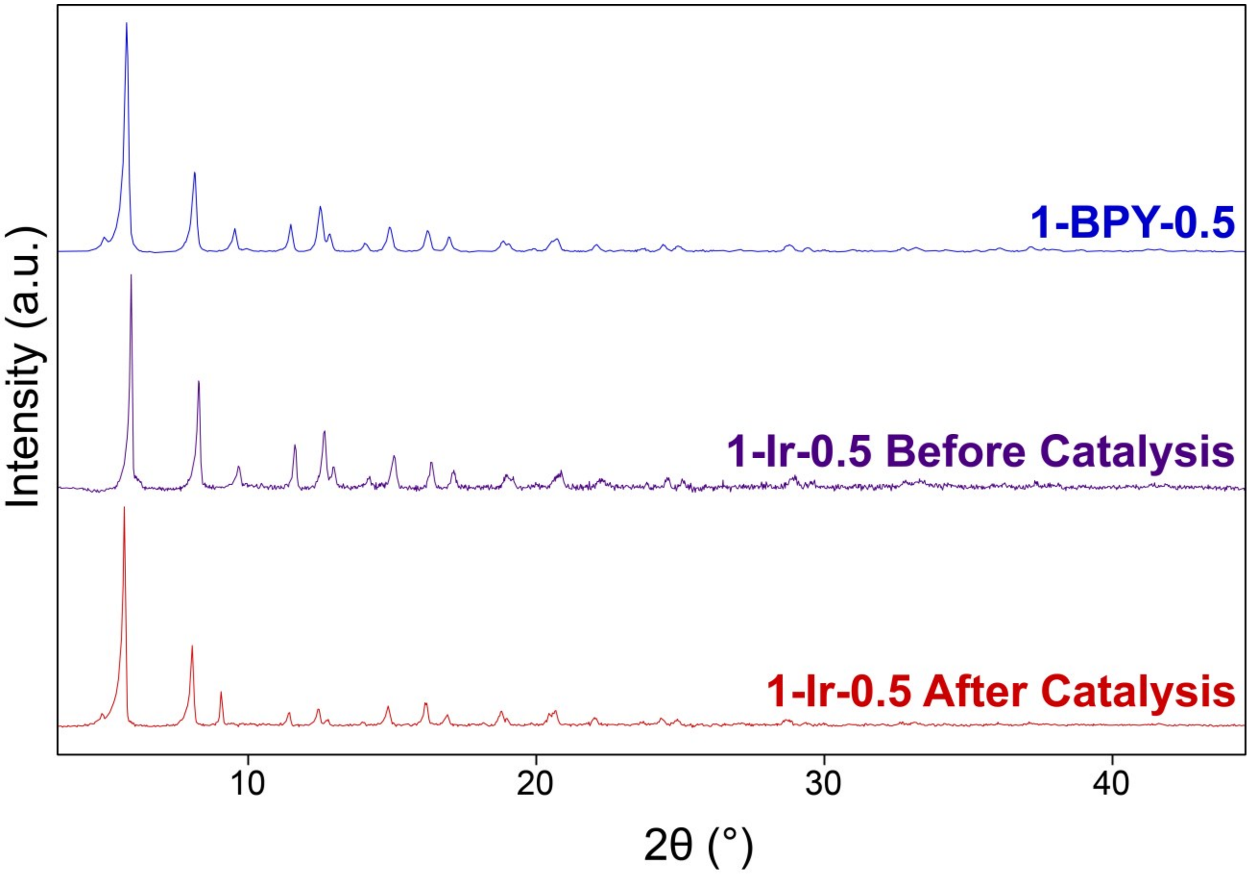

Figure S18. Powder X-ray diffraction patterns of **1-Ir-0.5** before and after catalysis.

The PXRD pattern for **1-Ir-0.5** After Catalysis was collected using the MOF catalyst isolated after Run 5 of the 1 mol % recyclability study.

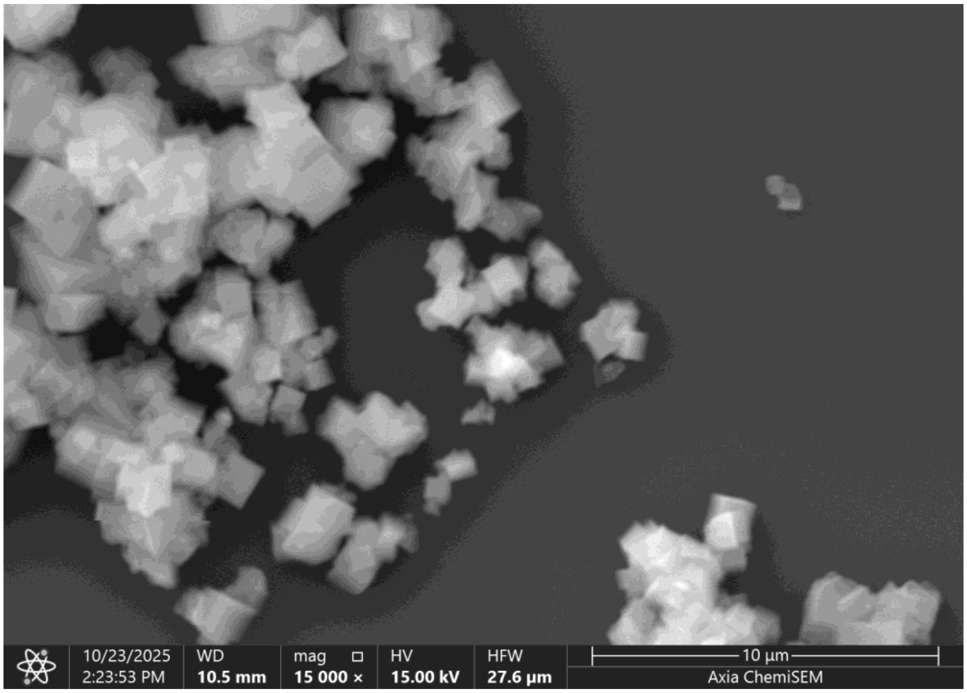

Figure S19. SEM image of 1-Ir-0.5 prior to catalysis.

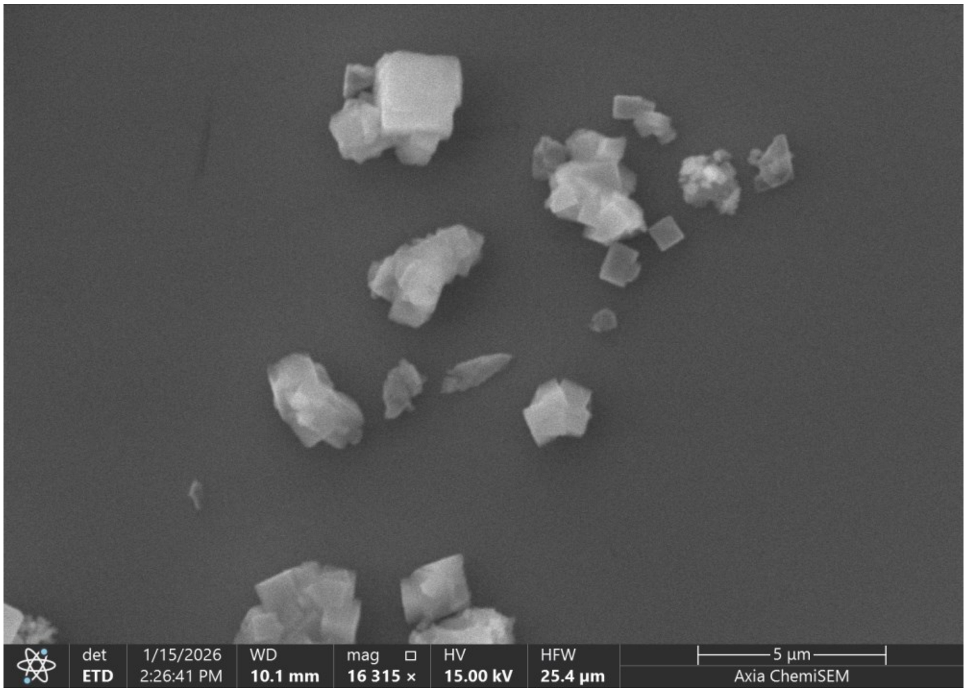

Figure S20. SEM image of 1-Ir-0.5 after 1 mol % recycling study.

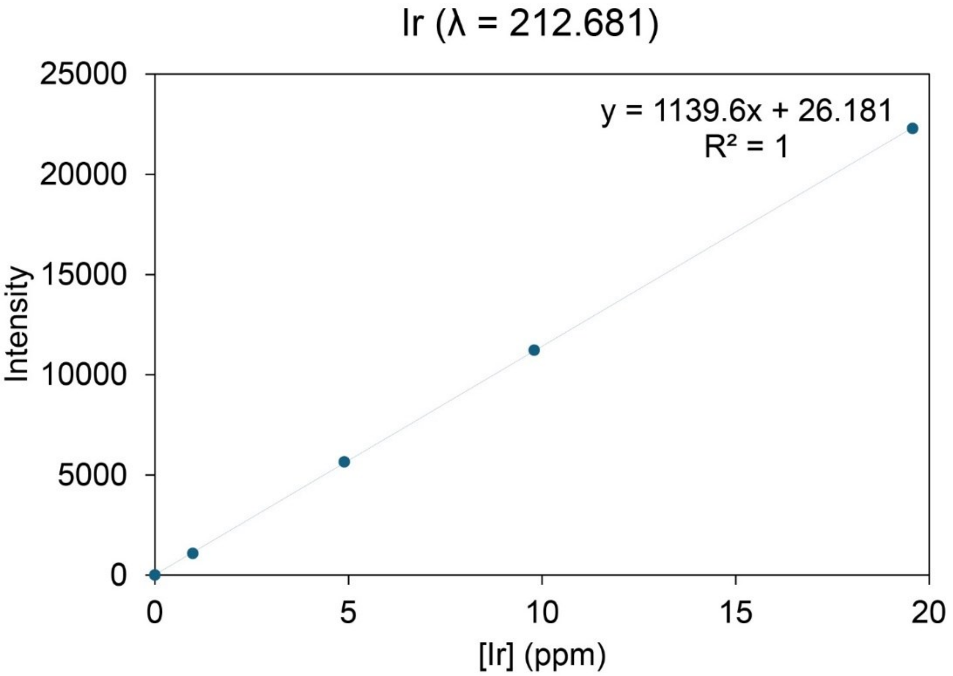

Figure S21. Iridium ICP-OES calibration curve.

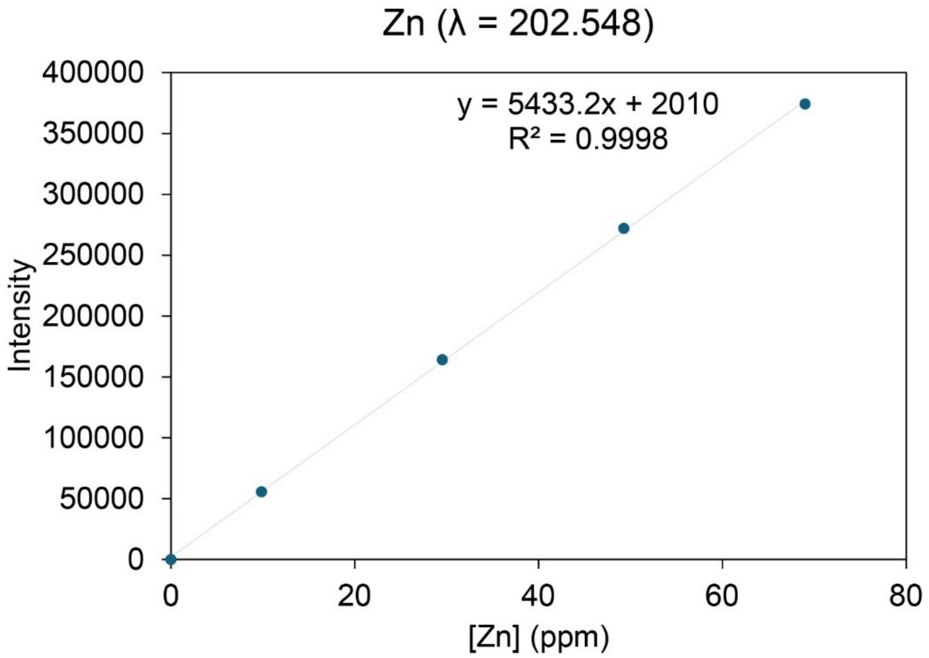

Figure S22. Zinc ICP-OES calibration curve.

Table S1. ICP-OES results for **1-Ir-*x***.

Entry	MOF Sample	Ir/Zn Expected	Ir/Zn Determined
1	**1-Ir-0.1**	0.020	0.022
2	**1-Ir-0.2**	0.040	0.045
3	**1-Ir-0.3**	0.060	0.064
4	**1-Ir-0.4**	0.080	0.085
5	**1-Ir-0.5**	0.100	0.116
6	**1-Ir-0.5** After Catalysis Run 1	0.100	0.101
7	**1-Ir-0.5** After Catalyst Recycling	0.100	0.101

Table S2. Comparison of MOF-supported Iridium C–H Borylation Catalysts

**Catalyst (mol% Ir)[a]**	**Substrate**	**Temp (°C)**	**Time (h)**	**TON**	**Reference**
**1-Ir-0.5** (0.12 mol%)	Toluene	100	20	1,560[b]	**This work**
UiO-67-bpy-[Ir(cod)]BF_4 (0.065 mol %)	Toluene	80	24	1,477	1
mPT-MOF-Ir (0.1 mol%)	Toluene	115	18	2,000[b]	2
Bpy-UiO-Ir (0.5 mol%)	Toluene	100	15	186	3
Bpy-PMO-Ir(OMe)(cod) (1.5 mol%)	Toluene	80	12	188	4
MFU-dppmg-Ir-0.3 (0.1 mol%)	Toluene	100	72	778	5
**Neat Benzene Substrate:**					
UiO-67-bpy-[Ir(cod)]BF_4 (0.065 mol%)	Benzene	80	24	1,538	1
UiO-67-bpy-[Ir(cod)]BF_4 (0.021 mol %)	Benzene	80	24	1,477[b]	1
Ir-BPy-PMO (0.125 mol%)	Benzene	80	24	728	6
mPT-MOF-Ir (0.1 mol%)	Benzene	115	18	2,000[b]	2
mBPV-MOF-Ir (0.1 mol%)	Benzene	115	16	2,000[b]	2
**Homogeneous Catalysts:**					
[Ir(COD)Cl]$_2$/dtbpy (0.003 mol%)	Benzene	100	24	24,800	7

[a]Reactions were preformed in neat arene substrate
[b]B_2pin_2 was used in a stochiometric amount with respect to arene, doubling the maximum TON
cod = 1,5-cyclooctadiene

Table S3. C–H Borylation of toluene in solvent**.**

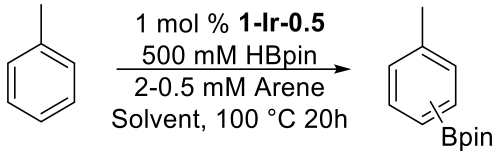

Entry	Solvent	$[\text{Arene}]_0$	TON[b]
**1**	Heptane	2 M	77
**2**	Heptane	1 M	55
**3**	Heptane	0.5 M	31
**4**[c]	Cyclohexane	2 M	76
**5**[c]	Cyclohexane	1 M	32
**6**[c]	Cyclohexane	0.5 M	17
**7**	1,4-Dioxane	1 M	77
**8**	1,4-Dioxane	0.5 M	23

[a]Reaction conditions: HBpin (500 mM), arene (0.5-2 M), catalyst (0.0008 mmol Ir), solvent, 100 °C. [b]Turnover numbers (TON) are reported as total borylated products per Ir site. TONs were determined by GC-FID with respect to an internal standard (hexamethylbenzene). [c]Reaction was performed at 80 °C.

Details of DFT Calculations

All calculations were performed with Orca v6.0.1[8,9] and/or GFN2-xTB.[10] A truncated model of the A-type pore (cubic cage) containing the embedded bpydc-Ir(Bpin)$_3$ catalyst was constructed using reported crystallographic coordinates for MFU-4*l*.[11] This initial model was subject to full geometry optimization using GFN2-xTB, and the resulting structure was used to construct starting models of the arene adducts (AA) and oxidative addition (OA) products for C–H activation at the *meta* and *para* positions of PhTIPS. Subsequent gas phase geometry optimizations were performed using a multiscale QM/xTB approach implemented in Orca. The boundary region was defined as the C–C bonds of the bpydc carboxylate groups (Figure S23). The bpy-Ir(Bpin)$_3$ fragments with bound substrate (124 atoms) were assigned to the high-level region and treated with the r^{2}SCAN-3c composite DFT method.[12] The remaining cage atoms (676 atoms) were assigned to the low-level region and treated with GFN2-xTB. Hydrogen link atoms were automatically placed at the truncated C–C bonds of the QM/xTB boundary. The coordinates of the low-level region were fixed during geometry optimizations by defining the high-level region with the ActiveAtoms keyword.

The optimized *meta*/*para*-AA and OA geometries were used as reactants and products for Nudged Elastic Band (NEB) transition state searches.[13] Transition state guesses were identified from the climbing images generated in the NEB calculations. Transition state optimizations (OPTTS) were performed by following the C–H bond breaking modes using the TS_mode keyword. Frequency calculations confirmed the presence of imaginary modes associated with the transition states (-800 cm^{-1} for TS$_{meta}$ and -801 cm^{-1} for TS$_{para}$).

Frequency calculations revealed several low-energy, imaginary vibrational modes for each of the catalyst-embedded cage structures. The presence of a few imaginary modes is not unusual for large systems treated with multiscale methods and/or fixed-atom constraints.[14] Although it has been proposed that small imaginary frequencies can be neglected or inverted in some cases, in the present case they were found to have a significant effect on the thermochemical calculations.[15] Attempts to remove the imaginary frequencies by tightening the geometry optimization criteria or increasing the DFT integration grid were unsuccessful.

To remedy the imaginary modes, the high-level regions (bpyIr(Bpin)$_3$ + substrate) of the optimized structures were extracted from the MOF cage with hydrogen link atoms (Figure S23, shown in red). The resulting fragments were subject to constrained geometry optimizations using r^{2}SCAN-3c and the CPCM(heptane) implicit solvation model. The cartesian coordinates were held fixed for the core atoms, including Ir, B, N$_{bpy}$, H$_{Ir–H/C–H}$, Si, O$_{Si–O}$, and C$_{Ar}$ of the substrate (Figure S23, shown in blue). This approach maintains the strain induced by the confined environment of the MOF cage while allowing the peripheral groups (*i.e.* Bpin and Si(iPr)$_3$) to relax. Overlays of the high-level regions from the cage-embedded and constrained catalyst optimizations are shown in Figure S25. The core structures of the catalyst+substrate, including the arene bending angles at the Ir–C bonds, are nearly unchanged and the only noticeable differences are in the peripheral

Bpin and TIPS groups. The resulting optimized structures exhibited, at most, one low-energy imaginary frequency (< 10 cm^{-1}) associated with methyl group rotations.

To interrogate the effects of MOF-induced strain, the *meta*/*para*-AA, TS, and OA structures were also subject to geometry optimizations (r^2SCAN-3c/CPCM(heptane)) in the absence of the cage and without any constraints. Overlays of the fully relaxed and constrained structures are shown in Figure S26.

Final electronic single point energies were computed at the ωB97M-V/def2-QZVP level[16,17] with matching auxiliary basis sets,[18] a large-core ECP on Ir,[19] and the CPCM(heptane) implicit solvation model.[20,21]

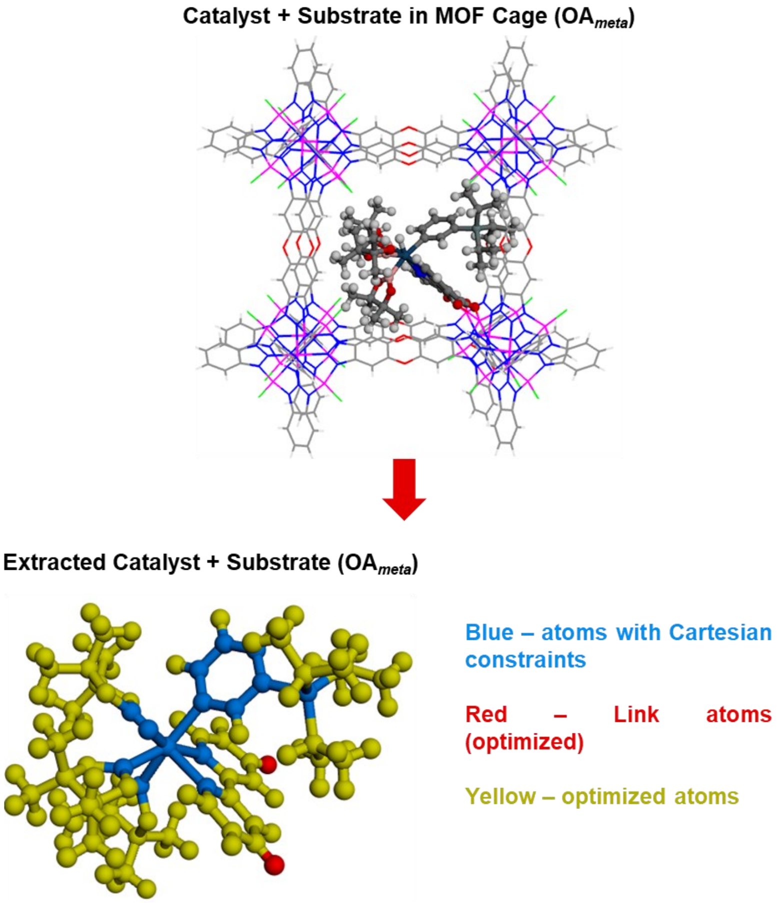

Figure S23. Structure of OA_{meta} embedded in MOF cage (Top) and extracted structure with link atoms for constrained optimization (Bottom).

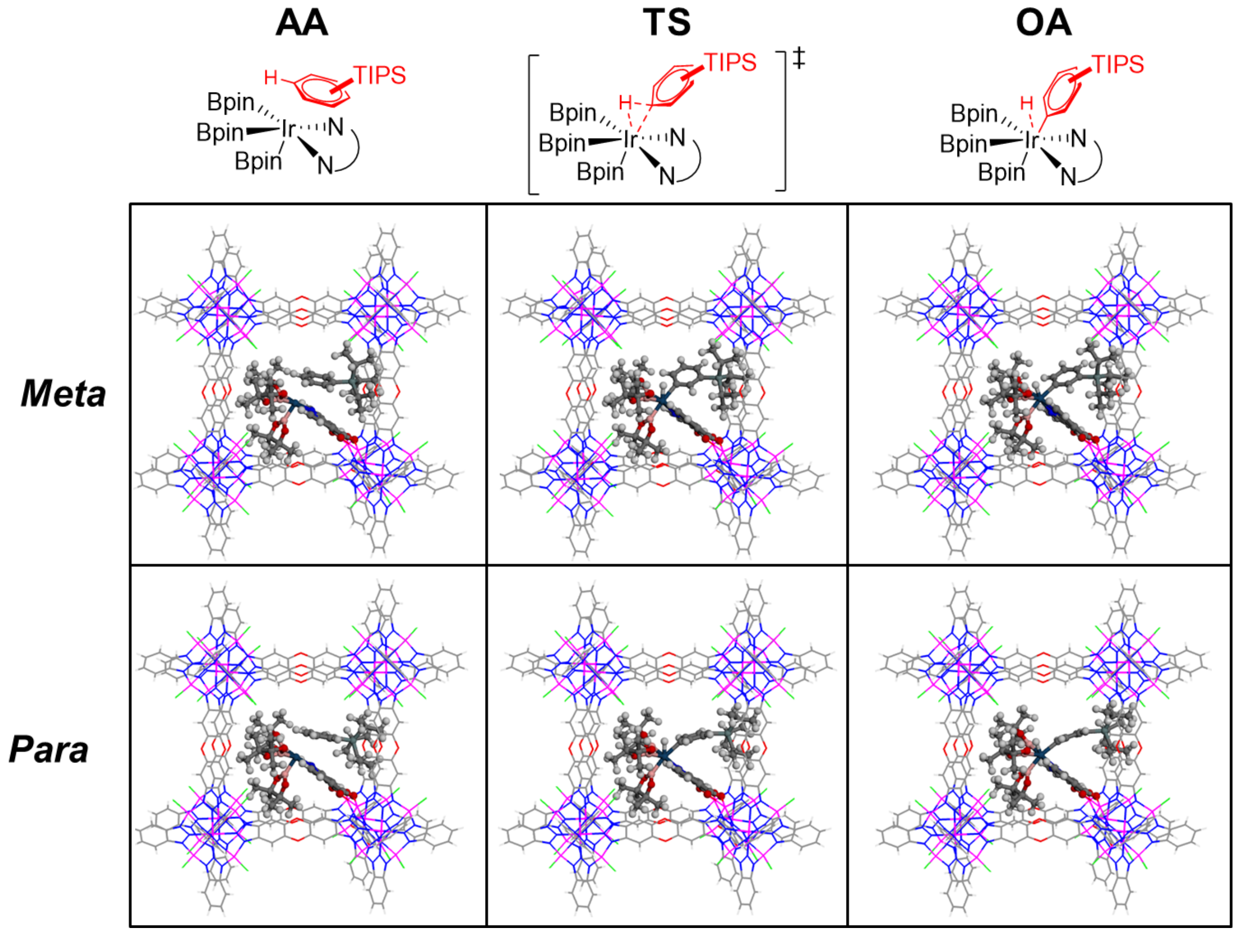

Figure S24. DFT-optimized structures embedded in the MOF cage.

The structures include the arene adducts (AA), transition states (TS), and oxidative addition (OA) products for C–H bond activation at the *meta* and *para* sites of PhTIPS.

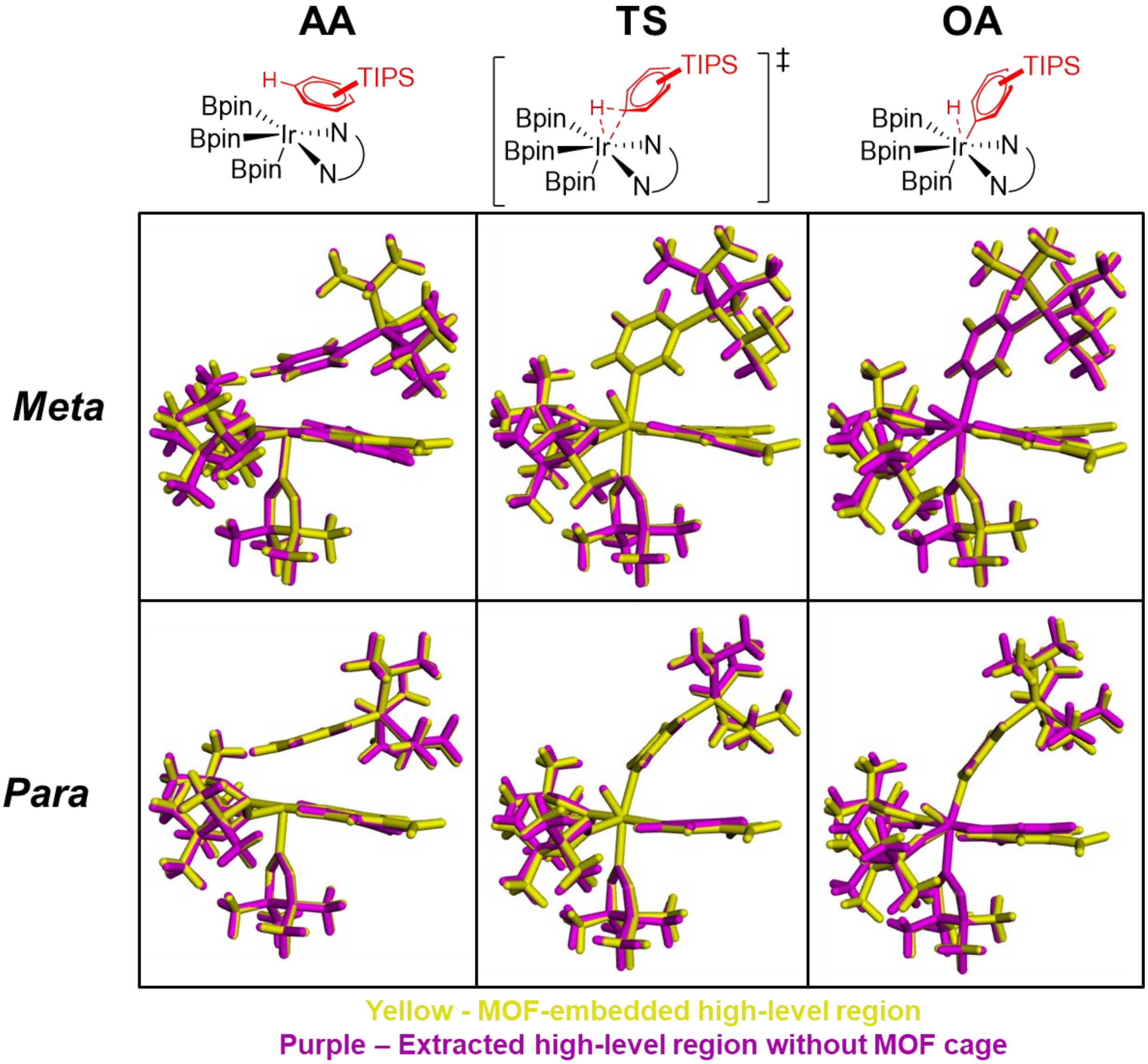

Figure S25. Overlay of AA, TS, and OA structures optimized within the MOF cage (yellow) followed by constrained optimization without the MOF cage (purple).
For the constrained optimization (purple), the cartesian coordinates were held fixed for the core atoms, including Ir, B, N_{bpy}, $H_{Ir–H/C–H}$, Si, $O_{Si–O}$, and C_{Ar} of the substrate (see blue atoms in Figure S23). The constrained optimization maintains the strain induced by the confined environment of the MOF cage while allowing the peripheral groups (*i.e.* Bpin and $Si(iPr)_3$) to relax, removing spurious imaginary frequencies.

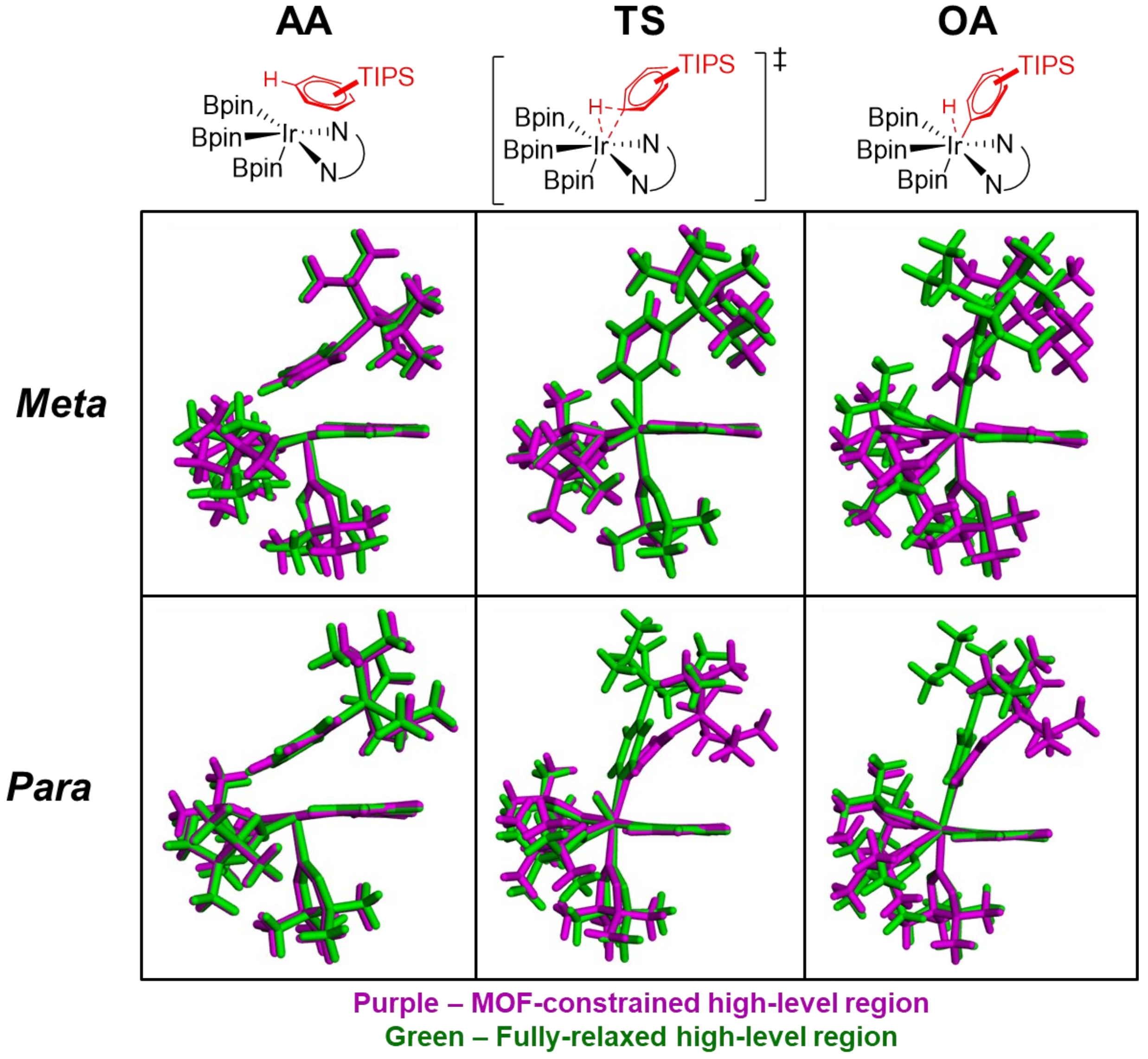

Figure S26. Overlay of DFT-optimized structures of MOF-constrained (purple) and fully relaxed (green) structures.
Structures include the arene adducts (AA), transition states (TS), and oxidative addition (OA) products for C–H bond activation at the *meta* and *para* sites of PhTIPS.

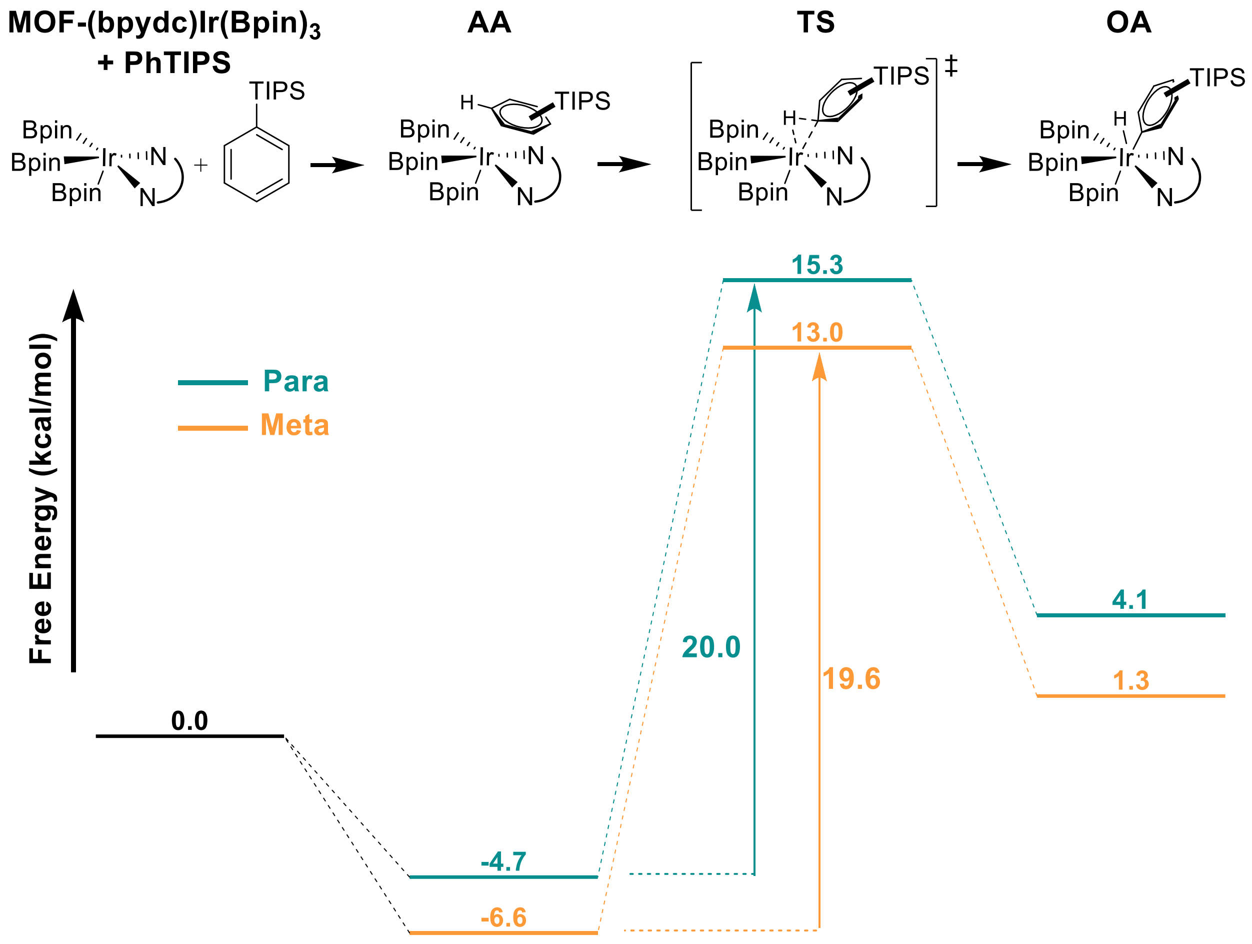

Figure S27. DFT-calculated free energy reaction profiles for *meta* and *para*-C–H activation of PhTIPS with fully relaxed (bpy)Ir(Bpin)$_3$ catalyst and substrate fragments.

¹H NMR Spectra of Substrates

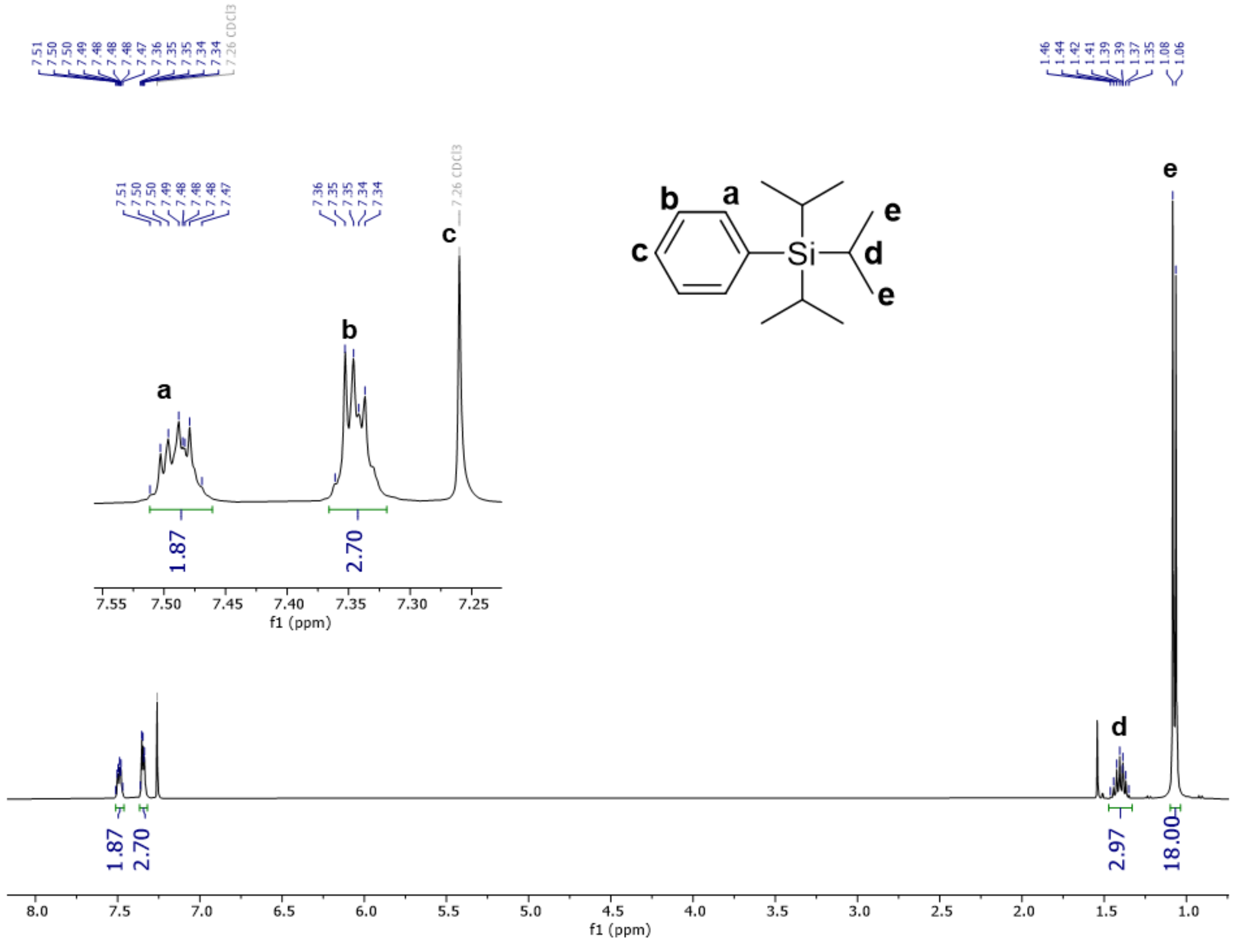

Figure S28. ^{1}H NMR spectrum of phenyl(triisopropyl)silane (PhTIPS). Sample was measured in $CDCl_3$ at 400 MHz.

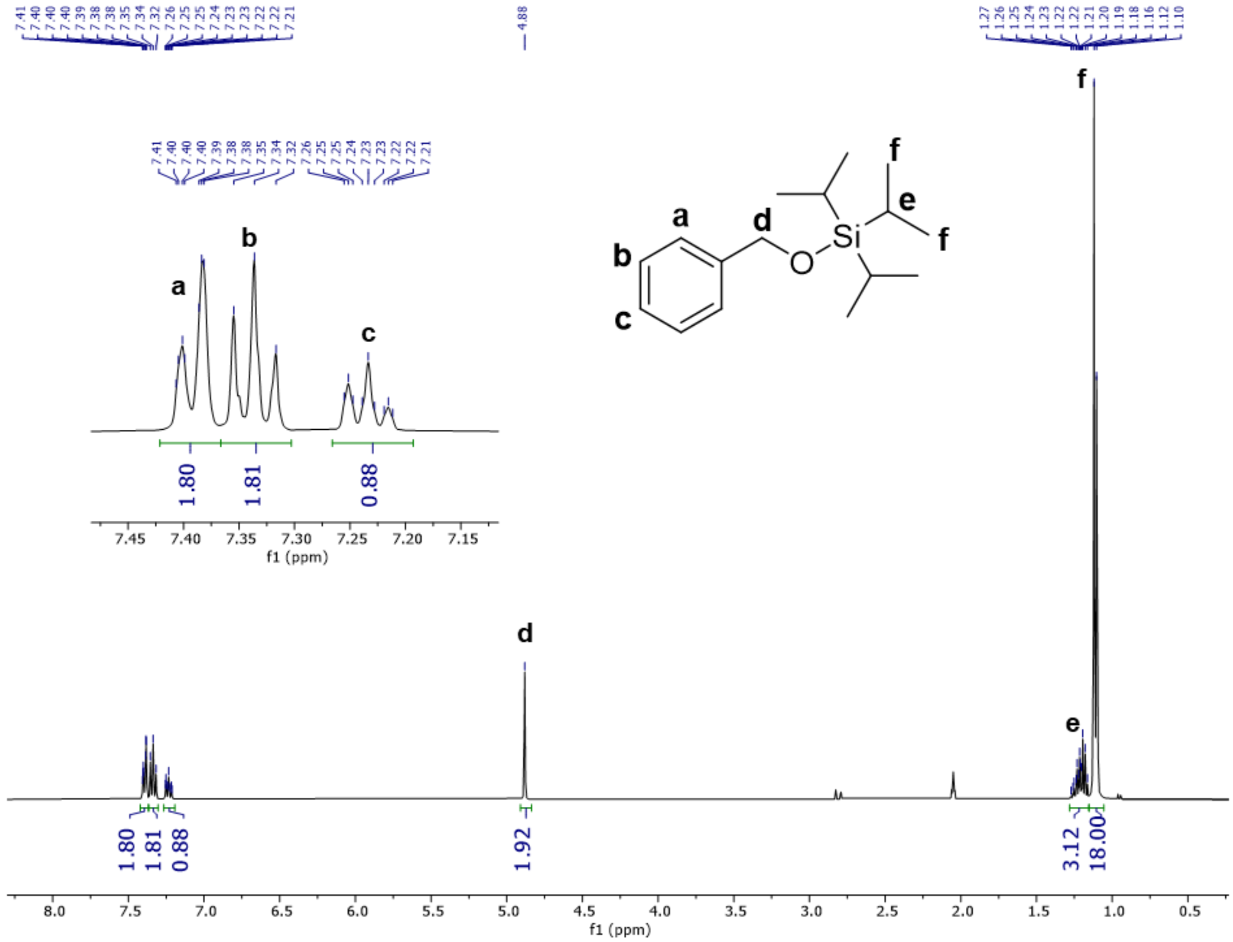

Figure S29. ^{1}H NMR spectrum of benzyl(triisopropyl)silyl ether (BnOTIPS). Sample was measured in acetone-d_6 at 400 MHz.

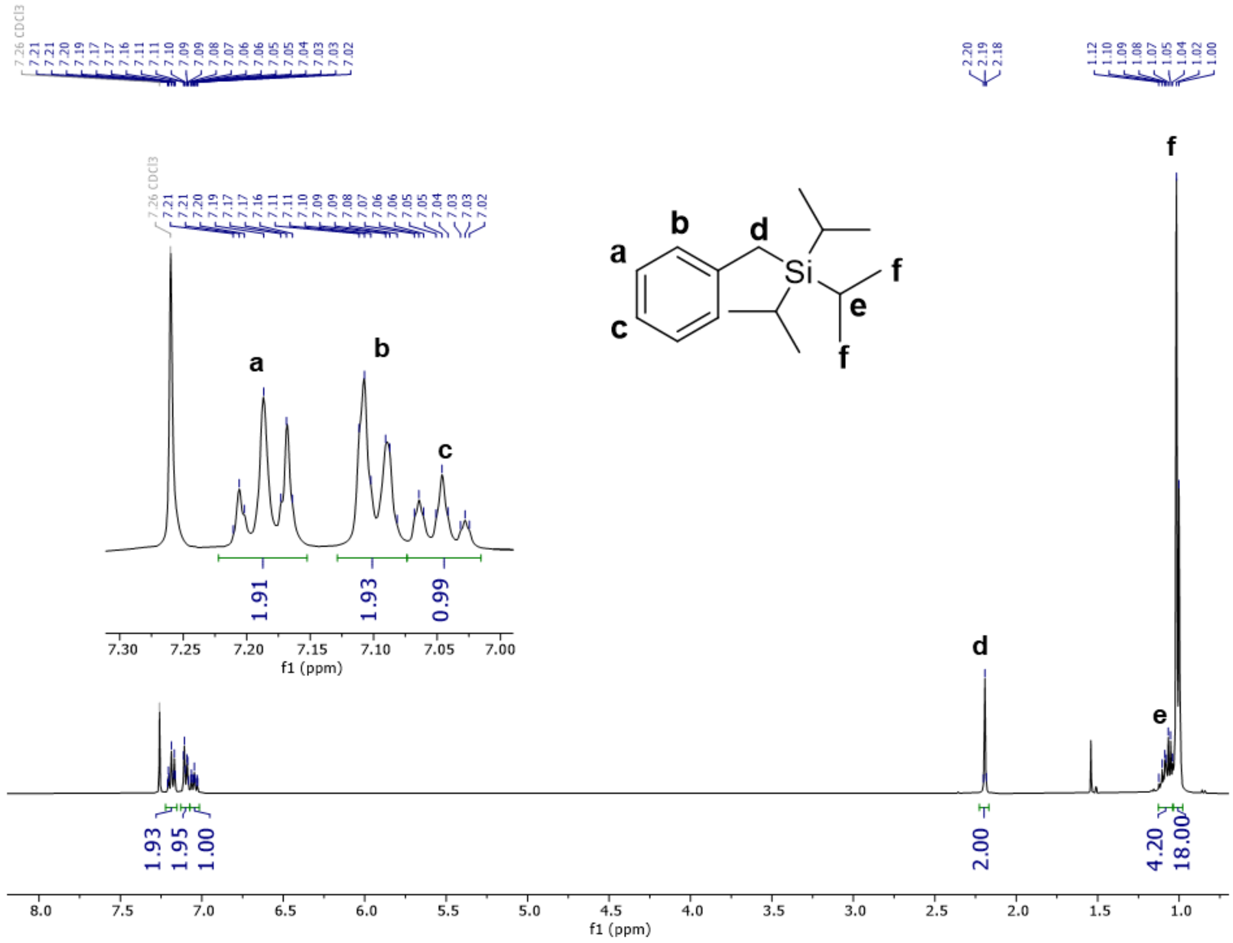

Figure S30. ^{1}H NMR spectrum for benzyl(triisopropyl)silane (BnTIPS). Sample was measured in $CDCl_3$ at 400 MHz.

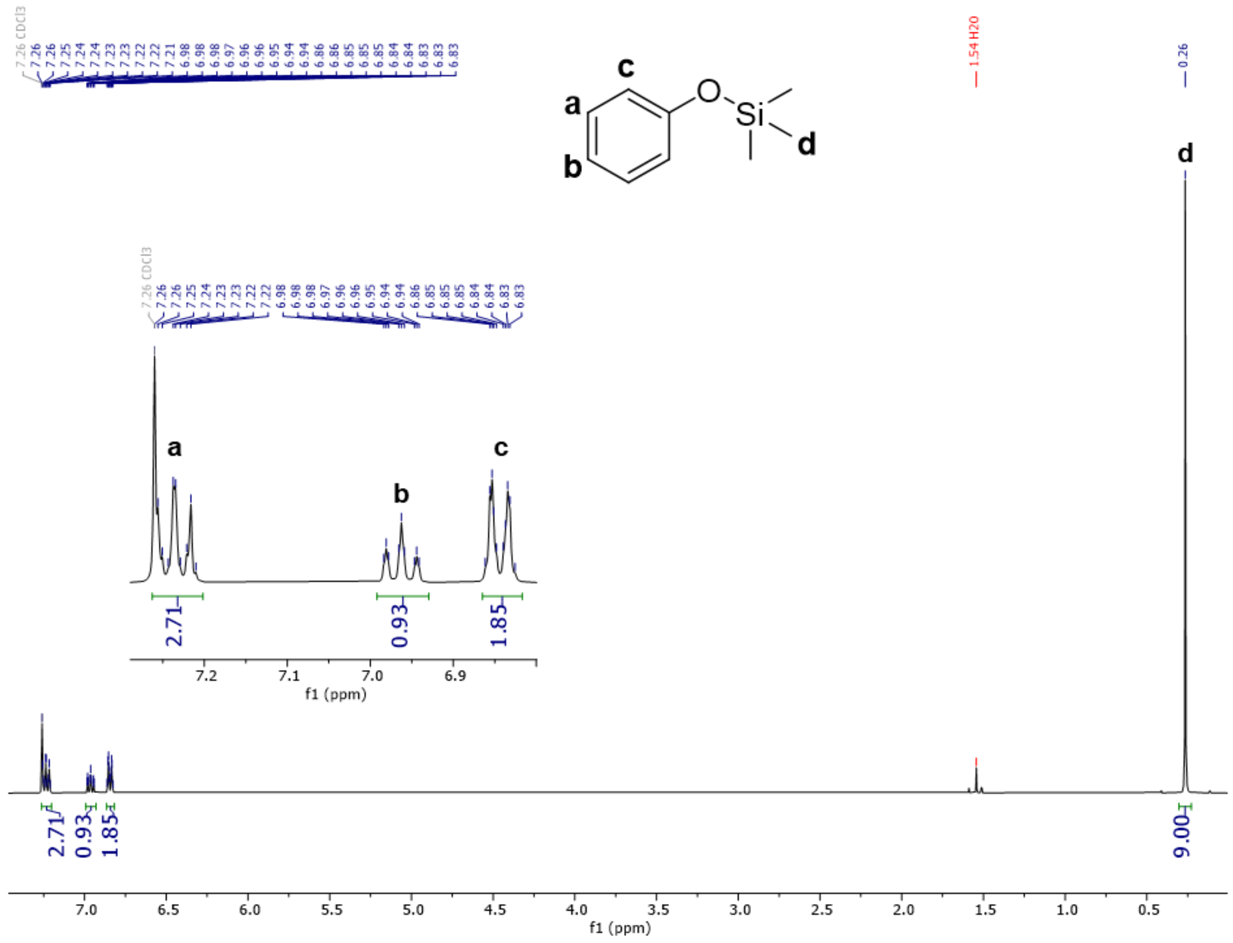

Figure S31. ^{1}H NMR spectrum of phenoxy(trimethyl)silane (PhOTMS). Sample was measured in $CDCl_3$ at 400 MHz.

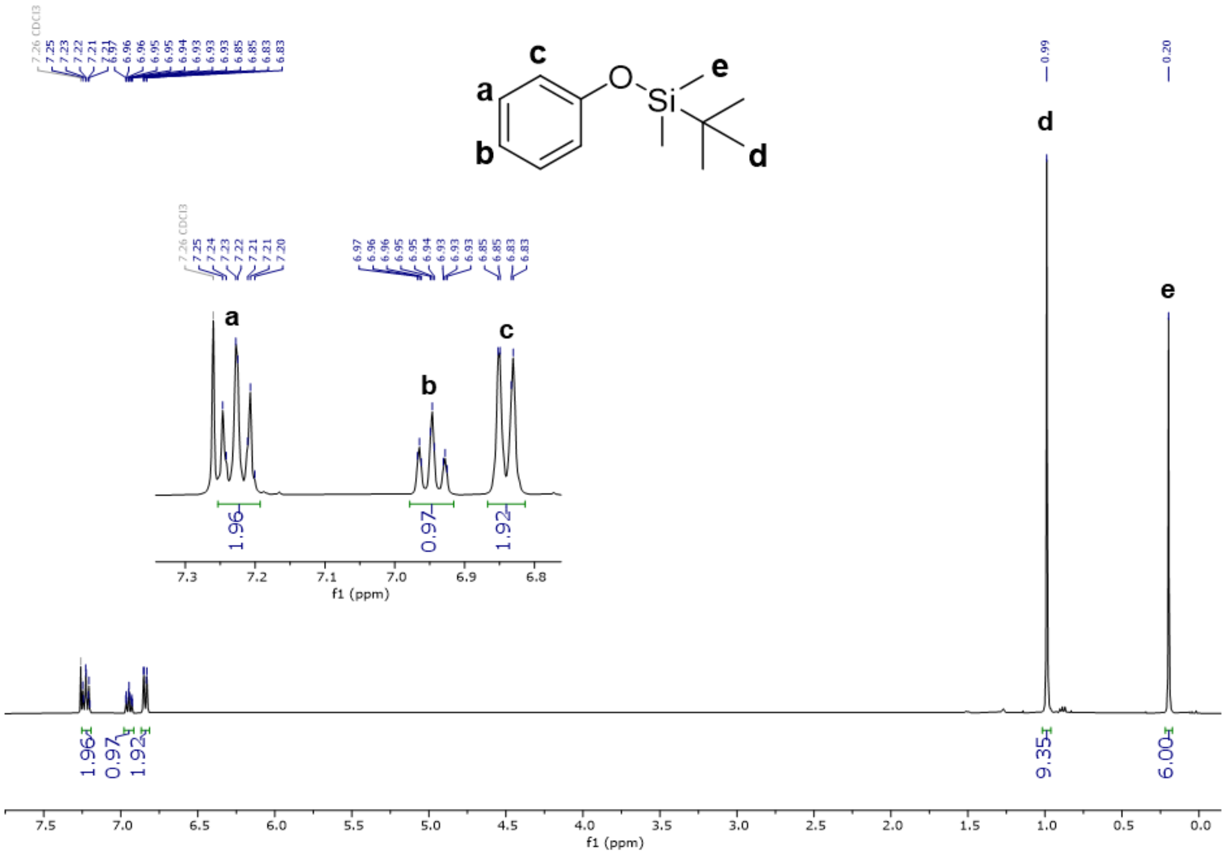

Figure S32. ^{1}H NMR spectrum of phenoxy(*tert*-butyldimethyl)silane (PhOTBDMS). Sample was measured in $CDCl_3$ at 400 MHz.

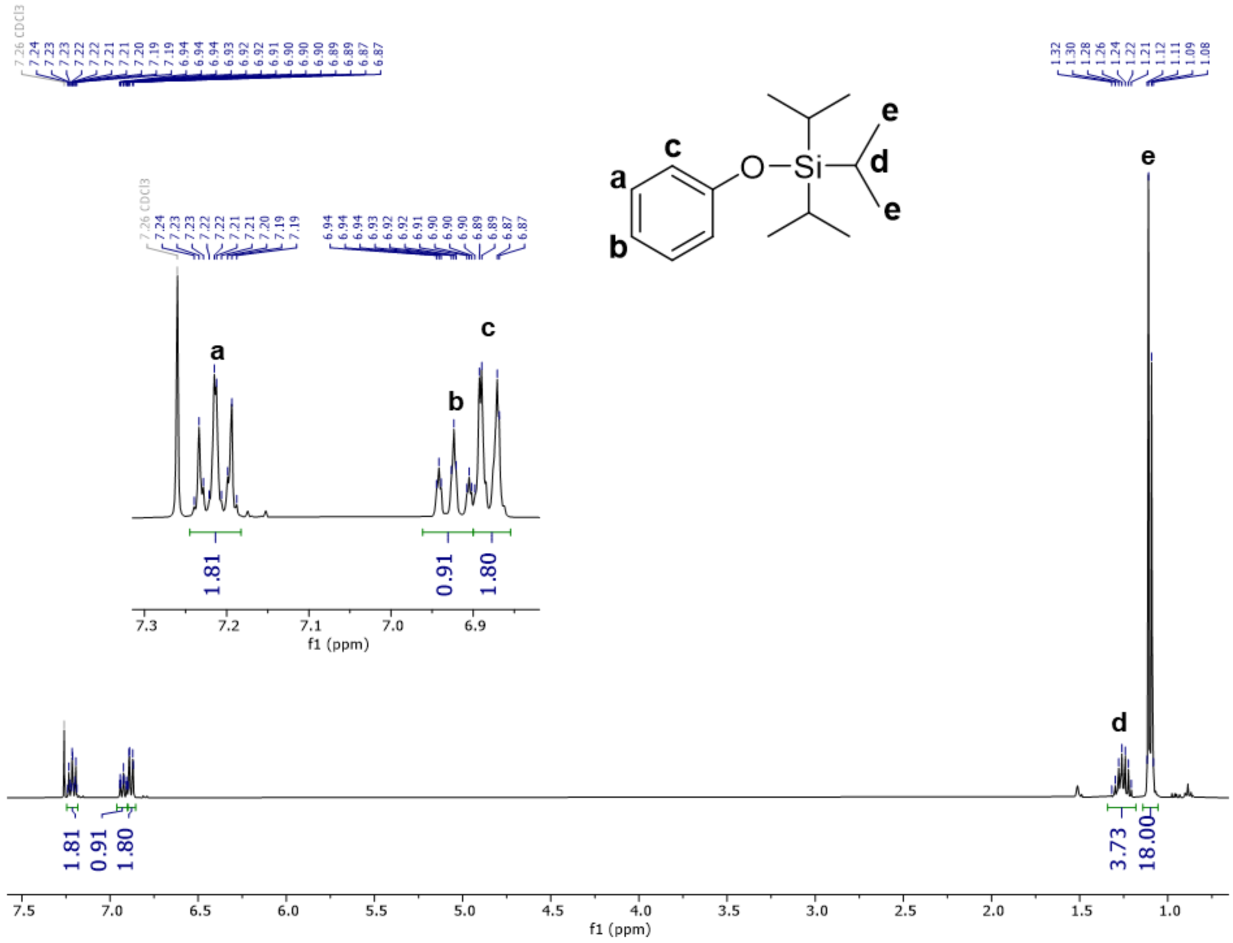

Figure S33. ^{1}H NMR spectrum of phenoxy(triisopropyl)silane (PhOTIPS). Sample was measured in $CDCl_3$ at 400 MHz.

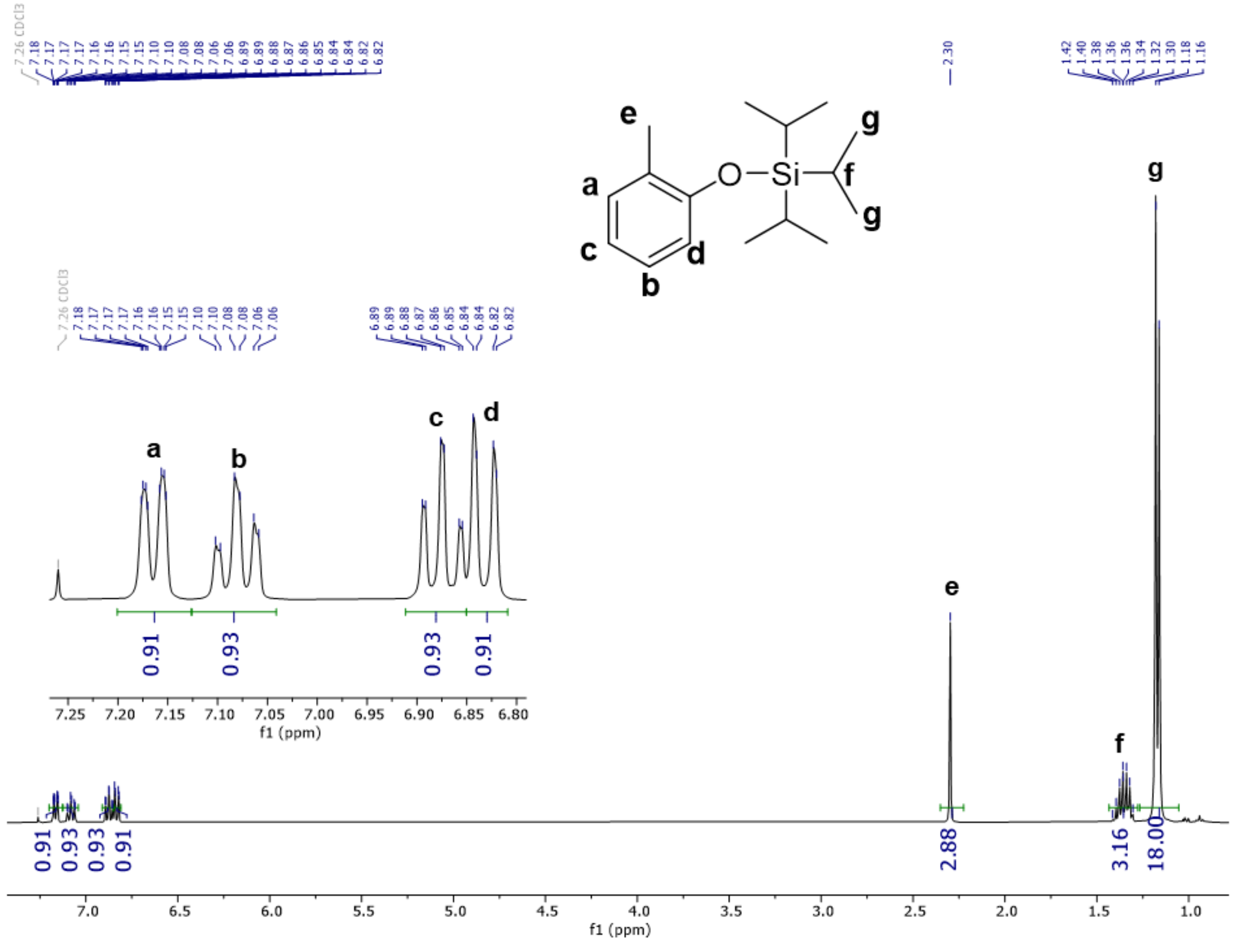

Figure S34. ^{1}H NMR spectrum of 2-methyl-phenoxy(triisopropyl)silane (2-Me-PhOTIPS).
Sample was measured in $CDCl_3$ at 400 MHz.

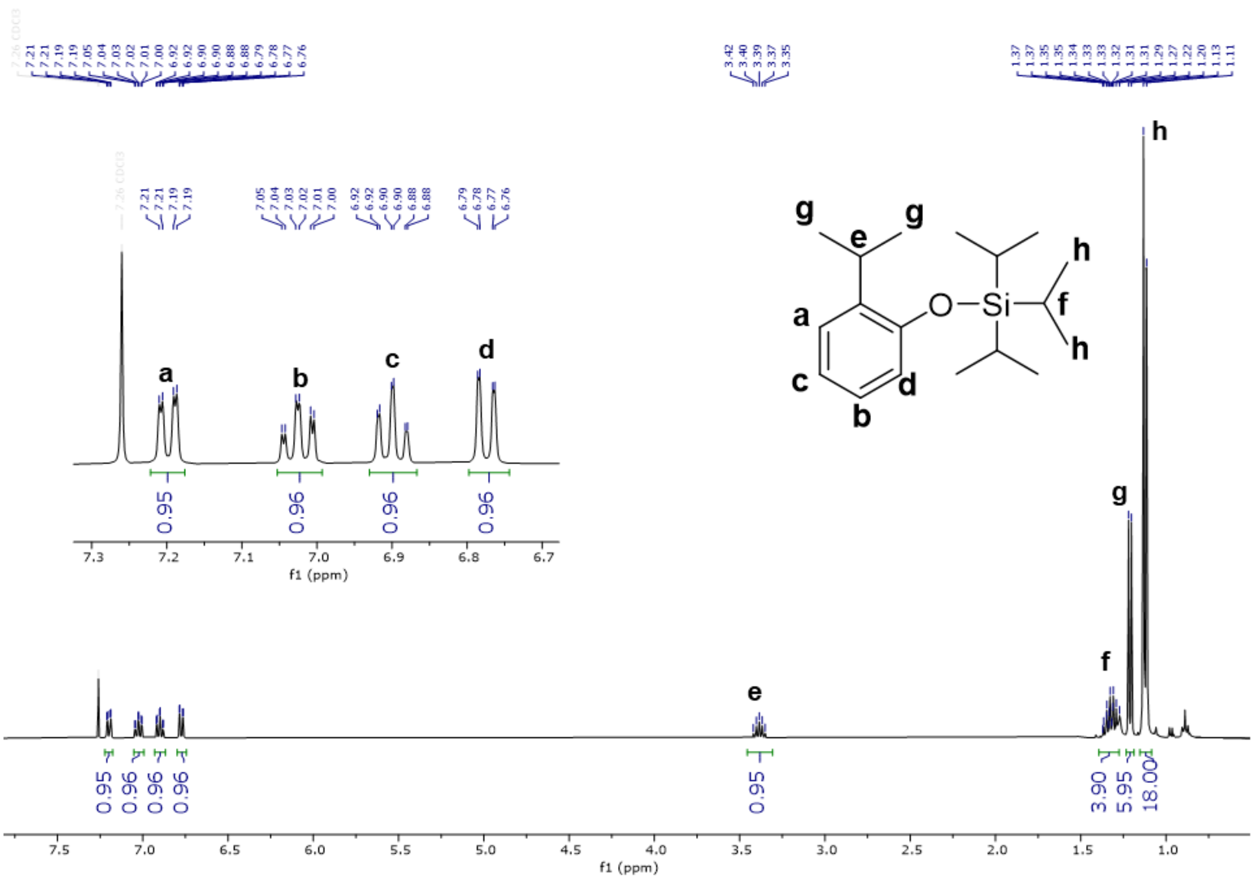

Figure S35. ^{1}H NMR spectrum of 2-isopropyl-phenoxy(triisopropyl)silane (2-iPr-PhOTIPS).
Sample was measured in $CDCl_3$ at 400 MHz.

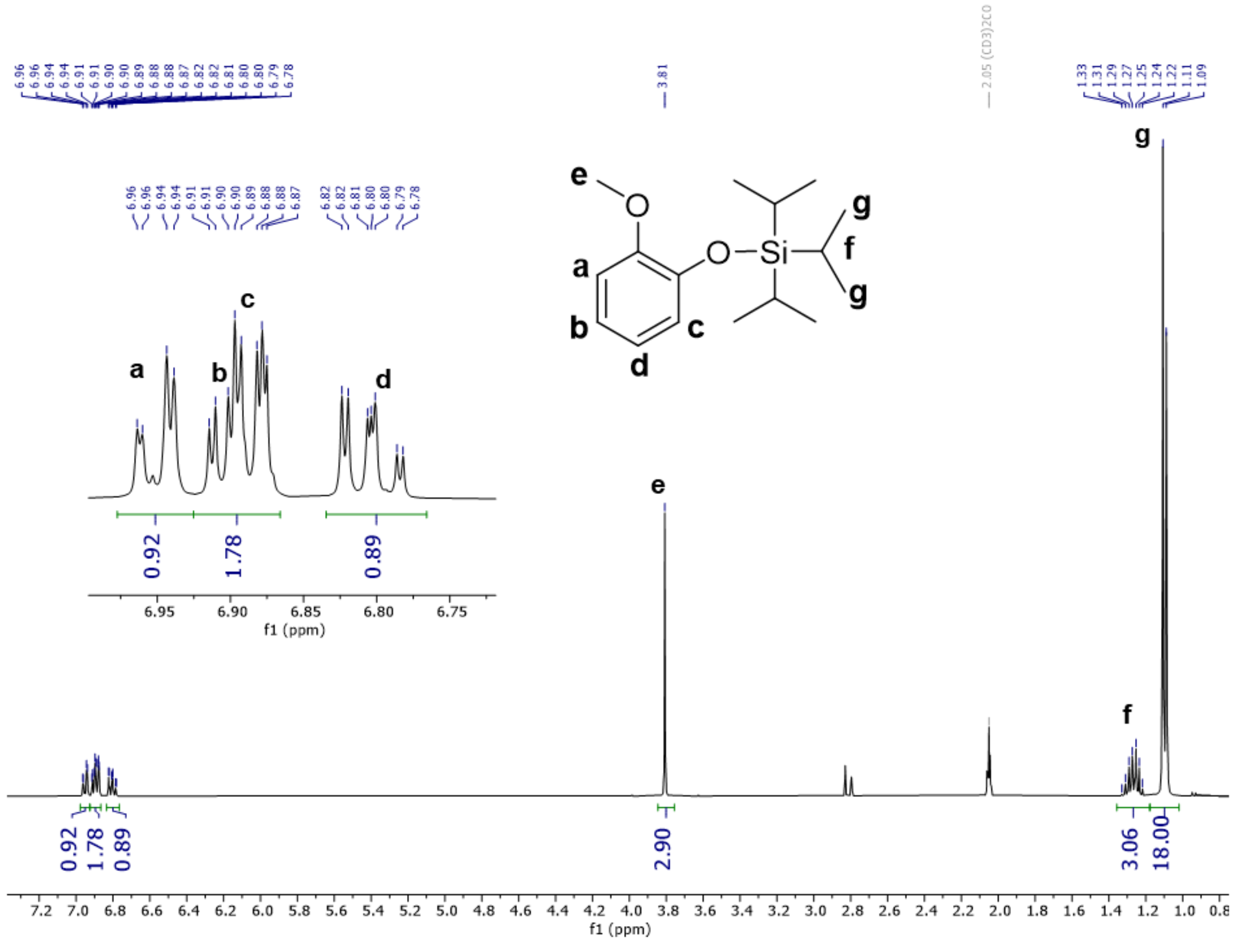

Figure S36. ^{1}H NMR spectrum of 2-methoxy-phenoxy(triisopropyl)silane (2-MeO-PhOTIPS).
Sample was measured in acetone-d_6 at 400 MHz.

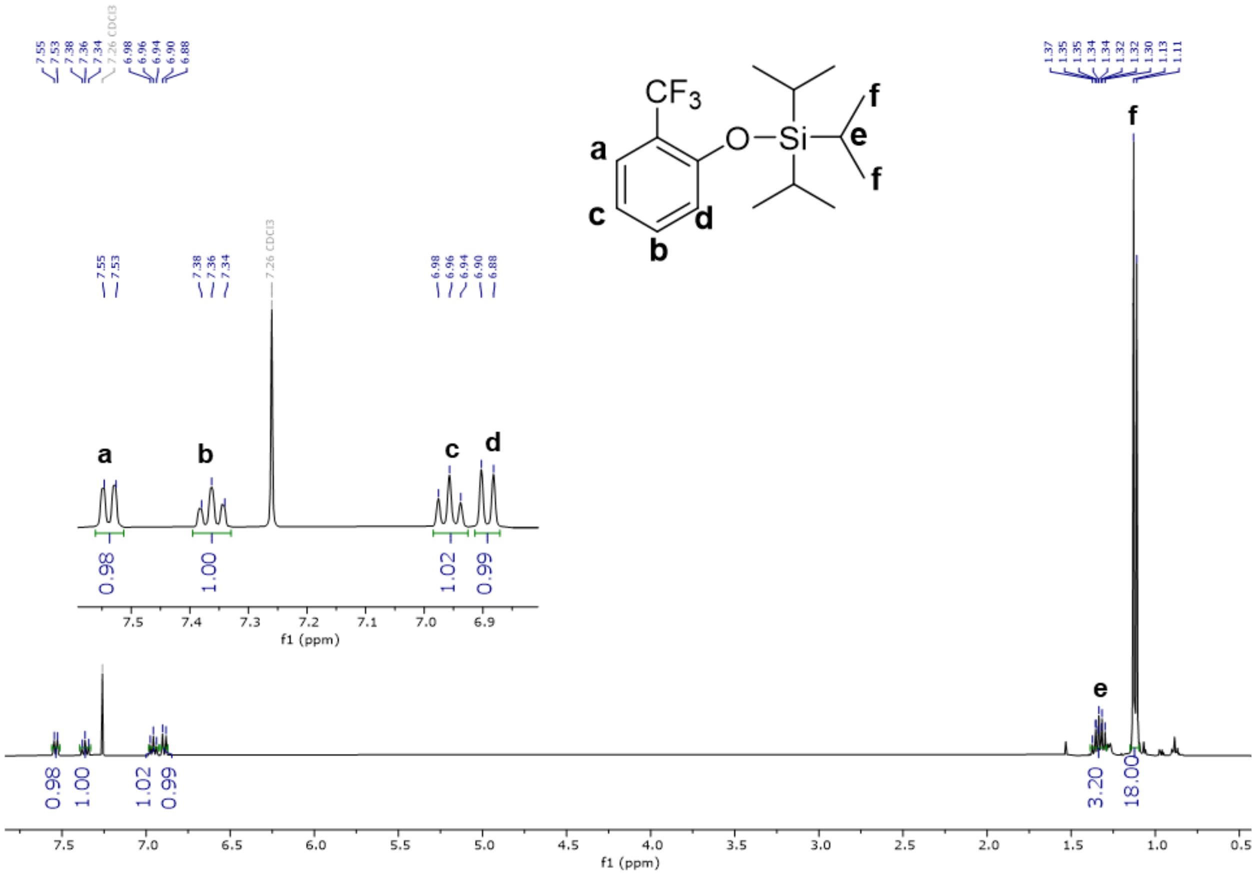

Figure S37. ^{1}H NMR spectrum of 2-trifluoromethyl-phenoxy(triisopropyl)silane (2-CF_3-PhOTIPS).
Sample was measured in $CDCl_3$ at 400 MHz.

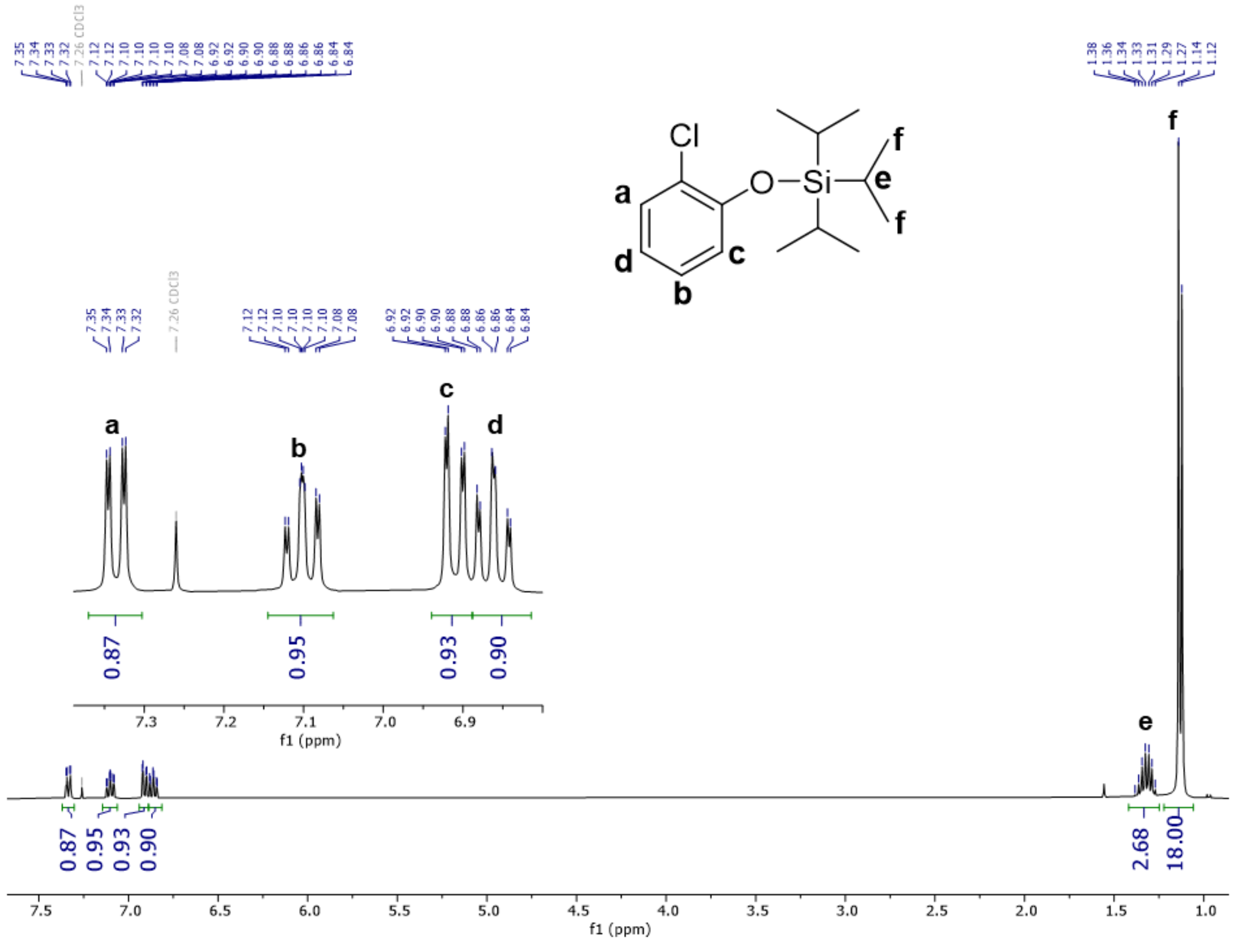

Figure S38. ^{1}H NMR spectrum of 2-chloro-phenoxy(triisopropyl)silane (2-Cl-PhOTIPS). Sample was measured in $CDCl_3$ at 400 MHz.

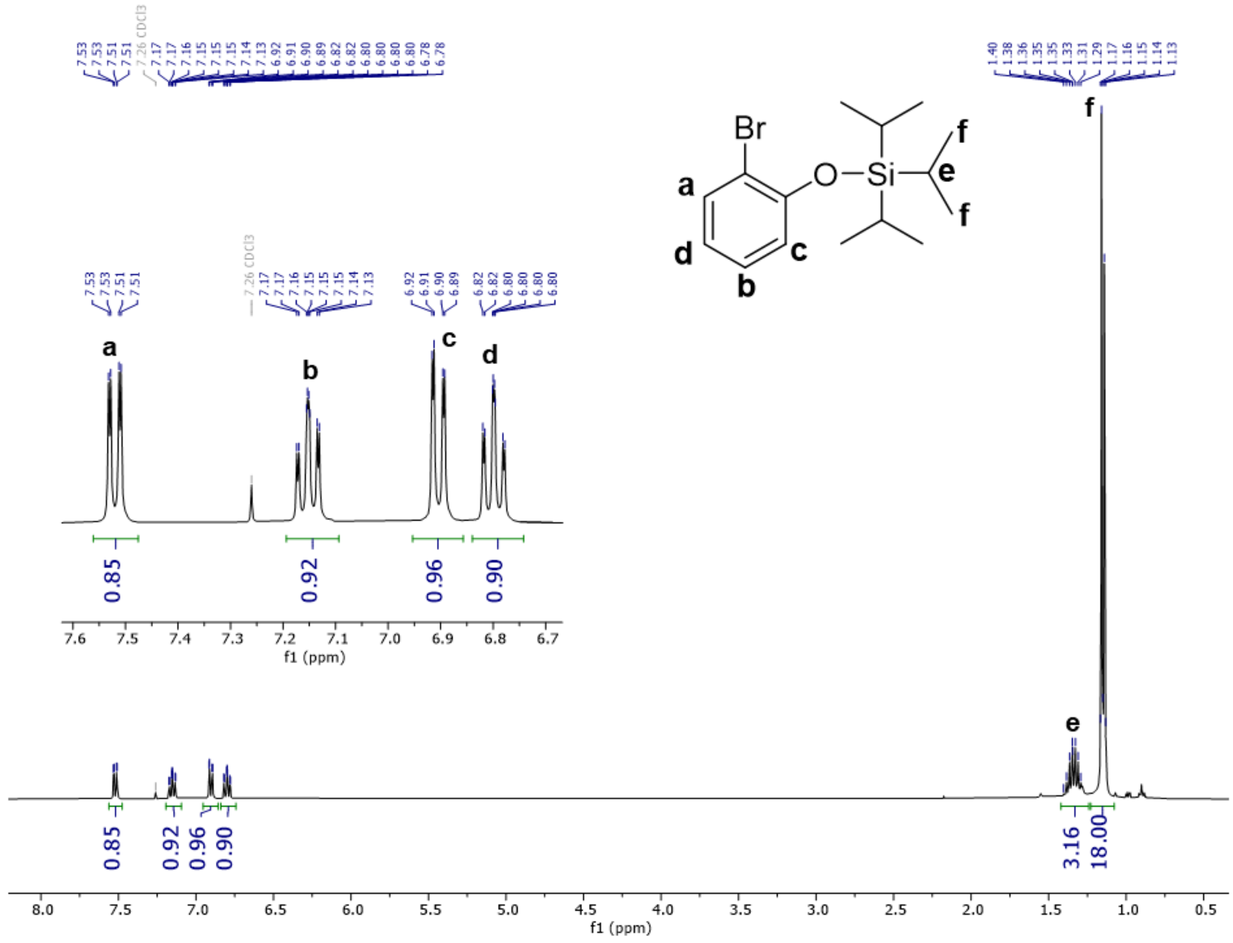

Figure S39. ^{1}H NMR spectrum of 2-bromo-phenoxy(triisopropyl)silane (2-Br-PhOTIPS). Sample was measured in $CDCl_3$ at 400 MHz.

Experimental Details of GC-FID Product Quantification

Chromatogram peak areas for internal standard, substrate, and products were determined an Agilent 7890 GC-FID. The method parameters and column information are given below. Aliquots of the MOF reaction mixtures (10 μL) were removed at each time point, diluted with HPLC grade benzene (0.5 mM), filtered over celite, and characterized by GC-FID. Aliquots of homogeneous reaction mixtures (10 μL) were removed at each timepoint, passed through a silica-celite column with CH_2Cl_2 (0.5 mM), and characterized by GC-FID. Response factors (RF) were determined using quantitative ^{1}H NMR spectroscopy and GC-FID according to Equation S1. The RF values for meta and para products ($RF_{monoboryl}$) were determined by the averaging the RFs for each isomer.

$$\frac{mmol\ substrate\ or\ product}{mmol\ standard} = RF \times \frac{GC{-}FID\ Area\ of\ substrate\ or\ product}{GC{-}FID\ Area\ of\ standard} \quad (\textit{Eq. S1})$$

Turnover numbers (TONs) for the formation of borylated products were calculated according to Equation S2.

$$\frac{mmol_{meta}}{mmol_{Ir}} + \frac{mmol_{para}}{mmol_{Ir}} + \frac{2 \times mmol_{diboryl}}{mmol_{Ir}} = TON \quad (\textit{Eq. S2})$$

Meta to para selectivity (*m:p*) was calculated using Equation S3.

$$\frac{(GC{-}FID\ Area_{meta} \times RF_{monoboryl} + GC{-}FID\ Area_{diboryl} \times RF_{diboryl})}{GC{-}FID\ Area_{para} \times RF_{monoboryl}} : 1 = meta : para \quad (\textit{Eq. S3})$$

Yield was calculated for each reaction aliquot according to Equation S4.

$$\frac{mmol_{meta} + mmol_{para} + mmol_{diboryl}}{starting\ mmol\ of\ substrate} \times 100\% = Yield \quad (\textit{Eq. S4})$$

Mass balance was calculated for each reaction aliquot according to Equation S5.

$$\frac{mmol\ substrate + mmol\ products}{starting\ mmols\ of\ substrate} \times 100\% = Mass\ Balance \quad (\textit{Eq. S5})$$

Column Information

J&W HP-5 GC Column, 30 m, 0.32 mm, 0.25 μm, 7-inch cage.

Method Parameters

Column flow: 5 mL/min
Initial Column Temp: 60 °C
Ramp 1: 10 °C/min to 130 °C; Hold time: 5 min
Ramp 2: 10 °C/min to 180 °C
Ramp 3: 35 °C/min to 300 °C; Hold time: 10 min

Table S4. Response factor tables for substrates.

Compound:	*t*BuPh		PhTIPS	
	HMB	*t*BuPh	TMB	PhTIPS
Mol Ratio (Quant. ^{1}H NMR)	1.00	0.95	1.00	0.11
GC Retention Time (min)	9.53	3.16	8.66	12.53
GC-FID Integration (a.u.)	112068	83058	40719	10805
Response Factor	1.00	1.28	1.00	0.43

Compound:	PhOTMS		PhOTBDMS	
	HMB	PhOTMS	HMB	PhOTBDMS
Mol Ratio (Quant. ^{1}H NMR)	1.00	0.71	1.00	0.62
GC Retention Time (min)	9.53	3.16	9.53	6.65
GC-FID Integration (a.u.)	117013	57407	111038	69649
Response Factor	1.00	1.44	1.00	1.00

Compound:	PhOTIPS	
	HMB	PhOTIPS
Mol Ratio (Quant. ^{1}H NMR)	1.00	1.13
GC Retention Time (min)	9.53	13.52
GC-FID Integration (a.u.)	51015	70940
Response Factor	1.00	0.81

Compound:	BnTIPS		BnOTIPS	
	TMB	BnTIPS	TMB	BnOTIPS
Mol Ratio (Quant. ^{1}H NMR)	1.00	1.76	1.00	3.27
GC Retention Time (min)	8.66	15.44	8.66	15.65
GC-FID Integration (a.u.)	3725	17017	9584	84603
Response Factor	1.00	0.39	1.00	0.37

Compound:	2-Me-PhOTIPS		2-iPr-PhOTIPS	
	HMB	2-Me-PhOTIPS	HMB	2-*i*Pr-PhOTIPS
Mol Ratio (Quant. ^{1}H NMR)	1.00	6.52	1.00	3.31
GC Retention Time (min)	9.54	15.37	9.53	16.64
GC-FID Integration (a.u.)	15771	130884	11258	54070
Response Factor	1.00	0.79	1.00	0.69

	2-MeO-PhOTIPS		2-CF_3-PhOTIPS	
Compound:	HMB	2-MeO-PhOTIPS	HMB	2-CF_3-PhOTIPS
Mol Ratio (Quant. ^{1}H NMR)	1.72	1.53	1.00	0.89
GC Retention Time (min)	19.87	20.12	9.53	14.19
GC-FID Integration (a.u.)	17292	14681	79908	83868
Response Factor	1.30	1.36	1.00	0.85

	2-Cl-PhOTIPS		2-Br-PhOTIPS	
Compound:	HMB	2-Cl-PhOTIPS	HMB	2-Br-PhOTIPS
Mol Ratio (Quant. ^{1}H NMR)	1.00	2.57	1.00	3.29
GC Retention Time (min)	9.53	16.68	9.53	17.69
GC-FID Integration (a.u.)	37964	117689	12771	50653
Response Factor	1.00	0.83	1.00	0.83

Table S5. Response factor tables for products.

2 (Toluene-Bpin)

Compound:	HMB	*meta*	*para*	*diboryl*
Mol Ratio (Quant. ^{1}H NMR)	1.00	2.22	1.09	0.06
GC Retention Time (min)	9.53	9.93	10.17	19.21
GC-FID Integration (a.u.)	56719	119309	59101	3767
Response Factor	1.00	1.06	1.05	0.90

3 (*t*BuPh-Bpin)

Compound:	HMB	*meta*	*para*	*diboryl*
Mol Ratio (Quant. ^{1}H NMR)	1.00	1.23	0.38	0.11
GC Retention Time (min)	9.53	14.78	15.81	19.54
GC-FID Integration (a.u.)	30790	49697	14941	3621
Response Factor	1.00	0.76	0.78	0.94

4 (PhTIPS-Bpin)

Compound:	TMB	*meta*	*para*	*diboryl*
Mol Ratio (Quant. ^{1}H NMR)	1.00	0.58	0.32	0.50
GC Retention Time (min)	8.66	19.16	19.65	20.59
GC-FID Integration (a.u.)	40719	59158	28712	24023
Response Factor	1.00	0.40	0.45	0.85

5 (PhOTMS-Bpin)

Compound:	HMB	*meta*	*para*	*diboryl*
Mol Ratio (Quant. ^{1}H NMR)	1.00	1.34	0.31	0.21
GC Retention Time (min)	9.53	15.55	16.46	19.66
GC-FID Integration (a.u.)	32842	50409	11150	4852
Response Factor	1.00	0.87	0.90	1.42

6 (PhOTBDMS-Bpin)

Compound:	HMB	*meta*	*para*	*diboryl*
Mol Ratio (Quant. ^{1}H NMR)	1.00	0.73	0.84	0.03
GC Retention Time (min)	9.53	18.10	18.57	20.23
GC-FID Integration (a.u.)	5399	4067	4569	147
Response Factor	1.00	0.97	0.99	1.10

7 (PhOTIPS-Bpin)

Compound:	HMB	*meta*	*para*	*diboryl*
Mol Ratio (Quant. ^{1}H NMR)	1.00	2.01	0.45	0.28
GC Retention Time (min)	9.53	19.36	19.78	20.86
GC-FID Integration (a.u.)	11400	19652	4094	1181
Response Factor	1.00	1.14	1.24	2.65

8 (BnTIPS-Bpin)

Compound:	TMB	*meta*	*para*	*diboryl*
Mol Ratio (Quant. ^{1}H NMR)	1.00	1.13	0.66	0.12
GC Retention Time (min)	8.66	19.71	20.05	21.13
GC-FID Integration (a.u.)	3725	8687	4224	391
Response Factor	1.00	0.48	0.58	1.14

9 (BnOTIPS-Bpin)

Compound:	TMB	*meta*	*para*	*diboryl*
Mol Ratio (Quant. ^{1}H NMR)	1.00	2.67	1.21	0.25
GC Retention Time (min)	8.66	19.81	20.10	21.32
GC-FID Integration (a.u.)	9584	37148	17843	1450
Response Factor	1.00	0.69	0.65	1.65

10 (2-Me-PhOTIPS-Bpin)

Compound:	HMB	*a*	*b*
Mol Ratio (Quant. ^{1}H NMR)	1.00	1.51	0.53
GC Retention Time (min)	9.54	19.52	20.04
GC-FID Integration (a.u.)	15771	20774	6960
Response Factor	1.00	1.14	1.20

11 (2-*i*Pr-PhOTIPS-Bpin)

Compound:	HMB	*a*	*b*
Mol Ratio (Quant. ^{1}H NMR)	1.00	0.54	0.93
GC Retention Time (min)	9.53	19.70	20.03
GC-FID Integration (a.u.)	11258	5531	9610
Response Factor	1.00	1.10	1.09

12 (2-MeO-PhOTIPS-Bpin)

Compound:	HMB	*a*	*b*
Mol Ratio (Quant. ^{1}H NMR)	1.00	1.72	1.53
GC Retention Time (min)	9.53	19.87	20.12
GC-FID Integration (a.u.)	13039	17292	14681
Response Factor	1.00	1.30	1.36

13 (2-CF_3-PhOTIPS-Bpin)

Compound:	HMB	*a*	*b*
Mol Ratio (Quant. ^{1}H NMR)	1.00	0.59	2.24
GC Retention Time (min)	9.53	19.16	19.68
GC-FID Integration (a.u.)	7557	4036	15125
Response Factor	1.00	1.10	1.12

14 (2-Cl-PhOTIPS-Bpin)

Compound:	HMB	*a*	*b*
Mol Ratio (Quant. ^{1}H NMR)	1.00	2.80	1.85
GC Retention Time (min)	9.53	19.80	20.26
GC-FID Integration (a.u.)	37964	84610	48730
Response Factor	1.00	1.26	1.44

15 (2-Br-PhOTIPS-Bpin)

Compound:	HMB	*a*	*b*
Mol Ratio (Quant. ^{1}H NMR)	1.00	2.45	1.82
GC Retention Time (min)	9.53	20.06	20.52
GC-FID Integration (a.u.)	12771	19129	12279
Response Factor	1.00	1.64	1.89

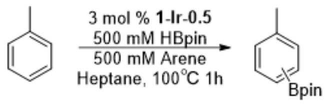

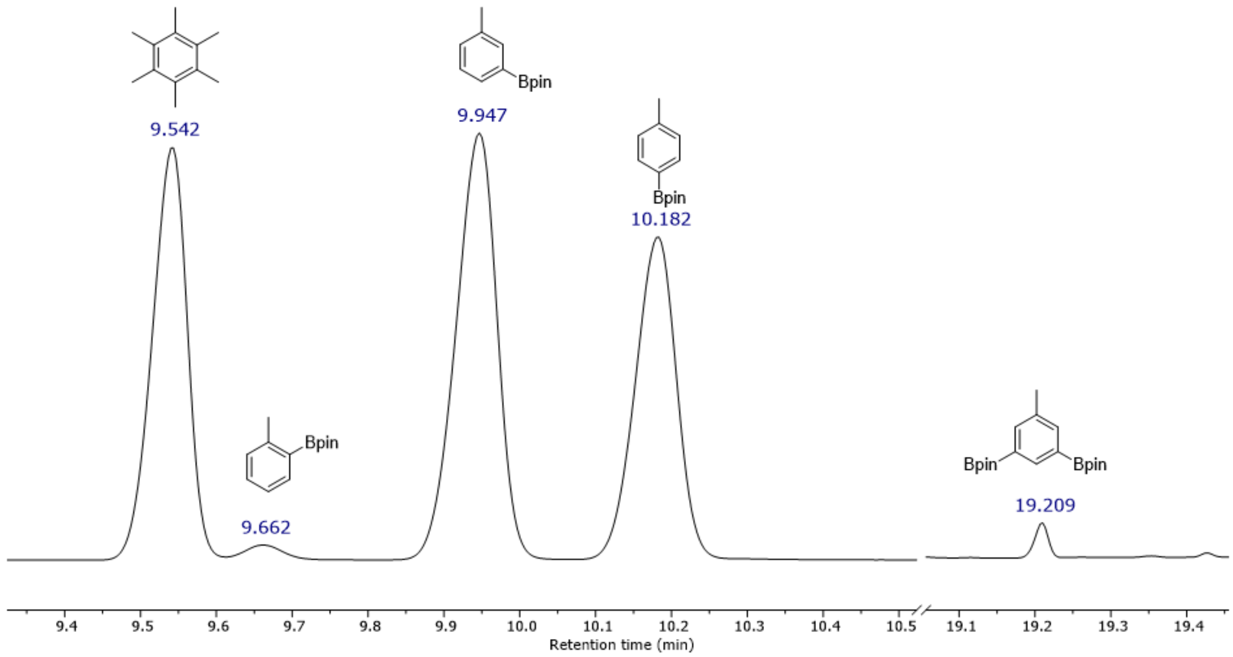

GC-FID reaction mixture ratio at t=1 h:

Compound	Retention Time (min)	Integration (a.u.)	mmol wrt standard
Hexamethylbenzene	9.542	37618	1.000
Ortho borylated	9.662	1378	0.039
Meta borylated	9.947	45761	1.289
Para borylated	10.182	34607	0.975
Diborylated	19.209	1259	0.035

Mass balance was unobtainable due to toluene eluting with solvent (benzene).

Figure S40. GC-FID chromatogram for the C–H borylation of toluene (500 mM) using **1-Ir-0.5** and HBpin (500 mM) in heptane.

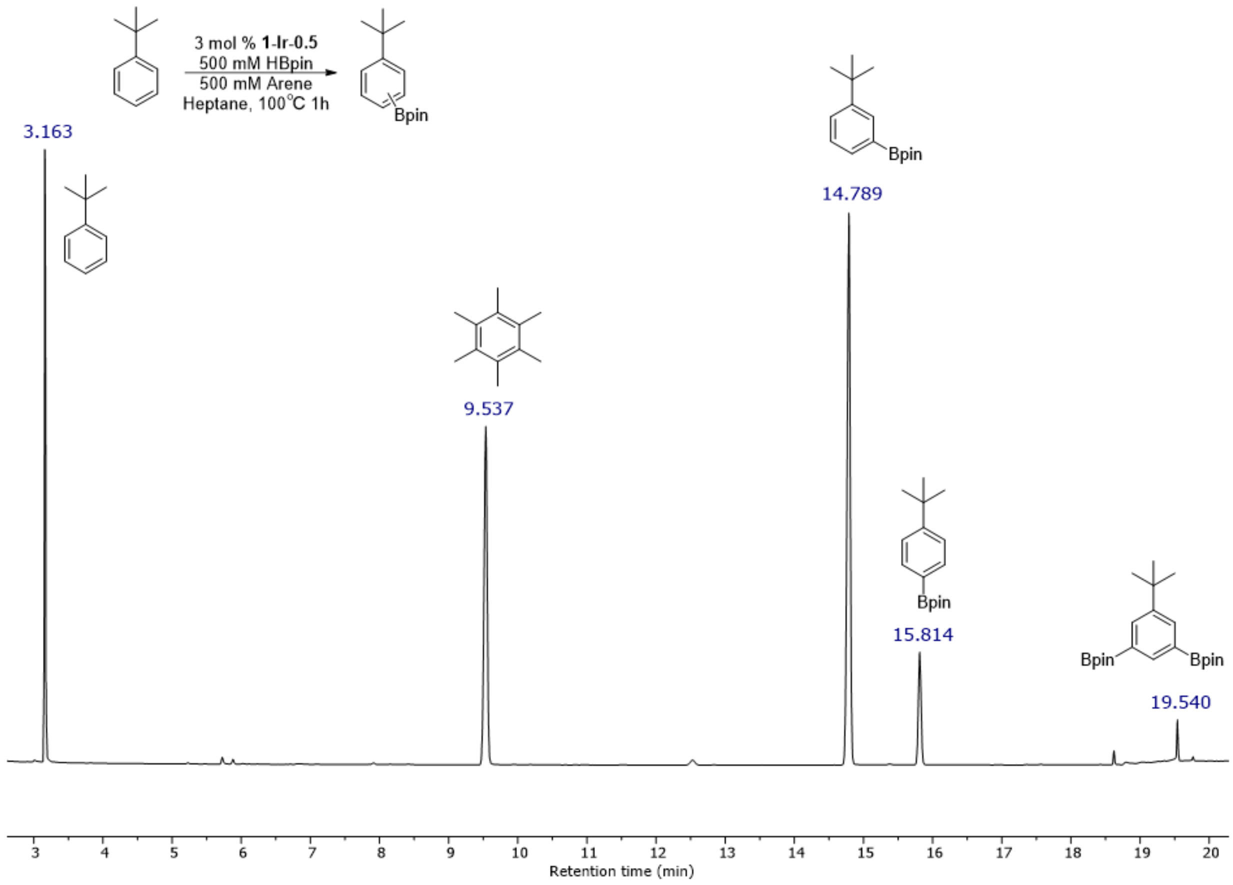

GC-FID reaction mixture ratio at t=1 h:

Compound	Retention Time (min)	Integration (a.u.)	mmol wrt standard
tBuPh	3.163	20926	0.892
Hexamethylbenzene	9.537	30029	1.000
Meta borylated	14.789	47840	1.227
Para borylated	15.814	8903	0.228
Diborylated	19.54	1334	0.042

Mass balance: 58.6%

Figure S41. GC-FID chromatogram for the C–H borylation of *tert*-butylbenzene (500 mM) using **1-Ir-0.5** and HBpin (500 mM) in heptane.

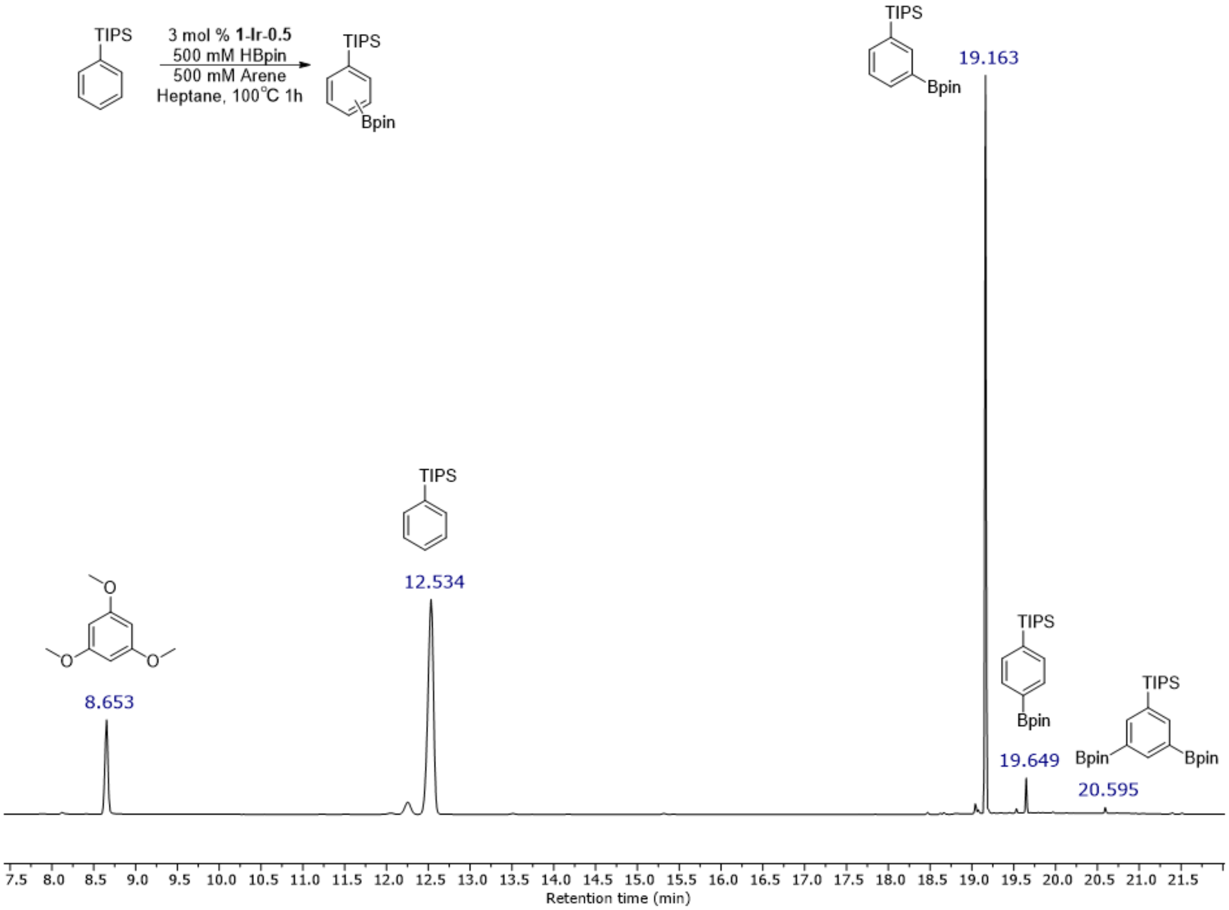

GC-FID reaction mixture ratio at t=1 h:

Compound	Retention Time (min)	Integration (a.u.)	mmol wrt standard
Trimethoxybenzene	8.653	15942	1.000
PhTIPS	12.534	66297	1.788
Meta borylated	19.163	55966	1.510
Para borylated	19.649	2388	0.064
Diborylated	20.595	435	0.023

Mass balance: 92.5%

Figure S42. GC-FID chromatogram for the C–H borylation of phenyl(triisopropyl)silane (PhTIPS) (500 mM) using **1-Ir-0.5** and HBpin (500 mM) in heptane.

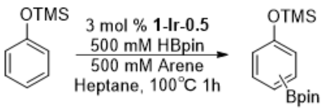

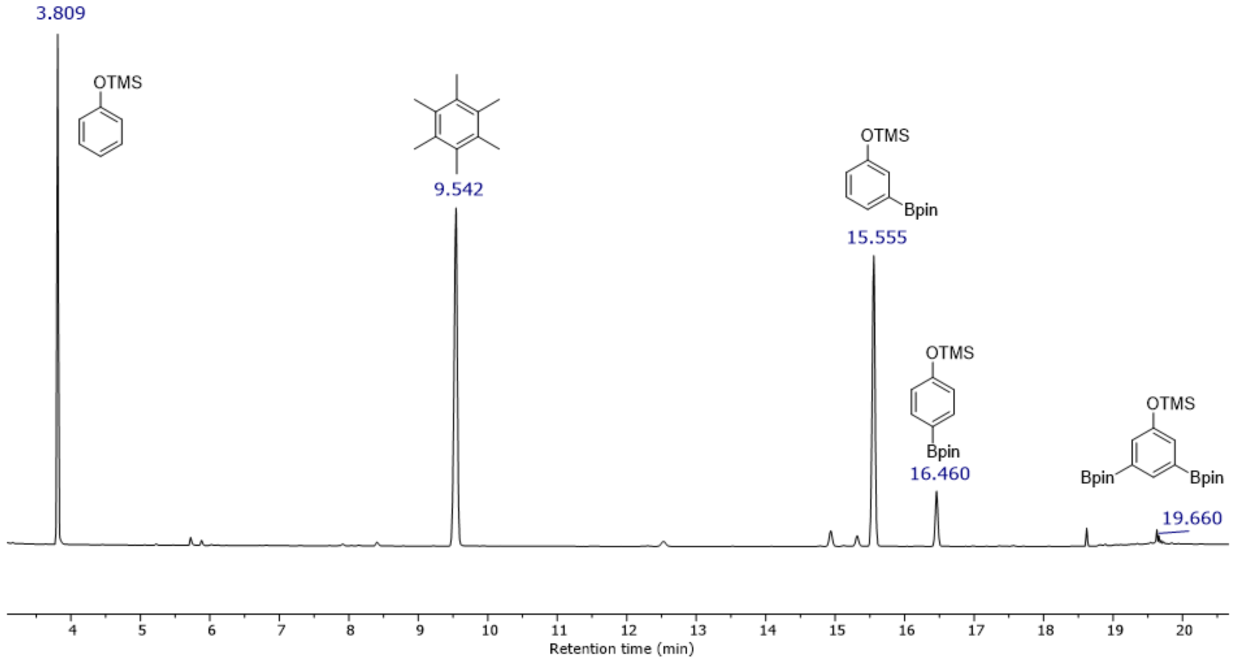

GC-FID reaction mixture ratio at t=1 h:

Compound	Retention Time (min)	Integration (a.u.)	mmol wrt standard
PhOTMS	3.809	27902	0.956
Hexamethylbenzene	9.542	42012	1
Meta borylated	15.555	32052	0.679
Para borylated	16.46	5539	0.117
Diborylated	19.66	210	0.007

Mass balance: 43.1%

Figure S43. GC-FID chromatogram for the C–H borylation of phenoxy(trimethyl)silane (PhOTMS) (500 mM) using **1-Ir-0.5** and HBpin (500 mM) in heptane.

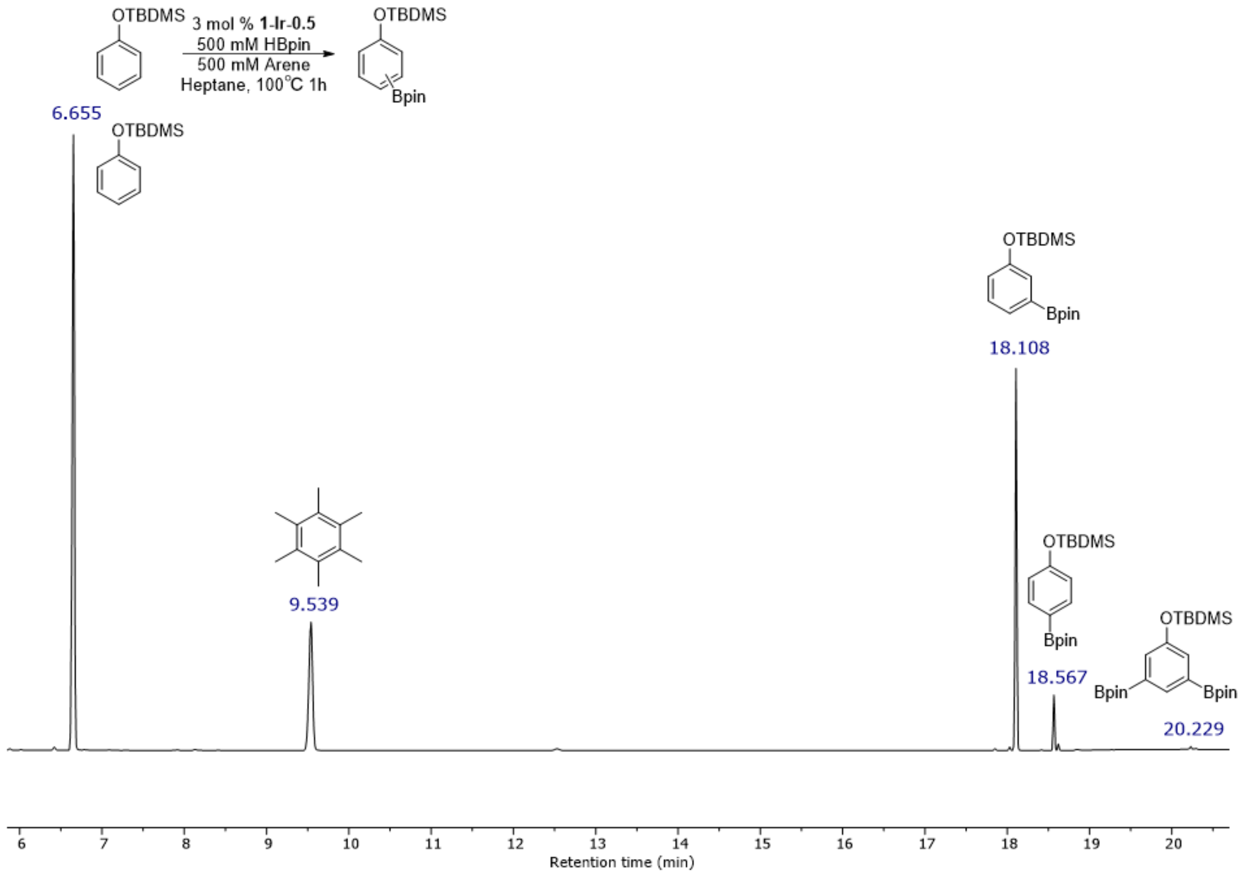

GC-FID reaction mixture ratio at t=1 h:

Compound	Retention Time (min)	Integration (a.u.)	mmol wrt standard
PhOTBDMS	6.655	92910	2.845
Hexamethylbenzene	9.539	32653	1.000
Meta borylated	18.108	44134	1.325
Para borylated	18.567	5511	0.165
Diborylated	20.229	240	0.008

Mass balance: 100.3%

Figure S44. GC-FID chromatogram for the C–H borylation of phenoxy(*tert*-butyldimethyl)silane (PhOTBDMS) (500 mM) using **1-Ir-0.5** and HBpin (500 mM) in heptane.

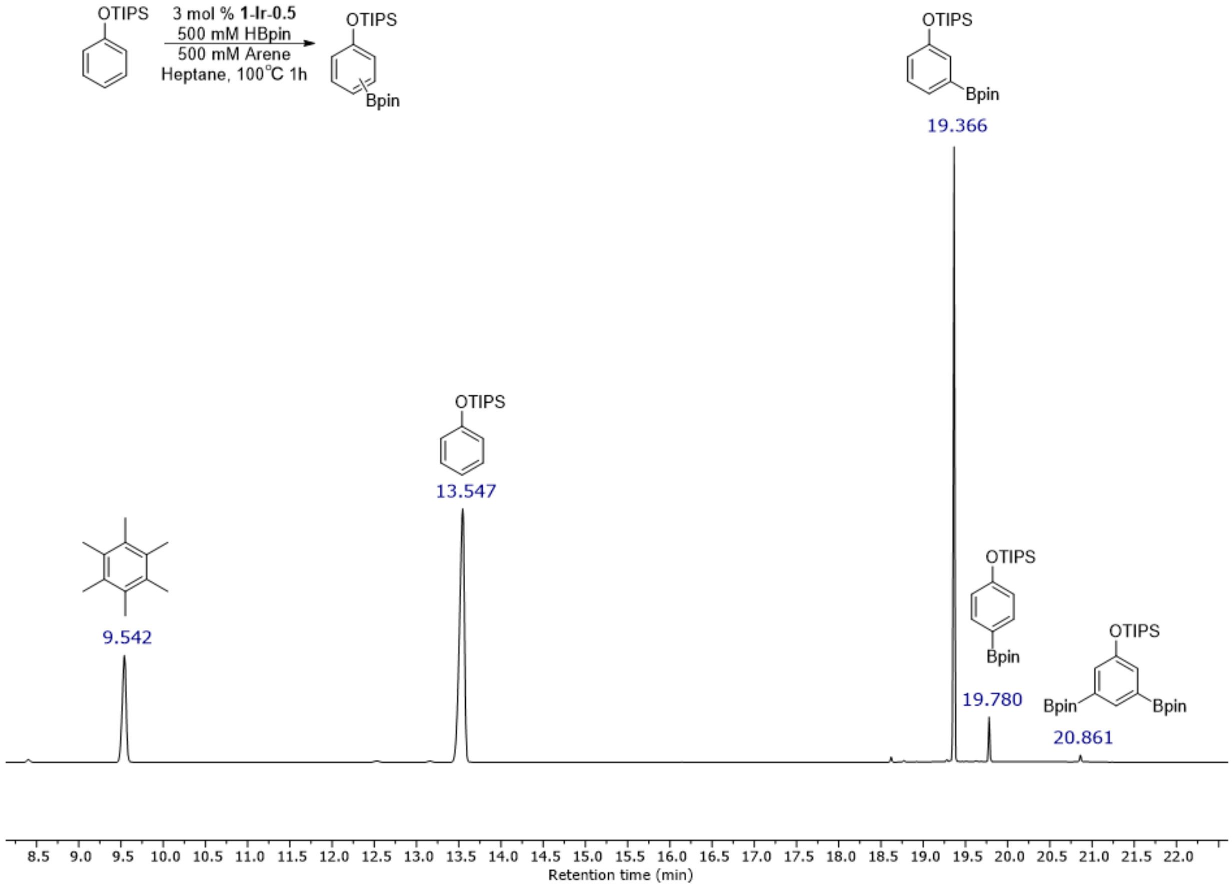

GC-FID reaction mixture ratio at t=1 h:

Compound	Retention Time (min)	Integration (a.u.)	mmol wrt standard
Hexamethylbenzene	9.542	43510	1.000
PhOTIPS	13.547	130178	2.423
Meta borylated	19.366	86489	2.365
Para borylated	19.78	5962	0.163
Diborylated	20.861	911	0.055

Mass balance: 99.4%

Figure S45. GC-FID chromatogram for the C–H borylation of phenoxy(triisopropyl)silane (PhOTIPS) (500 mM) using **1-Ir-0.5** and HBpin (500 mM) in heptane.

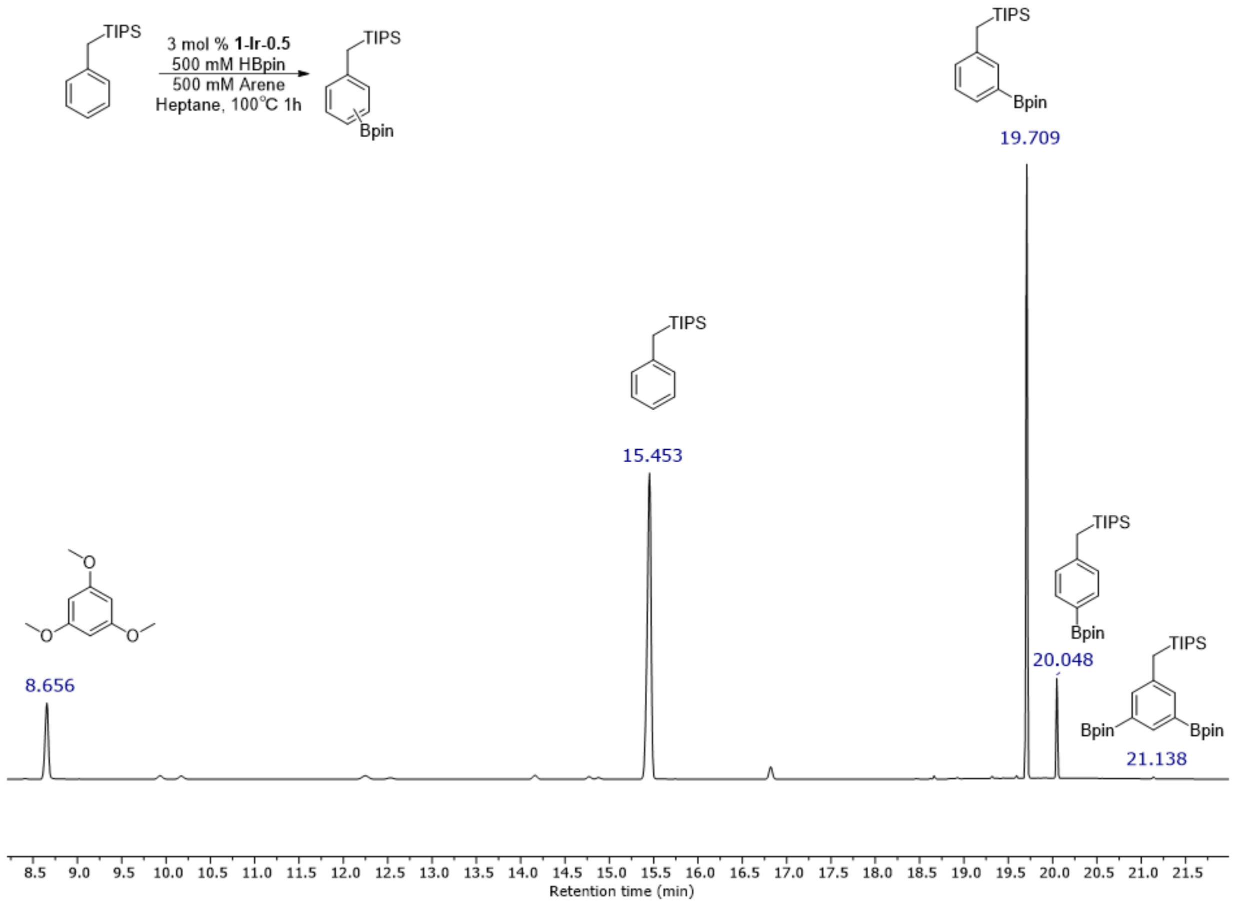

GC-FID reaction mixture ratio at t=1 h:

Compound	Retention Time (min)	Integration (a.u.)	mmol wrt standard
Trimethoxybenzene	8.656	19652	1.000
BnTIPS	15.453	92963	1.845
Meta borylated	19.709	69264	1.833
Para borylated	20.048	11258	0.298
Diborylated	21.138	276	0.016

Mass balance: 106.4%

Figure S46. GC-FID chromatogram for the C–H borylation of benzyl(triisopropyl)silane (BnTIPS) (500 mM) using **1-Ir-0.5** and HBpin (500 mM) in heptane.

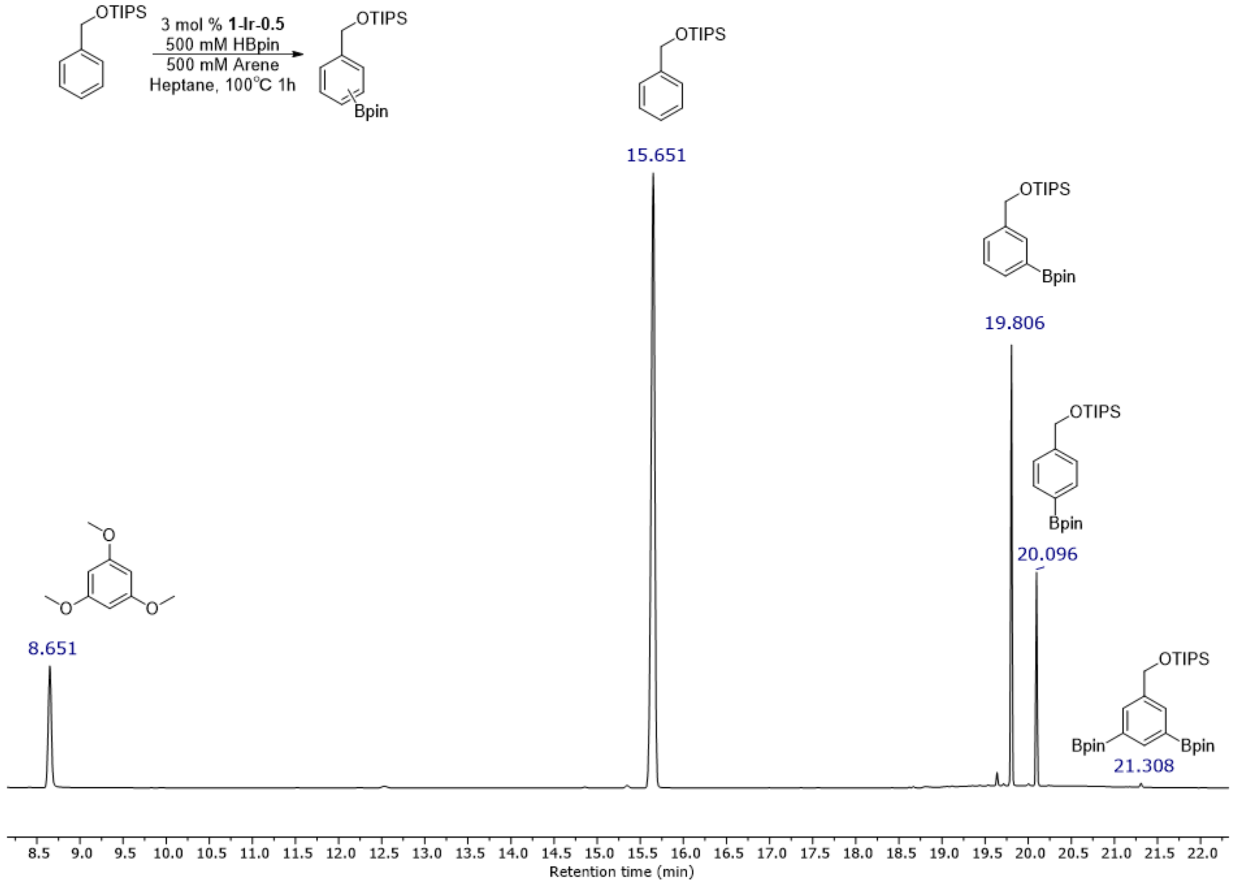

GC-FID reaction mixture ratio at t=1h:

Compound	Retention Time (min)	Integration (a.u.)	mmol wrt standard
Trimethoxybenzene	8.651	12411	1.000
BnOTIPS	15.651	73404	2.188
Meta borylated	19.806	19205	1.037
Para borylated	20.096	9476	0.512
Diborylated	21.308	228	0.030

Mass balance: 101.3%

Figure S47. GC-FID chromatogram for the C–H borylation of benzyl(triisopropyl)silyl ether (BnOTIPS) (500 mM) using **1-Ir-0.5** and HBpin (500 mM) in heptane.

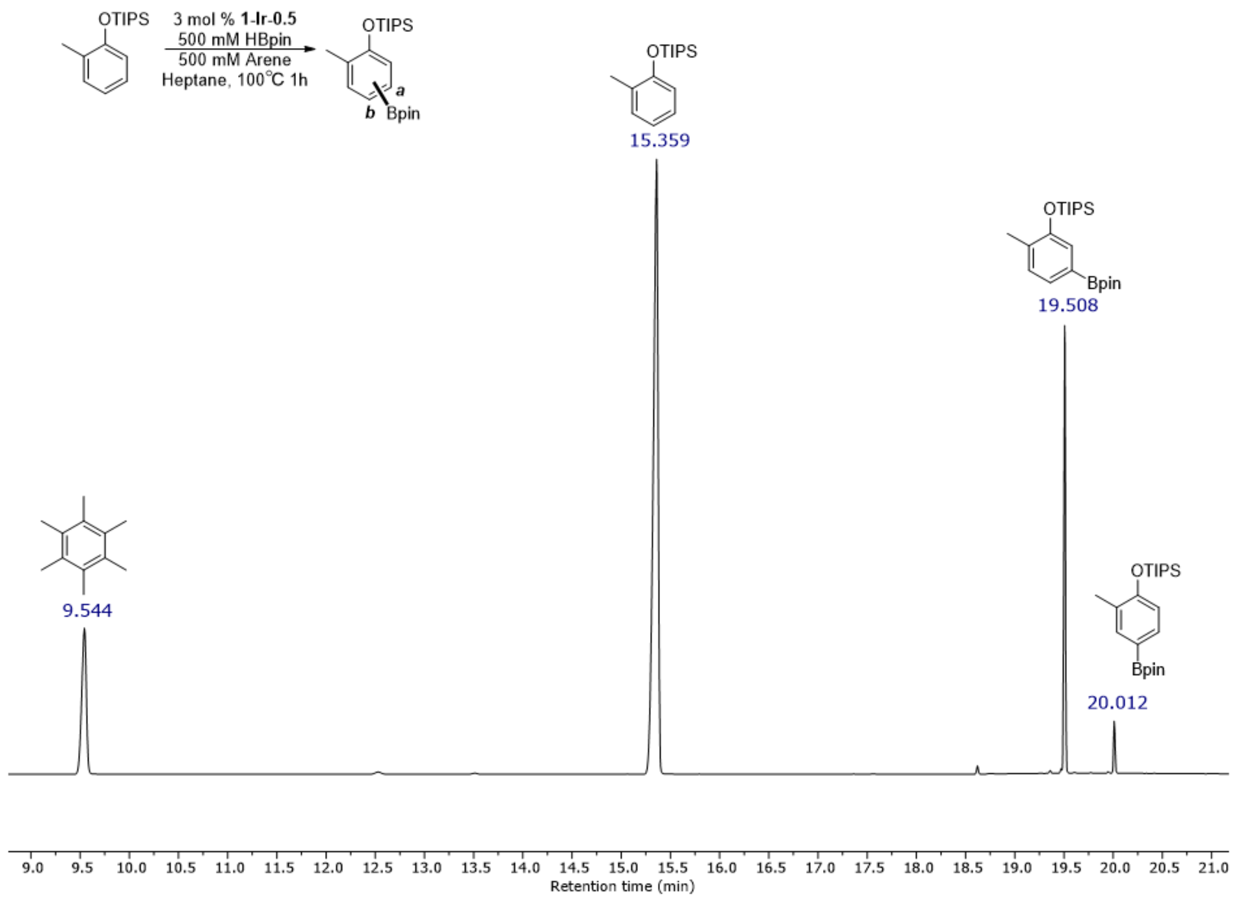

GC-FID reaction mixture ratio at t=1 h:

Compound	Retention Time (min)	Integration (a.u.)	mmol with respect to standard
Hexamethylbenzene	9.544	47190	1.000
2-Me-PhOTIPS	15.359	204849	3.429
A isomer	19.508	49299	1.222
B isomer	20.012	5616	0.139

Mass balance: 98.3%

Figure S48. GC-FID chromatogram for the C–H borylation of 2-methyl-phenoxy(triisopropyl)silane (2-Me-PhOTIPS) (500 mM) using **1-Ir-0.5** and HBpin (500 mM) in heptane.

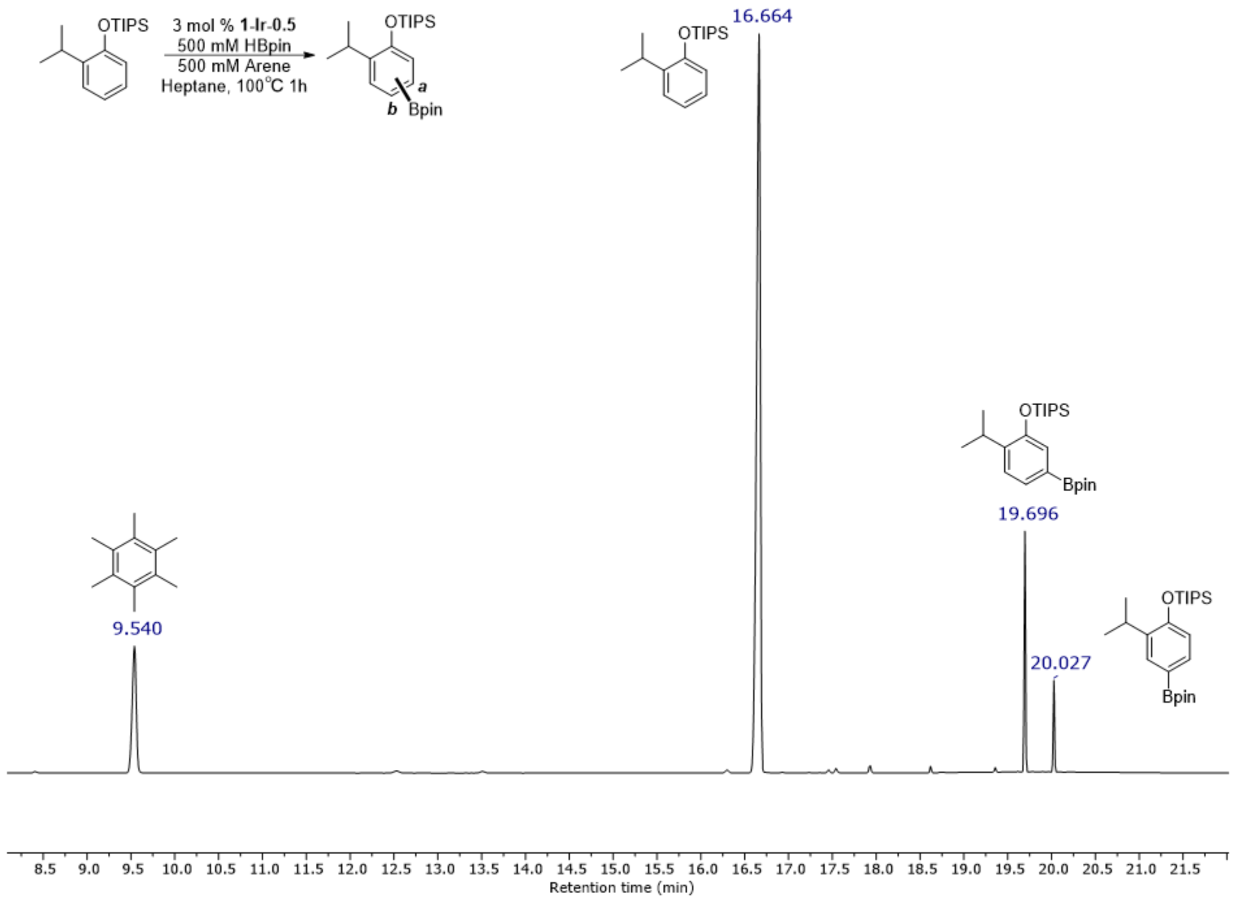

GC-FID reaction mixture ratio at t=1 h:

Compound	Retention Time (min)	Integration (a.u.)	mmol wrt standard
Hexamethylbenzene	9.540	40228	1.000
2-iPr-PhOTIPS	16.664	218455	3.747
A isomer	19.696	26073	0.713
B isomer	20.027	9680	0.265

Mass balance: 95.6%.

Figure S49. GC-FID chromatogram for the C–H borylation of 2-isopropyl-phenoxy(triisopropyl)silane (2-iPr-PhOTIPS) (500 mM) using **1-Ir-0.5** and HBpin (500 mM) in heptane.

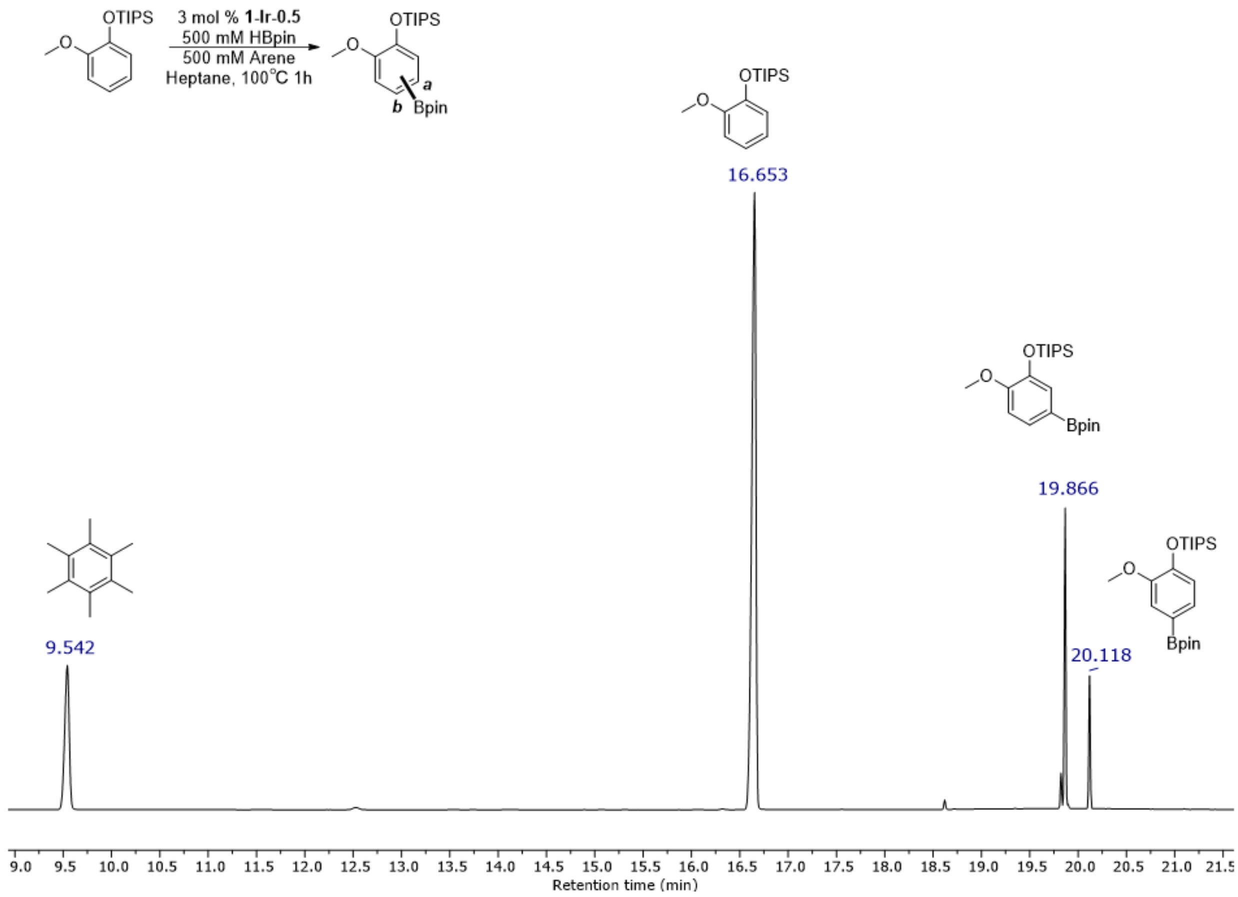

GC-FID reaction mixture ratio at t=1 h:

Compound	Retention Time (min)	Integration (a.u.)	mmol wrt standard
Hexamethylbenzene	9.542	43858	1.000
2-MeO-PhOTIPS	16.653	174708	3.027
A isomer	19.866	34435	1.044
B isomer	20.118	13928	0.422

Mass balance: 94.2%

Figure S50. GC-FID chromatogram for the C–H borylation of 2-methoxy-phenoxy(triisopropyl)silane (2-MeO-PhOTIPS) (500 mM) using **1-Ir-0.5** and HBpin (500 mM) in heptane.

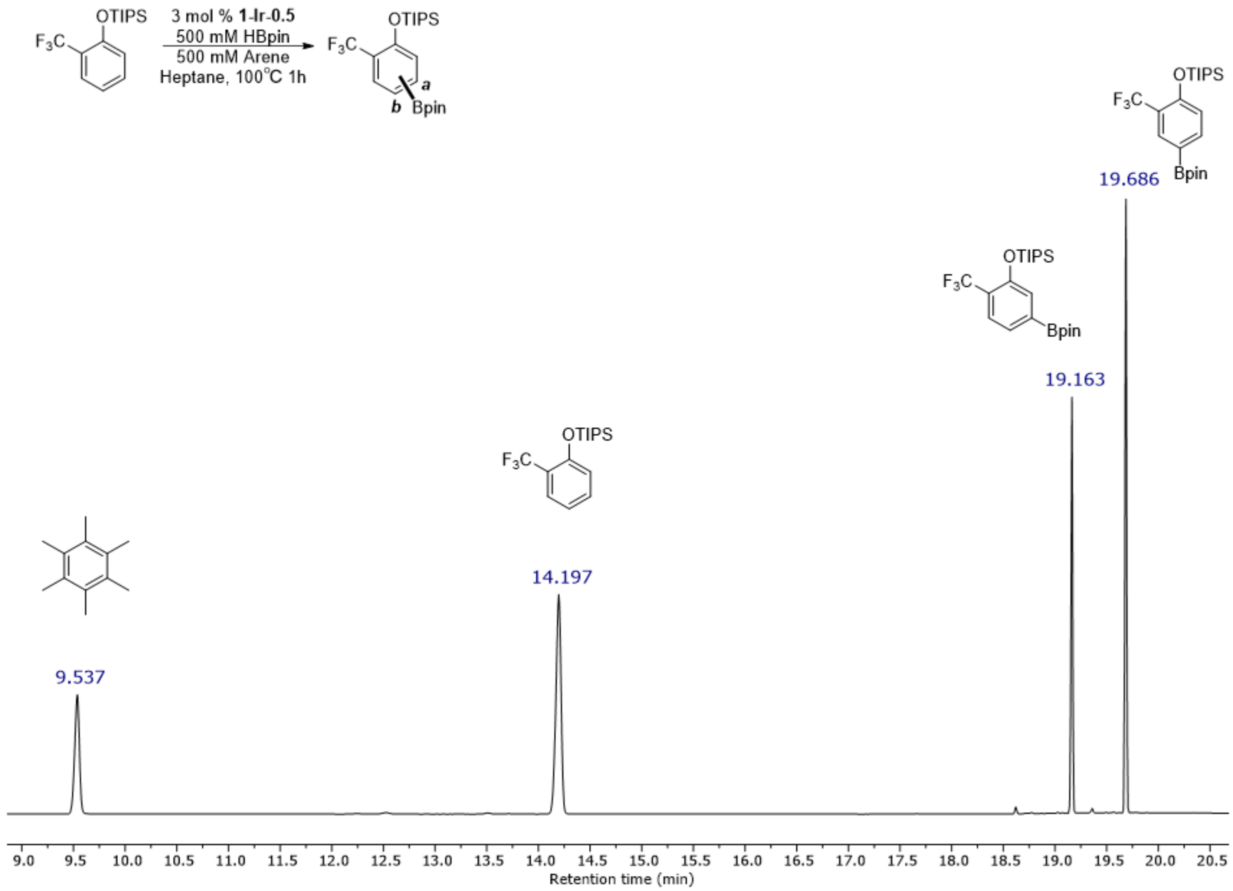

GC-FID reaction mixture ratio at t=1 h:

Compound	Retention Time (min)	Integration (a.u.)	mmol wrt standard
Hexamethylbenzene	9.537	30507	1.000
2-CF_3-PhOTIPS	14.197	61345	1.709
A isomer	19.163	37598	1.368
B isomer	19.686	53289	1.939

Mass balance: 101.7%

Figure S51. GC-FID chromatogram for the C–H borylation of 2-trifluoromethyl-phenoxy(triisopropyl)silane (2-CF_3-PhOTIPS) (500 mM) using **1-Ir-0.5** and HBpin (500 mM) in heptane.

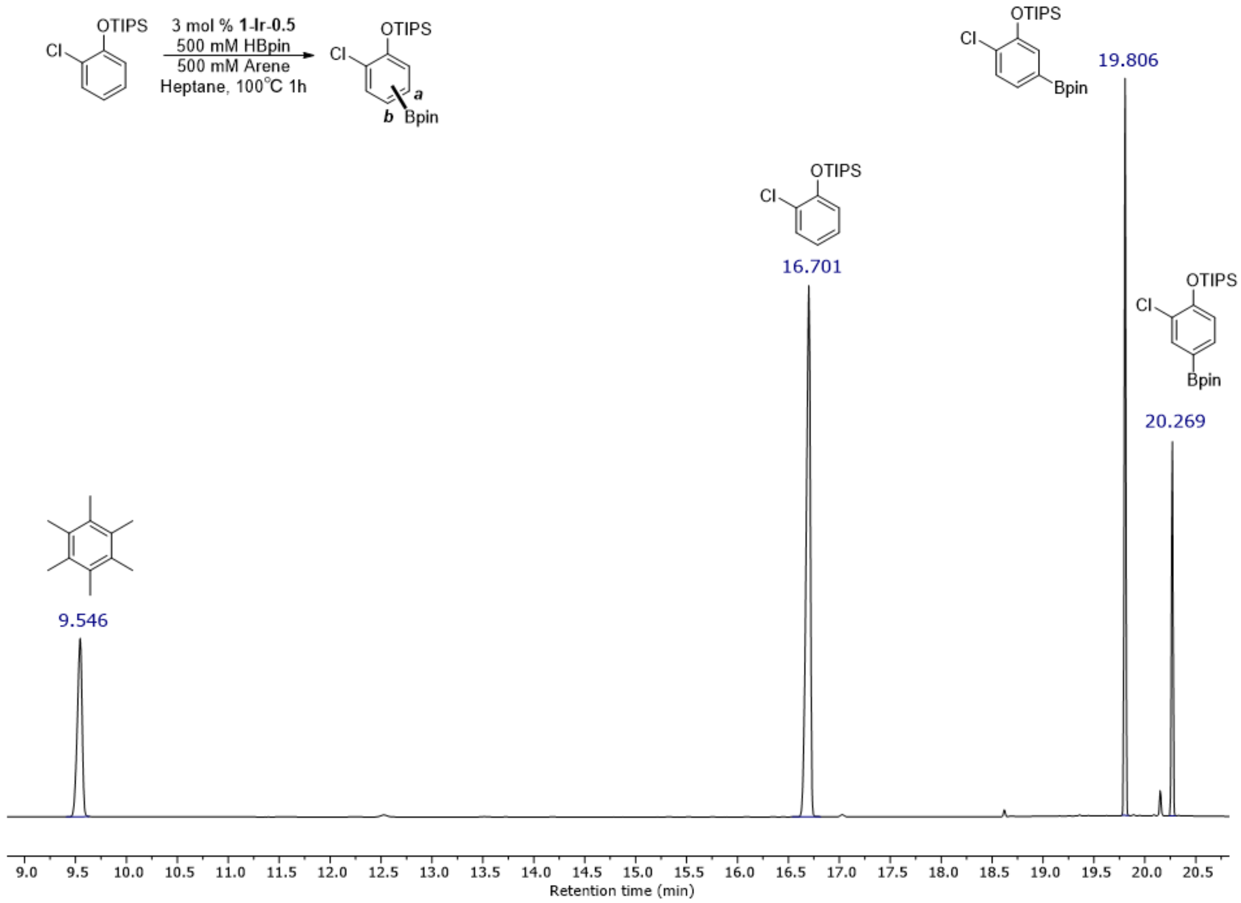

GC-FID reaction mixture ratio at t=1 h:

Compound	Retention Time (min)	Integration (a.u.)	mmol wrt standard
Hexamethylbenzene	9.546	53701	1.000
2-Cl-PhOTIPS	16.701	138260	2.137
A isomer	19.806	74046	1.861
B isomer	20.269	37160	0.934

Mass balance: 98.7%

Figure S52. GC-FID chromatogram for the C–H borylation of 2-chloro-phenoxy(triisopropyl)silane (2-Cl-PhOTIPS) (500 mM) using **1-Ir-0.5** and HBpin (500 mM) in heptane.

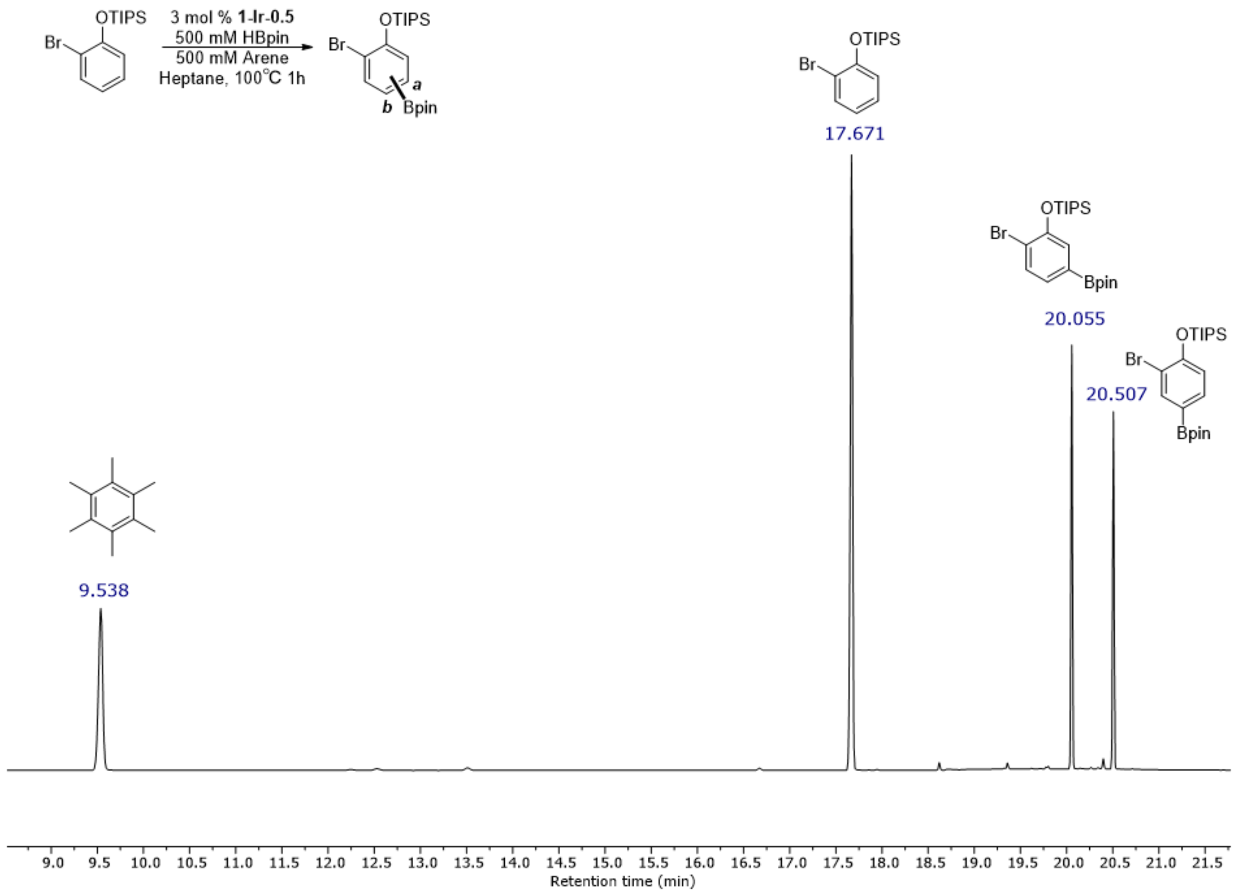

GC-FID reaction mixture ratio at t=1 h:

Compound	Retention Time (min)	Integration (a.u.)	mmol wrt standard
Hexamethylbenzene	9.538	34019	1.000
2-Br-PhOTIPS	17.671	71507	1.745
A isomer	20.055	30768	1.601
B isomer	20.507	25823	1.344

Mass balance: 94.2%

Figure S53. GC-FID chromatogram for the C–H borylation of 2-bromo-phenoxy(triisopropyl)silane (2-Br-PhOTIPS) (500 mM) using **1-Ir-0.5** and HBpin (500 mM) in heptane.

References

(1) Gonzalez, M. I.; Bloch, E. D.; Mason, J. A.; Teat, S. J.; Long, J. R. Single-Crystal-to-Single-Crystal Metalation of a Metal–Organic Framework: A Route toward Structurally Well-Defined Catalysts. *Inorg Chem* 2015, *54* (6), 2995–3005. https://doi.org/10.1021/acs.inorgchem.5b00096.

(2) Manna, K.; Zhang, T.; Greene, F. X.; Lin, W. Bipyridine- and Phenanthroline-Based Metal–Organic Frameworks for Highly Efficient and Tandem Catalytic Organic Transformations via Directed C–H Activation. *J Am Chem Soc* 2015, *137* (7), 2665–2673. https://doi.org/10.1021/ja512478y.

(3) Manna, K.; Zhang, T.; Lin, W. Postsynthetic Metalation of Bipyridyl-Containing Metal-Organic Frameworks for Highly Efficient Catalytic Organic Transformations. *J Am Chem Soc* 2014, *136* (18), 6566–6569. https://doi.org/10.1021/ja5018267.

(4) Waki, M.; Maegawa, Y.; Hara, K.; Goto, Y.; Shirai, S.; Yamada, Y.; Mizoshita, N.; Tani, T.; Chun, W.-J.; Muratsugu, S.; Tada, M.; Fukuoka, A.; Inagaki, S. A Solid Chelating Ligand: Periodic Mesoporous Organosilica Containing 2,2′-Bipyridine within the Pore Walls. *J Am Chem Soc* 2014, *136* (10), 4003–4011. https://doi.org/10.1021/ja4131609.

(5) Hilliard, J. S.; Murray, D. J.; Saha, A.; Goldberger, J. E.; Wade, C. R. MOF-Supported Diphosphine Ligands for Iridium-Catalyzed C–H Borylation of Arenes. *Inorg Chem* 2025, *64* (14), 7127–7136. https://doi.org/10.1021/acs.inorgchem.5c00523.

(6) Maegawa, Y.; Inagaki, S. Iridium–Bipyridine Periodic Mesoporous Organosilica Catalyzed Direct C–H Borylation Using a Pinacolborane. *Dalton Transactions* 2015, *44* (29), 13007–13016. https://doi.org/10.1039/C5DT00239G.

(7) Boller, T. M.; Murphy, J. M.; Hapke, M.; Ishiyama, T.; Miyaura, N.; Hartwig, J. F. Mechanism of the Mild Functionalization of Arenes by Diboron Reagents Catalyzed by Iridium Complexes. Intermediacy and Chemistry of Bipyridine-Ligated Iridium Trisboryl Complexes. *J Am Chem Soc* 2005, *127* (41), 14263–14278. https://doi.org/10.1021/ja053433g.

(8) Neese, F.; Wennmohs, F.; Becker, U.; Riplinger, C. The ORCA Quantum Chemistry Program Package. *J Chem Phys* 2020, *152* (22). https://doi.org/10.1063/5.0004608.

(9) Neese, F. Software Update: The ORCA Program System—Version 5.0. *WIREs Computational Molecular Science* 2022, *12* (5). https://doi.org/10.1002/wcms.1606.

(10) Bannwarth, C.; Ehlert, S.; Grimme, S. GFN2-XTB—An Accurate and Broadly Parametrized Self-Consistent Tight-Binding Quantum Chemical Method with

Multipole Electrostatics and Density-Dependent Dispersion Contributions. *J Chem Theory Comput* 2019, *15* (3), 1652–1671. https://doi.org/10.1021/acs.jctc.8b01176.

(11) Denysenko, D.; Grzywa, M.; Tonigold, M.; Streppel, B.; Krkljus, I.; Hirscher, M.; Mugnaioli, E.; Kolb, U.; Hanss, J.; Volkmer, D. Elucidating Gating Effects for Hydrogen Sorption in MFU-4-Type Triazolate-Based Metal-Organic Frameworks Featuring Different Pore Sizes. *Chem. Eur. J.* 2011, *17* (6), 1837–1848. https://doi.org/10.1002/chem.201001872.

(12) Grimme, S.; Hansen, A.; Ehlert, S.; Mewes, J.-M. R2SCAN-3c: A “Swiss Army Knife” Composite Electronic-Structure Method. *J Chem Phys* 2021, *154* (6). https://doi.org/10.1063/5.0040021.

(13) Ásgeirsson, V.; Birgisson, B. O.; Bjornsson, R.; Becker, U.; Neese, F.; Riplinger, C.; Jónsson, H. Nudged Elastic Band Method for Molecular Reactions Using Energy-Weighted Springs Combined with Eigenvector Following. *J Chem Theory Comput* 2021, *17* (8), 4929–4945. https://doi.org/10.1021/acs.jctc.1c00462.

(14) Bowling, P. E.; Dasgupta, S.; Herbert, J. M. Eliminating Imaginary Vibrational Frequencies in Quantum-Chemical Cluster Models of Enzymatic Active Sites. *J Chem Inf Model* 2024, *64* (9), 3912–3922. https://doi.org/10.1021/acs.jcim.4c00221.

(15) Bursch, M.; Mewes, J.; Hansen, A.; Grimme, S. Best-Practice DFT Protocols for Basic Molecular Computational Chemistry. *Angewandte Chemie International Edition* 2022, *61* (42). https://doi.org/10.1002/anie.202205735.

(16) Weigend, F.; Ahlrichs, R. Balanced Basis Sets of Split Valence, Triple Zeta Valence and Quadruple Zeta Valence Quality for H to Rn: Design and Assessment of Accuracy. *Physical Chemistry Chemical Physics* 2005, *7* (18), 3297. https://doi.org/10.1039/b508541a.

(17) Mardirossian, N.; Head-Gordon, M. ΩB97M-V: A Combinatorially Optimized, Range-Separated Hybrid, Meta-GGA Density Functional with VV10 Nonlocal Correlation. *J Chem Phys* 2016, *144* (21). https://doi.org/10.1063/1.4952647.

(18) Weigend, F. Accurate Coulomb-Fitting Basis Sets for H to Rn. *Physical Chemistry Chemical Physics* 2006, *8* (9), 1057. https://doi.org/10.1039/b515623h.

(19) Andrae, D.; Häußermann, U.; Dolg, M.; Stoll, H.; Preuß, H. Energy-Adjusted Ab Initio Pseudopotentials for the Second and Third Row Transition Elements. *Theor Chim Acta* 1990, *77* (2), 123–141. https://doi.org/10.1007/BF01114537.

(20) Barone, V.; Cossi, M. Quantum Calculation of Molecular Energies and Energy Gradients in Solution by a Conductor Solvent Model. *J Phys Chem A* 1998, *102* (11), 1995–2001. https://doi.org/10.1021/jp9716997.

(21) Garcia-Ratés, M.; Neese, F. Efficient Implementation of the Analytical Second Derivatives of Hartree–Fock and Hybrid DFT Energies within the Framework of the Conductor-like Polarizable Continuum Model. *J Comput Chem* 2019, *40* (20), 1816–1828. https://doi.org/10.1002/jcc.25833.
